# Cabozantinib versus everolimus, nivolumab, axitinib, sorafenib and best supportive care: A network meta-analysis of progression-free survival and overall survival in second line treatment of advanced renal cell carcinoma

**DOI:** 10.1371/journal.pone.0184423

**Published:** 2017-09-08

**Authors:** Billy Amzal, Shuai Fu, Jie Meng, Johanna Lister, Helene Karcher

**Affiliations:** Decision Analytics and Value in Access, Analytica LA-SER, London, England; National Institute of Health, UNITED STATES

## Abstract

**Background:**

Relative effect of therapies indicated for the treatment of advanced renal cell carcinoma (aRCC) after failure of first line treatment is currently not known. The objective of the present study is to evaluate progression-free survival (PFS) and overall survival (OS) of cabozantinib compared to everolimus, nivolumab, axitinib, sorafenib, and best supportive care (BSC) in aRCC patients who progressed after previous VEGFR tyrosine-kinase inhibitor (TKI) treatment.

**Methodology & findings:**

Systematic literature search identified 5 studies for inclusion in this analysis. The assessment of the proportional hazard (PH) assumption between the survival curves for different treatment arms in the identified studies showed that survival curves in two of the studies did not fulfil the PH assumption, making comparisons of constant hazard ratios (HRs) inappropriate. Consequently, a parametric survival network meta-analysis model was implemented with five families of functions being jointly fitted in a Bayesian framework to PFS, then OS, data on all treatments. The comparison relied on data digitized from the Kaplan-Meier curves of published studies, except for cabozantinib and its comparator everolimus where patient level data were available. This analysis applied a Bayesian fixed-effects network meta-analysis model to compare PFS and OS of cabozantinib versus its comparators. The log-normal fixed-effects model displayed the best fit of data for both PFS and OS, and showed that patients on cabozantinib had a higher probability of longer PFS and OS than patients exposed to comparators. The survival advantage of cabozantinib increased over time for OS. For PFS the survival advantage reached its maximum at the end of the first year’s treatment and then decreased over time to zero.

**Conclusion:**

With all five families of distributions, cabozantinib was superior to all its comparators with a higher probability of longer PFS and OS during the analyzed 3 years, except with the Gompertz model, where nivolumab was preferred after 24 months.

## Introduction

Kidney cancer is relatively rare, accounting for 2.4% of all cancers globally (GLOBOCAN 2012 data) [1;2]. Data on the number of patients with renal cell carcinoma (RCC) who are alive at a certain point of time (disease prevalence) are scarce, mainly due to poor patient prognosis making incidence a more frequently used metric for cancer epidemiology [3]. Globally, the age-standardized incidence rate of RCC is 4.4 per 100,000 (data derived from population-based registries across the world; GLOBOCAN, Cancer Incidence in Five Continents series) [4]. In Europe, cancer registries report an age-standardized incidence rate of 12.1 per 100,000 for kidney cancer in general [2]. RCC is difficult to diagnose because it is generally asymptomatic or presents with unspecific symptoms at disease onset [5;6]. Delayed diagnosis results in a considerable proportion (30%) of patients presenting with advanced disease [6].

In Europe, standard of care for advanced renal cell carcinoma (aRCC) consists of targeted therapies that inhibit key signaling pathways involved in renal cell tumor genesis, specifically the vascular endothelial growth factor receptor (VEGFR) and the mammalian target of rapamycin (mTOR) pathways [7]. The European Association of Urology (EAU) recommends sunitinib or pazopanib as first line treatments for aRCC [8;9]. However, most patients progress on the first line therapy and require subsequent treatment. Median time to progression under the first line therapy is approximately 12 months [10]. Commonly used treatments after disease progression are everolimus, axitinib and sorafenib, and more recently also nivolumab and cabozantinib. From year 2005 onwards, targeted therapies, such as everolimus, axitinib and sorafenib have been recommended in National Comprehensive Cancer Network (NCCN), European Society for Medical Oncology (ESMO), and EAU guidelines for the second line treatment of aRCC [7;11;12]. Nivolumab and cabozantinib were added to recent guideline updates [8;9;12] after publication of results from the CheckMate025 and METEOR trials [13–15]. In our network we consider studies that either directly or indirectly contribute to the assessment of relative efficacy of cabozantinib compared to everolimus, axitinib, sorafenib, nivolumab or best supportive care.

Due to lack of head-to-head comparisons of therapies for aRCC after failure of VEGFR-therapy, the information regarding their comparative effectiveness is currently limited. Overall survival (OS) under nivolumab and cabozantinib have been compared in a recent network meta-analysis (NMA) [16] using data from the pivotal trials for each treatment [13;14]. Since the publication of this study, more mature OS results for cabozantinib were published [15]. No indirect treatment comparisons or NMAs were identified that compared cabozantinib with axitinib, sorafenib or best supportive care. The recent National Institute for Health and Care Excellence (NICE) single technology appraisal (STA) for nivolumab includes a NMA comparing nivolumab to axitinib, everolimus and best supportive care (BSC) [17]. While Wiecek & Karcher (2016) conducted a meta-analysis of full Kaplan-Meier (KM) data from publications [16], the nivolumab STA presented a meta-analysis of hazard ratios (HRs). NMAs based on HRs assume that the proportional hazard (PH) assumption holds for each pair of comparators, that is, that HR between treatment arms does not change over time. In the nivolumab NICE STA, the HR NMA approach was used without formally testing for violation of PHs assumption, and this was criticized by NICE. No additional analysis has been published using an alternative method. NMAs based on parametric curves compare the shape and scale parameters of each distribution fitted to the survival curves, and do not assume PHs between the pairwise comparators.

The objective of this study is to compare progression free survival (PFS) and OS and of cabozantinib to everolimus, nivolumab, axitinib, sorafenib and BSC by using the NMA method based on parametric survival curves as described by Ouwens et al. 2010 [18] and as used by Wiecek & Karcher (2016) in their analysis of cabozantinib and nivolumab OS relative gains [16]. Hence, this study will provide an update of the analysis done by Wiecek & Karcher (2016) on the nivolumab versus cabozantinib OS comparison using mature OS data for cabozantinib, while testing an additional distribution (log-normal). It will also provide the first comparison of cabozantinib OS versus other treatments in second line treatment of aRCC. Finally, to the authors’ knowledge, this study also provides the first PFS comparison of cabozantinib and its comparators using the NMA method based on parametric survival curves. These particular parametric survival distributions were chosen because they are typically required in decision analytic models of cost-effectiveness as part of the health technology assessment agency submissions, such as those for NICE.

## Materials and methods

### Study selection and data extraction

This systematic literature review aims to provide the evidence needed for the NMA. Search strategies were designed to identify any studies on cabozantinib and possible comparators. Based on the results of this broad systematic literature search, inclusion and exclusion criteria were then applied for the selection of studies to inform the NMA. The PICOS framework guiding the development of the search strategy is shown in Table 1 and further search parameter restrictions are shown on Table 2. The details of databases searched are shown on Table 3. The search protocol is presented in full in S2 File search protocol.

**Table 1:** Eligibility criteria of the systematic literature review.

Category	Details
Population	Patients with renal cell cancer (advanced / metastatic, previously treated)
Intervention	Cabozantinib
Comparators	Everolimus, axitinib, nivolumab, sorafenib, sunitinib, lenvatinib
Outcomes	PFS OS Response rates Drug discontinuation Any other efficacy outcomes Safety outcomes Quality of life and other Patient-reported Outcomes Biomarkers for efficacy and safety
Study Design	RCT

**Table 2:** Further parameter restrictions.

Category	Details
Timeframe of Search	No time restriction
Language	No language restriction

**Table 3:** Bibliographic databases searched.

Databases	Date of Search
Medline (includes Medline in Process and other non-indexed citations with status: publisher, in-data review or Pubmed-not-Medline)	Jun 03, 2016
Embase	Jun 03, 2016
Cochrane Library (includes Cochrane Central Register of Controlled Trials, Cochrane Reviews, DARE, HTA Database, NHSEED)	Jun 03, 2016

Each of the records identified during the search was assessed for relevance against predefined inclusion and exclusion criteria (Table 4). Copies of potentially relevant full papers were obtained and further selection was undertaken based on full text review. Double independent record selection was undertaken during screening of titles/abstract as well as full texts, and discrepancies were resolved after discussion between reviewers or by a third reviewer.

**Table 4:** Inclusion and exclusion criteria.

Clinical effectiveness	Inclusion Criteria	Exclusion Criteria
Population	Patients with previously treated advanced or metastatic renal cell carcinoma	Patients <18 years of age
		Healthy subjects
		Animal studies
Intervention	The following interventions in the second- (and further-) line setting: • Cabozantinib (Cabometyx) • Axitinib (Inlyta) • Everolimus (Afinitor) • Sorafenib (Nexavar) • Sunitinib (Sutent) • Lenvatinib (Kisplyx) • Nivolumab (Opdivo) Note: Combination therapies also possible	Interventions in the first-line setting
Comparators	Any, including placebo and best supportive care (BSC)	Radiotherapy, surgery and other non-pharmaceutical treatments
Outcomes	• Overall survival • Progression free survival	Patient-reported outcomes Biomarker results Safety results
Trial Design	Randomised controlled trial (RCT) Systematic reviews, meta-analyses, HTA for screening of bibliographies only	Non-RCT Comments, letters, editorials Non-systematic reviews
Timeframe	All publication years	
Language restrictions	• English • French • German • Italian • Spanish	Publications with abstract in English but full text in language other than listed in inclusion criteria will not be included but listed.

To identify relevant evidence, a clear definition of the study participants, interventions, comparison groups, outcomes and study types of interest was required. In order to ensure that the included studies were sufficiently homogeneous to form part of a NMA, only prospective comparative randomised controlled trials (RCTs) were included. Retrospective studies were excluded from the review and NMA.

### Trial selection for the network meta-analysis

For the NMA the following comparators were included: axitinib, everolimus, nivolumab, sorafenib, sunitinib and BSC/placebo. Studies with treatment which were not relevant for this NMA were excluded unless they provide an intermediate link between cabozantinib, axitinib, everolimus, nivolumab, sorafenib, sunitinib and BSC/placebo.

### Statistical analysis

Patient level data for the METEOR study were provided by the study sponsor. For other studies in the evidence network, the method published by Guyot et al. [19] was used to estimate the number of deaths and the number of patients censored every month from the published Kaplan-Meier curves [14;20–22]. We carried out statistical analyses as well as used visual inspection to assess whether the proportional hazards assumption is violated. We used tests and graphs based on the Schoenfeld residuals and Therneau and Grambsch test.

The Bayesian NMA was implemented with the following five parametric survival functions: log-normal, log-logistic, Weibull, Gompertz and exponential distributions, on the extracted PFS and OS data, as described by Ouwens et al. 2010 [18]. The Bayesian approach was chosen because it facilitates estimations on pooled data. A posterior probability distribution of this pooled relative effect was obtained [18;23]. Fixed-effects models were considered for this analysis and additional random-effects models were compared with fixed-effects models with purpose of heterogeneity and inconsistency checking. Fixed-effects model and random-effects model are defined in S3 File algorithms for fixed effect model and S4 File algorithms for random effect model. NMA comparing HRs was also carried out. We compared the logarithms of HRs, as described by Caldwell et al.[24]. Analysis was executed in R package *netmeta* using fixed effect model.

#### Bayesian estimation of survival parameters

Four of the models assumed two-parameter distributions (log-normal, log-logistic, Weibull, Gompertz). One model assumed one-parameter exponential distributions i.e. providing a fixed HR and hence assuming time-independent hazard ratios. Model parameters were estimated using a Markov Chain Monte Carlo (MCMC) method on WinBUGs [23]. The WinBUGs sampler was run for 50,000 iterations with the first 25,000 iterations discarded as “burn-in”. Convergence of the chains was checked using the Gelman-Rubin statistic [25]. Further details on the programming of the parametric survival curves are provided in S5 File Programming code for NMA.

Under the verification of the transitivity for each applied distribution (S6 File transitivity property) and the absence of heterogeneity or inconsistency in the network (S7 File Heterogeneity & inconsistency), Bayesian meta-analysis models were used to determine the difference of treatment effects. Goodness of model fit is specified in S8 File Goodness of fit.

## Results

### Literature selection

**Fig 1:**
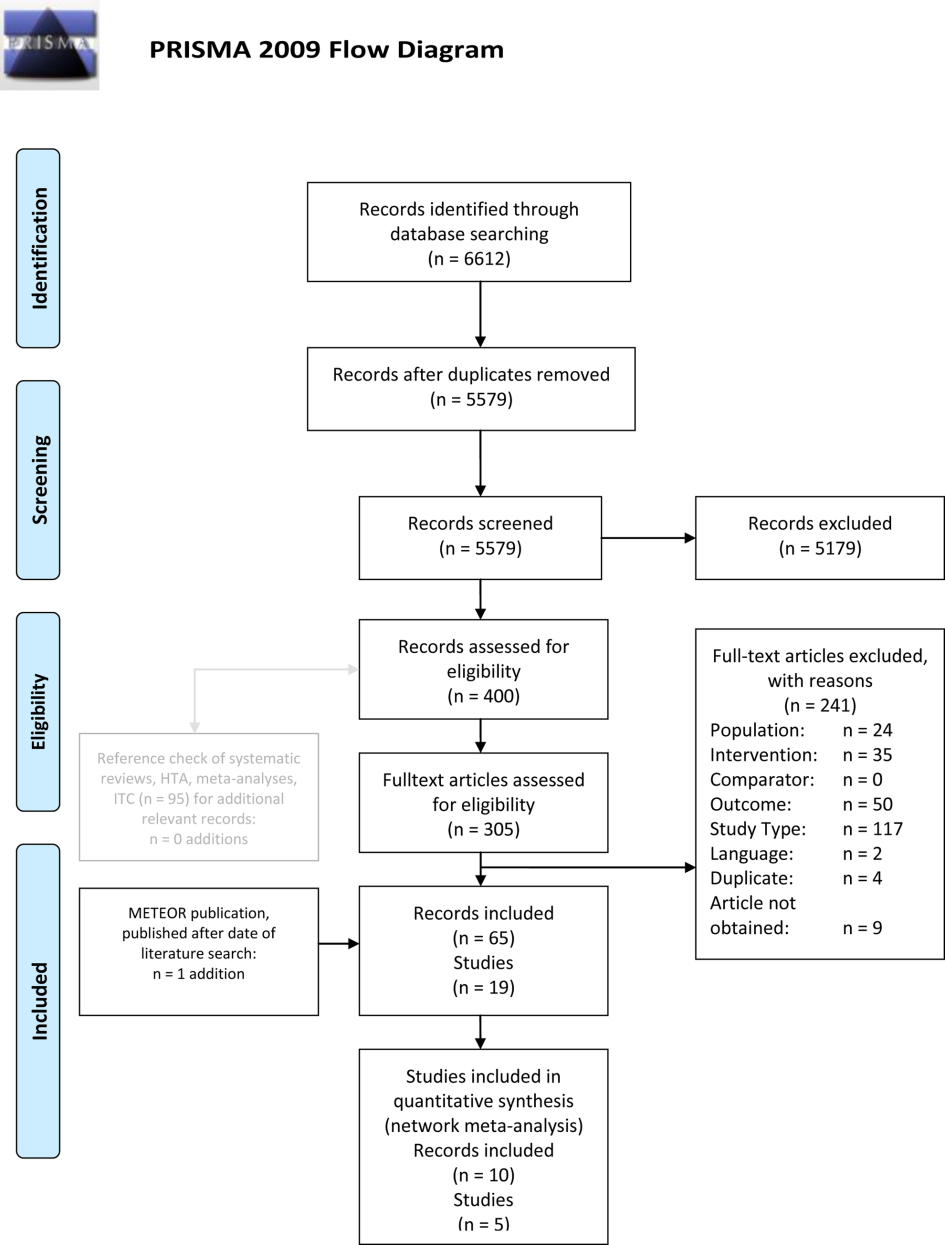
PRISMA chart.

The systematic literature search for RCTs on cabozantinib and 6 of its comparators retrieved 6,612 citations. After excluding duplicates (n = 1,033) and screening against inclusion/exclusion criteria 5,182 titles/abstracts were excluded. 400 citations were found eligible for the screening on full-text level. 95 of these 400 records were systematic reviews, meta-analyses or health technology assessment (HTA). Reference lists of these publications were checked for any further relevant studies. This process did not yield any additions. Of the 305 full-text articles 241 publications were excluded. Due to language restrictions two records were excluded: both records were in Chinese with an English abstract. The abstracts indicated that both are systematic reviews with meta-analysis. In total, 65 publications, referring to 19 different studies, were considered for potential inclusion into the NMA, as shown in the PRISMA chart in Fig 1. 64 of the studies were identified through the systematic literature search. One additional paper on the METEOR study was published after the date of the literature search, as shown in the PRISMA chart (Fig 1), and included for further analysis.

To perform a NMA, the studies must form a connected network. Network diagrams showing which of the treatments and comparator treatments are linked for each outcome were developed. In total 19 studies were identified for potential inclusion into the NMA. Multiple publications reporting the same study were identified and grouped as associated references. The primary RCT data sources identified in the systematic literature search are summarised in Table 5. Of the identified studies, ten studies were excluded because these were comparisons of everolimus or sorafenib to agents out of scope of this study: bevacizumab+sorafenib, GDC-0980, MK2206, AZD2014, apitolisib, temsirolimus, dovitinib, BNC105P+everolimus, tivozanib, lenvatinib, and lenvatinib + everolimus. These studies were excluded because they neither contained comparison to a treatment of interest (cabozantinib, everolimus, nivolumab, axitinib, sorafenib or best supportive care), nor did they provide a link between comparators that would not otherwise have a common comparator. The studies that have been excluded for this reason are: NCT01442090, NCT01239342, ZEBRA, DusrupTOR-1, ROVER, NCT02330983, TIVO-1, GOLD, INTORSECT, NCT01136733 [26–35]. Further four studies were excluded from the network meta-analysis: RECORD-3 [36], SWITCH [37], ESPN [38], and study by Ratain et al. 2006 [39]. The main reason for exclusion was sequential study design. Table 6 below gives details of the further exclusions.

**Table 5:** Primary RCT data sources included in the network evidence base.

Trial name	Treatment arms	Primary data source
METEOR	Cabozantinib vs everolimus	Choueiri et al. 2016 [15]
RECORD-1	Everolimus/BSC vs placebo/BSC	Motzer et al. 2010 [20]
CheckMate025	Nivolumab vs everolimus	Motzer et al. 2015 [14]
TARGET	Placebo vs sorafenib	Escudier et al. 2009 [21]
AXIS	Axitinib vs sorafenib	Rini et al. 2011 [22]
NCT01136733	Everolimus vs lenvatinib vs lenvatainib + everolimus	Motzer et al. 2015 [35]
RECORD-3	Sorafenib vs sunitinib	Motzer et al. 2014 [36]
SWITCH	Sorafenib vs sunitinib	Eichelberg et al. 2014 [37]
TIVO-1	Tivozanib vs sorafenib	Motzer et al. 2013 [29]
DisrupTOR-1	Everolimus vs BNC105P+everolimus	Pal et al. 2015 [31]
ESPN	Everolimus vs sunitinib	Tannir et al. 2014 [38]
GOLD	Dovitinib vs sorafenib	Motzer et al. 2014 [30]
INTORSECT	Temsirolimus vs sorafenib	Hutson et al. 2014 [27]
ROVER	Apitolisib vs everolimus	Powles et al. 2016 [33]
ZEBRA	AZD2014 vs Everolimus	Powles et al. 2016 [34]
NCT01239342	MK2206 vs everolimus	Jonasch et al. 2013 [28]
NCT01442090	GDC-0980 vs everolimus	Powles et al. 2014 [32]
NCT02330783	Bevacizumab+sorafenib vs sorafenib	Guo et al. 2015 [26]
Ratain 2006	Sorafenib followed by sorafenib vs placebo	Ratain et al. 2006 [39]

**Key**: BSC, best supportive care; vs, versus

**Table 6:** Key methodological and clinical reasons for further exclusions.

	Key methodological and clinical parameters supporting exclusion
RECORD-3 [36]	• Sequential design and hence randomisation only for first-line treatment • No PFS or OS data available for second line only
SWITCH [37]	• Sequential design and hence randomisation only for first-line treatment • Second line base line characteristics not reported • No OS data for second line
ESPN [38]	• Only non-clear cell patients included • No blinding details available
Ratain 2006 [39]	• No information on prior VEGFR therapies

The NMA was planned on the endpoints PFS and OS. These are commonly selected as the primary and secondary efficacy endpoints in oncology trials, including in trials with aRCC population. Data were extracted by one person from the reports on a pre-defined extraction template in excel. Data were sought and extracted for PFS and OS (mean median, associated hazard ratios, and Kaplan-Meier data, plus all associated confidence intervals). Data availability for OS and PFS hazard ratios and KM curves was assessed in all included trials.

Intent-to-treat (ITT) and cross-over results (in those trials where cross-over was present) were identified for the OS endpoint. PFS can be measured by an independent review committee (IRC) and investigators (INV). IRC assessment of disease progression was deemed likely to lead to the least biases, and hence it was prioritised if available. INV-assessed PFS was considered only in cases where IRC-assessed PFS was not available. In three of the included studies (RECORD-1, TARGET and AXIS) PFS was measured in the interim analysis by the IRC, and no further IRC-assessed updates were reported in subsequent publications. Further publications reported final OS results, and in some studies PFS continued to be assessed by INV. In these cases interim results for IRC-assessed PFS were used. In CheckMate025 study (nivolumab versus everolimus) no IRC-assessed PFS could be identified. In the nivolumab NICE single technology appraisal the manufacturer stated that disease assessment was not conducted independently in CheckMate025. The stated reason was that the CheckMate025 trial was designed with OS as primary endpoint, and independent review of secondary endpoints was not deemed necessary, as per regulatory guidelines [40].

**Fig 2:**
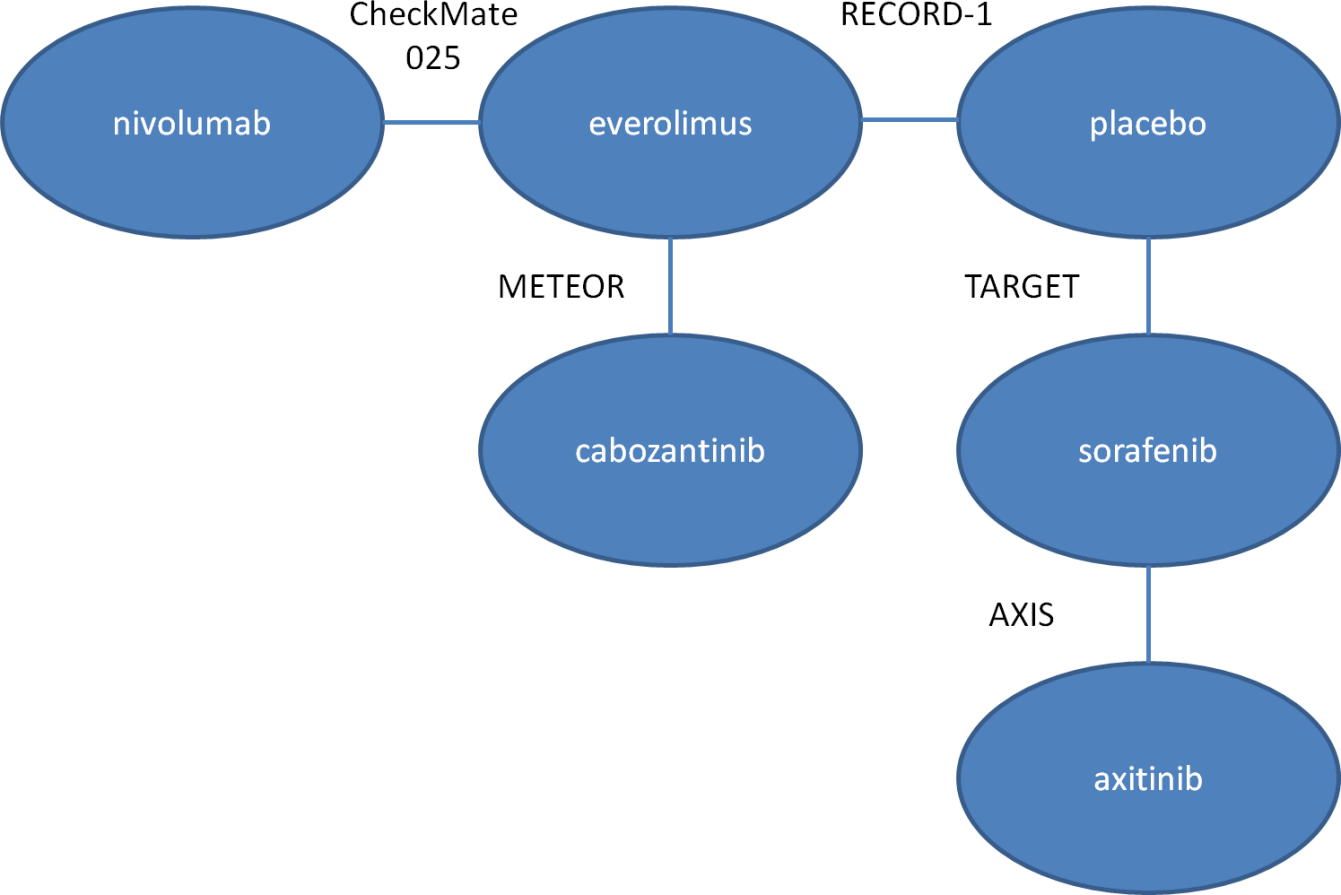
Evidence network for meta-analysis (OS, PFS).

A key consideration for any NMA is whether the studies that have been identified are suitably homogeneous to facilitate reliable comparison. This similarity comparison is achieved by comparing selected data from candidate studies; covariates that act as relative treatment effect modifiers must be similar across trials [41]. The similarity of the studies in each network was assessed (Table 7). The availability of subgroup results for PFS and OS endpoints was also assessed. The final network utilised in the NMA is presented in Fig 2. The network for OS and PFS endpoints are the same.

**Table 7:** Assessment of similarity between identified studies and availability of outcomes and subgroup results.

	Study type	Prior therapies	Prognostic score (MSKCC)	Subgroup results available by
METEOR [15]	RCT: Yes Phase: III Double blinded: Open-label Design: parallel	1 prior VEGFR Cabozantinib: 71% Everolimus: 70% 2+ prior VEGFR Cabozantinib: 29% Everolimus: 30%	Favourable: 43–44% Intermediate: 40–43% Poor: 14–16% Missing: 0%	Patient level data available
RECORD-1 [20]	RCT: Yes Phase: III Double blinded: Yes Design: cross-over	1 prior VEGFR Everolimus: 74% Placebo: 74% 2+ prior VEGFR Everolimus: 26% Placebo: 26%	Favourable: 28–29% Intermediate: 56–57% Poor: 14–15% Missing: 0%	Prognostic score: Yes Type of prior therapies: Yes Number of prior therapies: No Cross-over adjusted: Yes
CheckMate025 [14]	RCT: Yes Phase: III Double blinded: Open-label Design: parallel	1 prior VEGFR [17] Nivolumab: 72% Everolimus: 72% 2 prior VEGFR Nivolumab: 28% Everolimus: 28%	Favourable: 35–36% Intermediate: 49% Poor: 15–16% Missing: 0%	Prognostic score: Yes Type of prior therapies: No Number of prior therapies: Yes Cross-over adjusted: NA
TARGET [21]	RCT: Yes Phase: III Double blinded: Yes Design: cross-over	No prior VEGFR therapy was received among patients.	Favourable: 45–53%[42] Intermediate: 47–55%[42] Poor: NR Missing: NR	Prognostic score: No Type of prior therapies: No Number of prior therapies: No Cross-over adjusted: Yes
AXIS [22]	RCT: Yes Phase: III Double blinded: Yes Design: parallel	1 prior treatment	Favourable: 28% Intermediate: 36–37% Poor: 33% Missing: 2–3%	Prognostic score: No Type of prior therapies: Yes Number of prior therapies: No Cross-over adjusted: NA

**Key**: BSC, best supportive care; RCT, randomised controlled trial; PFS, progression free survival; IRC, independent review committee assessed; INV; investigator assessed; vs, versus; MSKCC, Memorial Sloan-Kettering Cancer Center; NA, not applicable; NR, not reported.

There were differences between the included trials, as shown in Table 7. The main sources of difference were presence/absence of a cross-over trial design (RECORD-1, TARGET), the number and type of prior therapies as well as baseline prognostic scores (e.g. Memorial Sloan-Kettering Cancer Center [MSKCC] score).

Cross-over is present in RECORD-1 and TARGET studies. Hence, cross-over has an impact on cabozantinib vs axitinib and BSC comparisons. However, comparison to nivolumab is not impacted by the cross-over issue. In the RECORD-1 trial the estimate for OS HR for everolimus vs placebo (BSC) is 0.87 [0.65, 1.17] in ITT population and 0.60 [0.22, 1.65] once adjusted to cross-over by using rank-preserving structural failure time (RPSFT) model published by Korhonen et al. [43]. The RPSFT model relies on assumption of constant effect of active treatment (in this case everolimus) in terms of relative survival time. Hence, the effect does not depend on when active treatment was initiated. Since the method requires additional censoring of patient data, the precision of the HR estimate is lower than for the ITT estimate. However, the method was shown to be preferable to simple adjustments, such as censoring of patients at time of crossover [43]. It should be noted that one other possible approach has been considered by Hollaender, using inverse probability of censoring weights and multivariate Cox models [44]. Results in best multivariate model (ranked according to Akaike information criterion) were HR = 0.47 [0.27, 0.82]. It relies on a strong (and un-testable) assumption of no unmeasured confounders, therefore RPSFT model was preferred, but the implications of using Cox model adjustment are also discussed below. In the TARGET study, an analysis with censoring of placebo-assigned patients who crossed over to sorafenib at the start of cross-over was conducted in addition to the ITT analysis [21]. The adjustment methodology is simple censoring of all cross-over patients.

**Fig 3:**
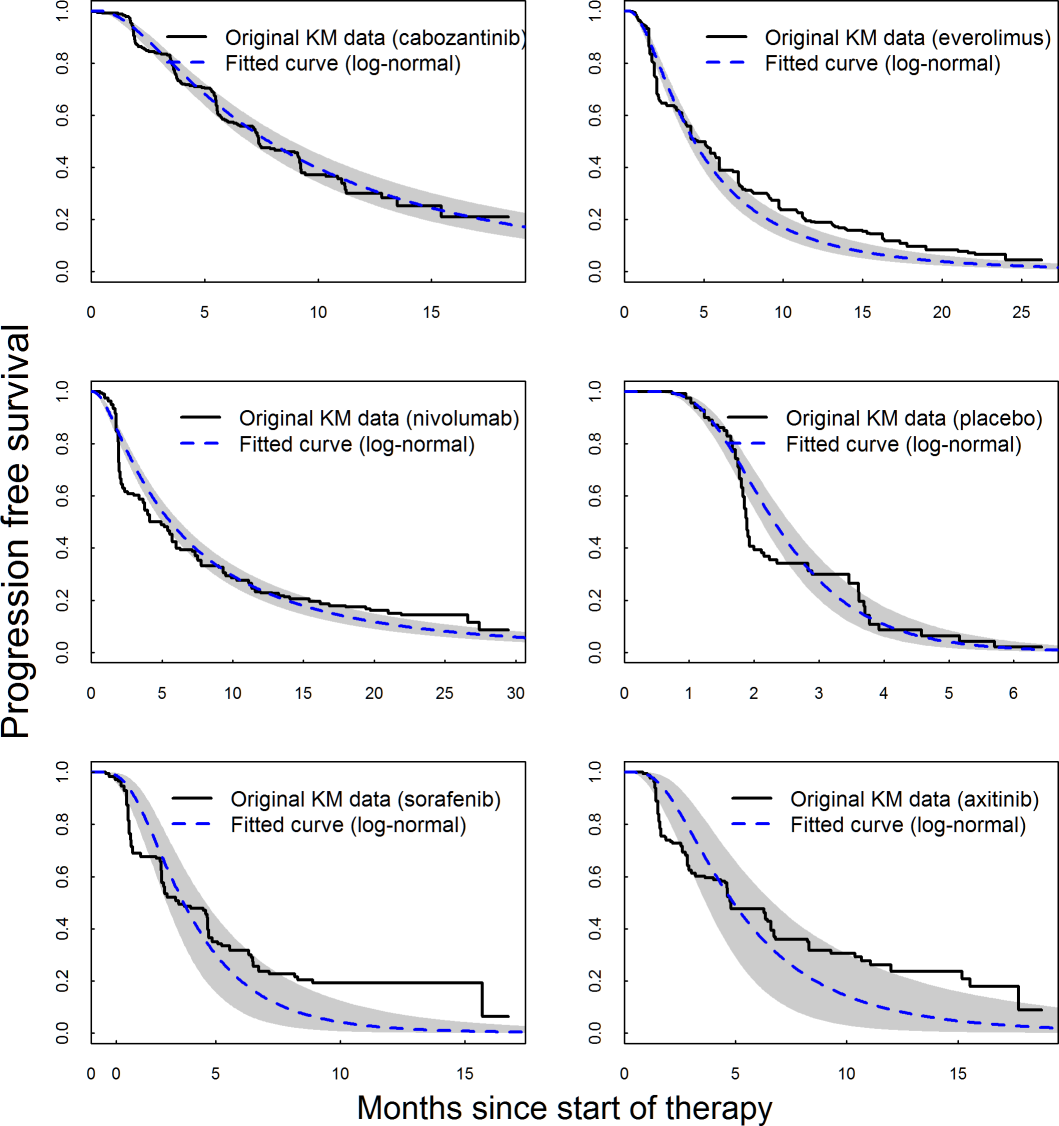
Fitted PFS based on the best fitting Bayesian fixed-effects model (log-normal) overlaid on extracted Kaplan-Meier (KM) data, with shaded areas representing 95% credible intervals.

**Fig 4:**
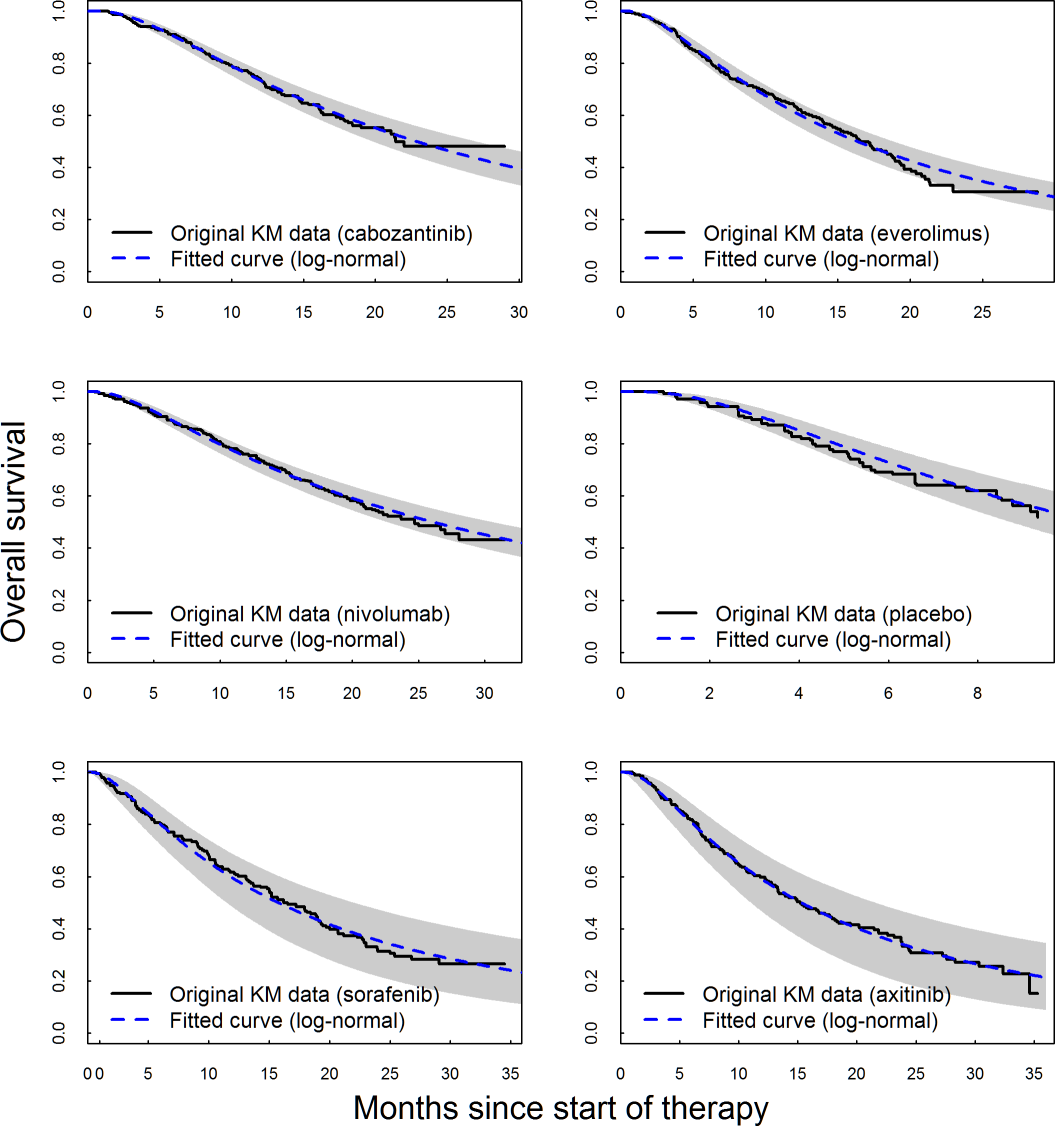
Fitted OS based on the best fitting Bayesian fixed-effects model (log-normal) overlaid on extracted Kaplan-Meier data, with shaded areas representing 95% credible intervals.

**Fig 5:**
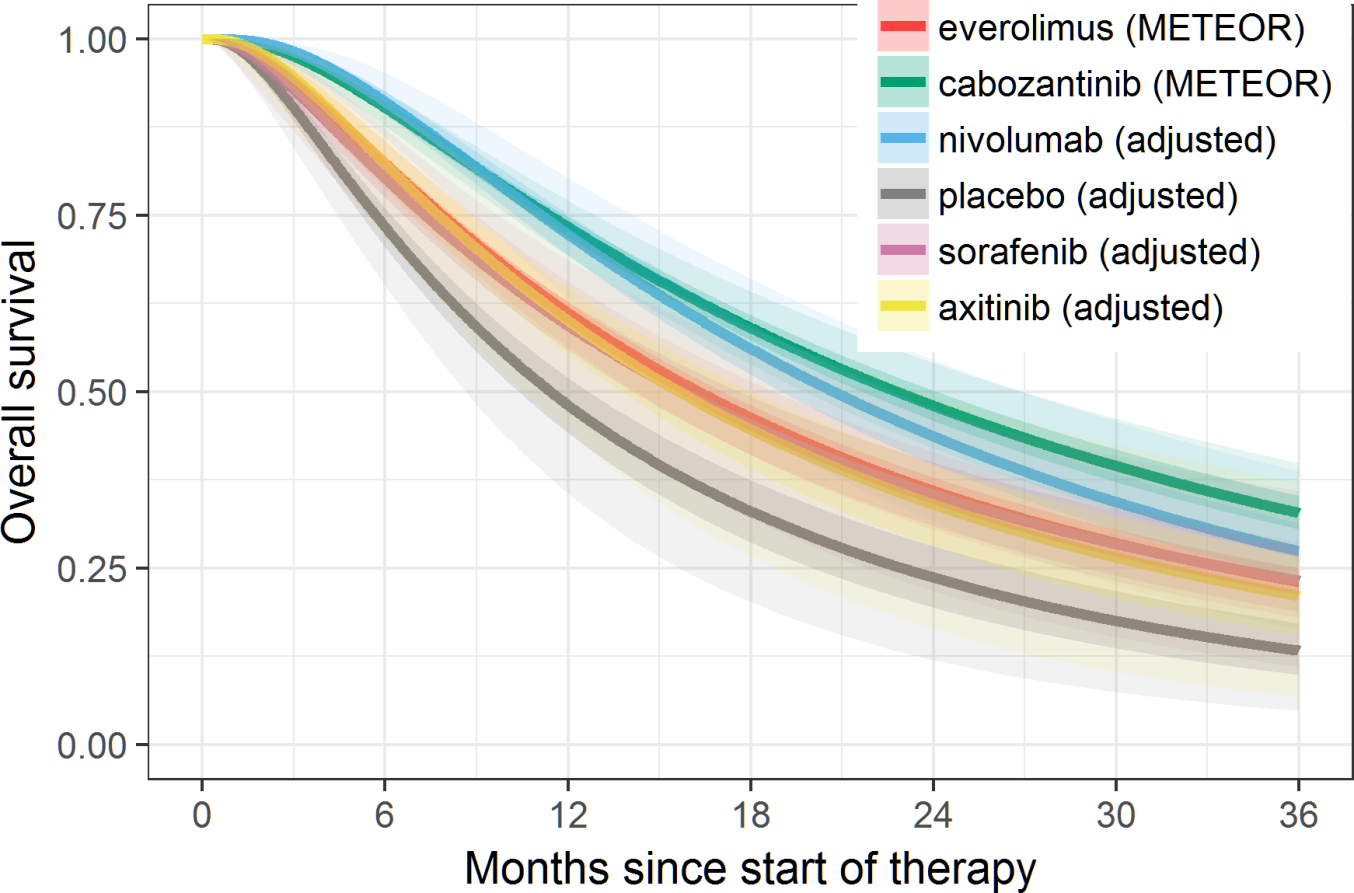
Averaged OS curves over time derived from the log-normal fixed-effects model, adjusted to the baseline from METEOR study, with shaded areas representing 95% credible intervals.

Trials included in network of evidence for the analysis were different in number of allowed prior therapies and the distribution of compounds in patient cohorts. In METEOR study more than one prior therapy was allowed. Patients were included in the study if they had received at least one previous VEGFR TKI (there was no limit to the number of previous treatments). In CheckMate025 patients were eligible to participate if they had received one or two previous regimens of antiangiogenic therapy. In RECORD-1, previous therapy with sorafenib, sunitinib or both was allowed. TARGET study included patients if they had progressed after one systemic treatment within the previous 8 months. AXIS study patients had previously received one previous systemic first line regimen with a sunitinib-based, bevacizumab plus interferon-alfa-based, temsirolimus-based, or cytokine-based regimen, which reflected regimens with regulatory approvals at the time of study design. In our study we only included the prior-sunitinib sub-population, because it was considered more comparable to the baseline study population of the METEOR study. Table 7 summarises the baseline prior therapies for each study and shows the availability of results for subgroups of patients by prior therapy. For CheckMate025 results stratified by number of prior therapies received were identified in nivolumab NICE single technology appraisal (Appendix A8, Figs 2–5 on page 301–304) [40]. Results were not identified by type of prior therapies. For RECORD-1 stratified estimates were available for PFS, but not OS. In the TARGET study publication no subgroup data were identified that stratified results by number/type of prior therapies. AXIS study reported results by type of first line therapy. Due to lack of consistency and availability of results across all trials in the network, it is not possible to analyse results by prior therapy. In the METEOR study results were consistent regardless of number of prior therapies (see Table 8). Available results for CheckMate025, RECORD-1, and AXIS studies are reported in Table 9, Table 10 and Table 11, respectively. These tables illustrate the differences in reported information between the trials.

**Table 8:** Subgroup results–number of previous therapies received (METEOR).

Number of VEGFR TKIs	PFS Choueiri et al. 2016 [15]		OS Choueiri et al. 2016 [15]	
	HR	95% CI	HR	95% CI
1	0.52	0.41–0.66	0.65	0.50–0.85
≥2	0.51	0.35–0.74	0.73	0.48–1.10

**Key:** CI, confidence interval; HR, hazard ratio; OS, overall survival; PFS, progression-free survival; TKI, tyrosine kinase inhibitor; VEGFR, vascular endothelial growth factor

**Table 9:** Subgroup results–number of previous therapies received (CheckMate025).

Number of VEGFR TKIs	PFS		OS Motzer et al. 2015 [14]	
	HR	95% CI	HR	95% CI
1	-	-	0.71	0.56–0.90
2	-	-	0.89	0.61–1.29

**Key:** CI, confidence interval; HR, hazard ratio; OS, overall survival; PFS, progression-free survival; TKI, tyrosine kinase inhibitor; VEGFR, vascular endothelial growth factor

**Table 10:** Subgroup results–number of previous therapies received (RECORD-1).

Number of VEGFR TKIs	PFS Motzer et al. 2008[45]		OS	
	HR	95% CI	HR	95% CI
Sorafenib only	0.29	-	-	-
Sunitinib only	0.30	-	-	-
Both	0.28	-	-	-

**Key:** CI, confidence interval; HR, hazard ratio; OS, overall survival; PFS, progression-free survival; TKI, tyrosine kinase inhibitor; VEGFR, vascular endothelial growth factor

**Table 11:** Subgroup results–number of previous therapies received (AXIS).

Number of VEGFR TKIs	PFS Rini et al. 2011 [22]		OS Motzer et al. 2013[46]	
	HR	95% CI	HR	95% CI
Sunitinib-containing regimen	0.741	0.574–0.958	0.997	0.782–1.270
Bevacizumab-containing regimen	1.147	0.573–2.295	Not reported	Not reported
Temsirolimus-containing regimen	0.595	0.188–1.886	Not reported	Not reported
Cytokine-containing regimen	0.462	0.318–0.673	0.813	0.555–1.191

**Key:** CI, confidence interval; HR, hazard ratio; OS, overall survival; PFS, progression-free survival; TKI, tyrosine kinase inhibitor; VEGFR, vascular endothelial growth factor

Evidence suggests that the number of prior therapies does not affect the relative efficacy of cabozantinib vs everolimus. For OS, nivolumab vs everolimus in CheckMate025 shows consistent results for patients with 1 and 2 prior therapies, although results were not statistically significant in the subgroup who received 2 prior treatments.

The MSKCC prognosis score was commonly used to stratify PFS and OS estimates: PFS in METEOR, RECORD-1 and AXIS and OS in METEOR and CheckMate025. TARGET trial did not include any patient with poor MSKCC prognosis and no subgroup analysis was presented by MSKCC prognosis. No subgroup result was identified for initial prognosis for AXIS study. An overview of identified HRs by prognosis is shown on Table 12. The identified data does not allow for recreating NMA for particular prognosis (poor/intermediate/favourable) neither based on HRs nor KM plots, but qualitative assessment is possible.

**Table 12:** Subgroup results–availability of HR results by prognostic score.

End-point	Study	Comparator	Baseline	HR for poor prognosis [95% CI]	HR for intermediate prognosis [95% CI]	HR for favourable prognosis [95% CI]
OS	CheckMate025	Nivolumab	Everolimus	0.47 [0.30, 0.73]	0.76 [0.58, 0.99]	0.89 [0.59, 1.32]
OS	METEOR	Cabozantinib	Everolimus	0.65 [0.39, 1.07]	0.67 [0.48, 0.94]	0.66 [0.46, 0.96]
PFS	AXIS	Axitinib	Sorafenib	0.68 [0.49, 0.94]	0.80 [0.58, 1.10]	0.50 [0.33, 0.76]
PFS	RECORD-1	Everolimus	Placebo	0.44 [0.22, 0.85]	0.32 [0.22, 0.44]	0.31 [0.19, 0.50]
PFS	METEOR	Cabozantinib	Everolimus	0.70 [0.42, 1.16]	0.47 [0.35, 0.62]	0.51 [0.38, 0.69]

**Key**: OS, overall survival; PFS, progression free survival, CI, confidence interval

In the METEOR study, OS results for subgroups of patients with intermediate/favourable prognosis, in comparison with the overall population, suggest potential larger efficacy of cabozantinib compared to everolimus. Subgroup results for patients with poor prognosis in CheckMate025 study, in comparison to the overall study population, suggest larger efficacy of nivolumab compared to everolimus in this subpopulation. For PFS it can be noted that for subgroups of patients with intermediate/favourable prognosis, in comparison with the overall population, results suggest potential larger efficacy of cabozantinib compared to everolimus and of everolimus compared to placebo. As patients with poor prognosis were not included in the TARGET study, excluding such patients from analysis might lead to PFS HR of cabozantinib vs axitinib more favourable to cabozantinib, but data (TARGET trial intermediate/favourable subgroups) is missing to conduct such comparison quantitatively.

Risk of bias was assessed with an adapted checklist for RCTs as proposed by the Centre for Reviews and Dissemination. Criteria for quality assessment included adequacy of randomization method, allocation concealment, homogeneity of baseline characteristics between treatment groups and blinding. The study quality assessment was conducted by two independent assessors. The quality assessment of included trials showed that demographic and baseline characteristics were balanced between the treatment arms in all included studies. None of the studies reported unexpected dropouts between study groups. All 5 studies reported intent-to-treat (ITT) analysis and reported appropriate method to account for missing data. A potential risk of bias arises from investigators, participants and outcome assessors not being blind to treatment allocation in all studies. Effective blinding can ensure that the compared groups receive a similar amount of attention, ancillary treatment and diagnostic investigations. Blinding is not always possible, however, and three of the studies were not double blinded:

- METEOR: Patients and investigators were not blinded to study treatment. A masked independent radiology committee assessed progression-free survival, overall survival, tumour response, duration of response, and changes on bone scans.
- CheckMate025: This was an open-label study. Patients and investigators were not blinded to study treatment.
- AXIS: This was an open-label study. Patients and investigators were not masked to study treatment. Progression-free survival and objective response rate were assessed by a masked independent radiology review.

CheckMate025 was an open-label study with no IRC assessment of end-points such as progression-free survival. The lack of blinding of the INV may increase the risk that knowledge of which intervention was received, rather than the intervention itself, affects outcome measurement. The blinding of outcome assessors can be especially important for the assessment of subjectively assessed outcomes.

### Statistical analysis

The Schoenfeld residuals and Therneau and Grambsch tests indicate that the proportional hazards assumption holds for METEOR, RECORD-1, and AXIS studies. However, the assumption is violated in CheckMate025 and TARGET studied for both PFS and OS endpoints. Intent-to-treat (ITT) and cross-over results (in those trials where cross-over was present) were identified for the OS endpoint. PFS can be measured by an independent review committee (IRC) and investigators (INV). IRC assessment of disease progression was deemed likely to lead to the least biases, and hence it was prioritized if available. INV-assessed PFS was considered only in cases where IRC-assessed PFS was not available. In three of the included studies (RECORD-1, TARGET and AXIS) PFS was measured in the interim analysis by the IRC, and no further IRC-assessed updates were reported in subsequent publications. Further publications reported final OS results, and in some studies PFS continued to be assessed by INV. In these cases interim results for IRC-assessed PFS were used. In CheckMate025 study (nivolumab versus everolimus) no IRC-assessed PFS could be identified. Table 13 shows the details of data availability by endpoint.

**Table 13:** Sources of OS (ITT), OS (cross-over adjusted), PFS IRC-, and PFS INV-assessed KM plots and hazard ratio results.

HR (95% confidence interval)	OS ITT	OS Cross-over adjusted	PFS Independent review committee (IRC)	PFS Investigator assessed (INV)
**METEOR**	0.66 (0.53–0.83) Patient level data (published in Fig 2 [14])	Not applicable	0.51 (0.41–0.62) Patient level data (published in Fig 4 [14])	Not applicable
**RECORD-1**	0.87 (0.65–1.15) Fig 6A [20]	0.60 (0.22–1.65) Fig 5	0.30 (0.22–0.40) Fig 2 [45]	Not applicable, IRC PFS available
**CheckMate025**	0.73 (0.57–0.93) Fig 1 [14]	Not applicable	Not available	0.88 (0.75–1.03) Fig 2B [14]
**TARGET**	0.88 (0.74–1.04) Fig1A [21]	Fig 1B [21]	0.44 (0.35–0.55) Fig 2C [47]	Not applicable, IRC PFS available
AXIS**	0.997 (0.78–1.27) Fig 2B [46]	Not applicable	0.741 (0.573–0.958) Fig 2C [22]	Not applicable, IRC PFS available

**Key**: OS, overall survival; ITT, intention to treat; PFS, progression-free survival; HR, hazard ratio; CI, confidence interval; KM, Kaplan-Meier; INV, investigator assessed; IRC, independent review committee assessed.

Note

** prior-sunitinib group results used in the analyses.

**Sources**: [20] Motzer et al. 2010, [48] Korhonen et al. 2012, [45] Motzer et al. 2008, [14] Motzer et al. 2015, [21] Escudier et al. 2009, [47] Escudier et al. 2007, [46] Motzer et al. 2013, [22] Rini et al. 2011, [35] Motzer et al. 2015.

Despite the violation of PH assumption, we carried out the comparison of HRs. The results for OS and PFS are shown in Table 14 and Table 15, respectively.

**Table 14:** Network meta-analysis of OS HRs (cross-over adjusted, if applicable).

	HR (95% credible intervals)					
	Axitinib	Cabozantinib	Everolimus	Nivolumab	Placebo	Sorafenib
**Axitinib**	NA	1.96 (0.68, 5.7)	1.3 (0.46, 3.67)	1.78 (0.62, 5.09)	0.78 (0.57, 1.07)	1 (0.79, 1.26)
**Cabozantinib**	0.51 (0.18, 1.48)	NA	0.66 (0.53, 0.83)	0.9 (0.69, 1.19)	0.4 (0.14, 1.09)	0.51 (0.18, 1.43)
**Everolimus**	0.77 (0.27, 2.19)	1.52 (1.21, 1.9)	NA	1.37 (1.17, 1.61)	0.6 (0.22, 1.62)	0.77 (0.28, 2.12)
**Nivolumab**	0.56 (0.2, 1.62)	1.11 (0.84, 1.46)	0.73 (0.62, 0.86)	NA	0.44 (0.16, 1.2)	0.56 (0.2, 1.57)
**Placebo**	1.29 (0.93, 1.77)	2.53 (0.91, 6.98)	1.67 (0.62, 4.49)	2.28 (0.84, 6.23)	NA	1.28 (1.04, 1.59)
**Sorafenib**	1 (0.79, 1.27)	1.97 (0.7, 5.57)	1.3 (0.47, 3.58)	1.78 (0.64, 4.97)	0.78 (0.63, 0.97)	NA

**Table 15:** Network meta-analysis of PFS HRs (IRC-assessed when available, otherwise INV).

	HR (95% credible intervals)					
	Axitinib	Cabozantinib	Everolimus	Nivolumab	Placebo	Sorafenib
**Axitinib**	NA	2.13 (1.32, 3.43)	1.09 (0.7, 1.68)	1.24 (0.78, 1.96)	0.33 (0.23, 0.46)	0.74 (0.58, 0.95)
**Cabozantinib**	0.47 (0.29, 0.76)	NA	0.51 (0.42, 0.62)	0.58 (0.45, 0.74)	0.15 (0.11, 0.22)	0.35 (0.23, 0.52)
**Everolimus**	0.92 (0.59, 1.42)	1.96 (1.62, 2.37)	NA	1.14 (0.97, 1.33)	0.3 (0.23, 0.4)	0.68 (0.48, 0.97)
**Nivolumab**	0.81 (0.51, 1.29)	1.73 (1.35, 2.21)	0.88 (0.75, 1.03)	NA	0.26 (0.19, 0.36)	0.6 (0.41, 0.89)
**Placebo**	3.07 (2.2, 4.28)	6.54 (4.65, 9.19)	3.33 (2.51, 4.42)	3.79 (2.75, 5.22)	NA	2.27 (1.83, 2.83)
**Sorafenib**	1.35 (1.05, 1.74)	2.88 (1.92, 4.31)	1.47 (1.03, 2.1)	1.67 (1.13, 2.46)	0.44 (0.35, 0.55)	NA

We undertook a network meta-analysis comparing the relative efficacy of cabozantinib and its comparators. Fixed-effects model was preferred to random-effects models for this analysis, based on the preliminary evaluation of heterogeneity. The fixed-effects models provided as good model fitting as the random-effects models (Table 16 and Table 17), but were more robust against the choice of prior distributions. Moreover, the “burn-in” period was shorter than that with the random-effects models.

**Table 16:** Model fit statistics with PFS, fixed- and random-effects model.

Model fit statistics	Weibull		**Gompertz**		**Log-logistic**		**Log-normal**		**Exponential**	
	**FE**	**RE**	**FE**	**RE**	**FE**	**RE**	**FE**	**RE**	**FE**	**RE**
Residual deviance (Dbar)	6355.8	6355.3	6456.8	6456.3	6047.7	6047.9	5987.3	5987.0	6599.7	6600.1
Effective number of parameters (pD)	19.7	20.0	19.8	19.8	19.9	19.9	20.2	19.9	9.9	10.2
Deviance information criteria (DIC)	6375.5	6375.3	6476.6	6476.1	6067.6	6067.8	6007.5	6006.9	6609.6	6610.3

**Table 17:** Model fit statistics with OS, fixed- and random-effects model.

Model fit statistics	Weibull		Gompertz		Log-logistic		Log-normal		Exponential	
	FE	RE	FE	RE	FE	RE	FE	RE	FE	RE
Residual deviance (Dbar)	4364.9	4364.8	4443.8	4344.0	4314.5	4314.3	4293.8	4293.4	4535.8	4536.3
Effective number of parameters (pD)	20.0	19.7	19.6	20.1	20.0	19.9	20.2	19.8	9.8	10.2
Deviance information criteria (DIC)	4384.9	4384.5	4463.4	4464.1	4334.5	4334.2	4314.0	4313.2	4545.6	4546.5

**Fig 6:**
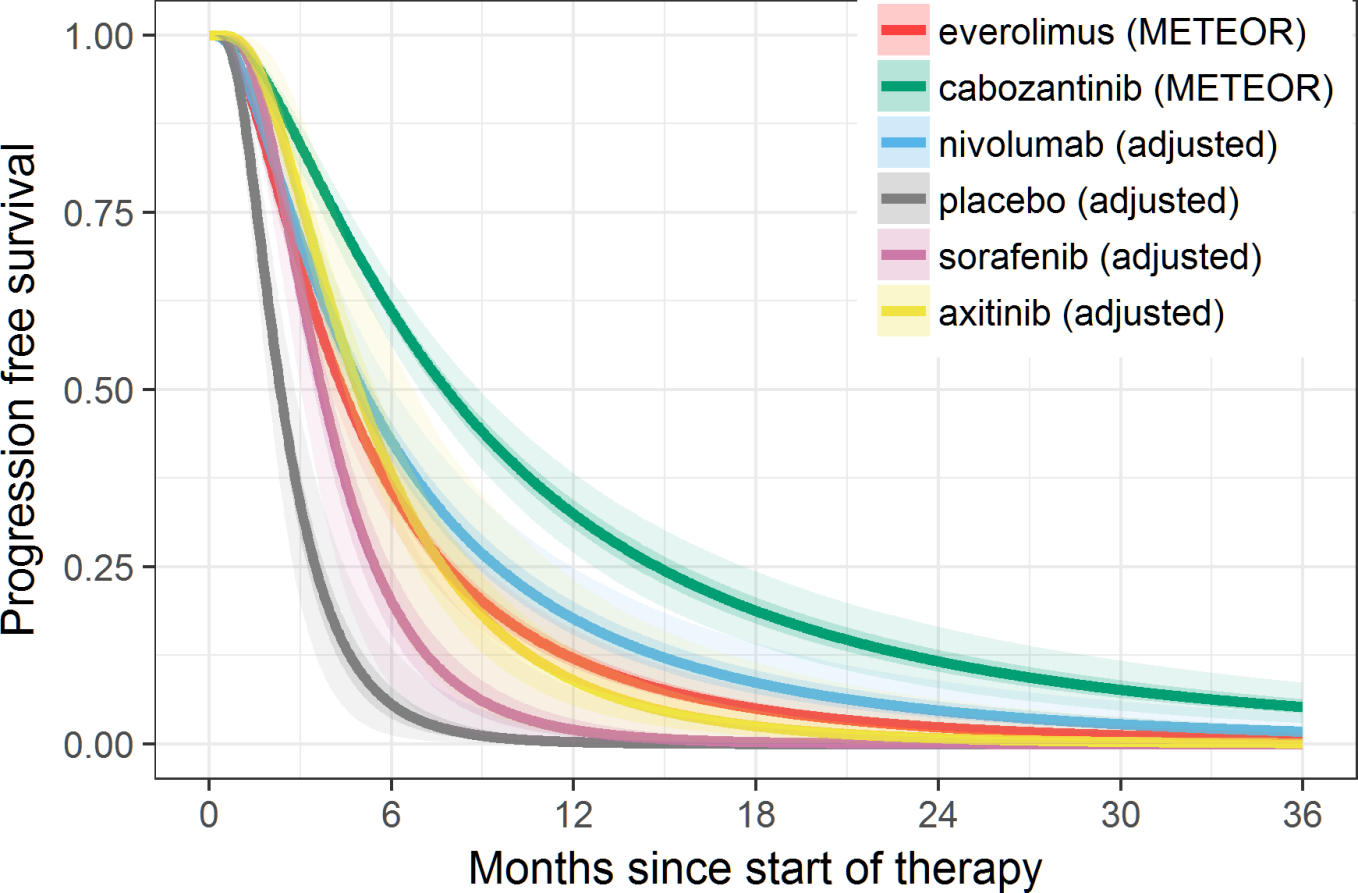
Averaged PFS over time derived from the Log-normal fixed-effects model, adjusted to the baseline from METEOR study, with shaded areas representing 95% credible intervals.

**Fig 7:**
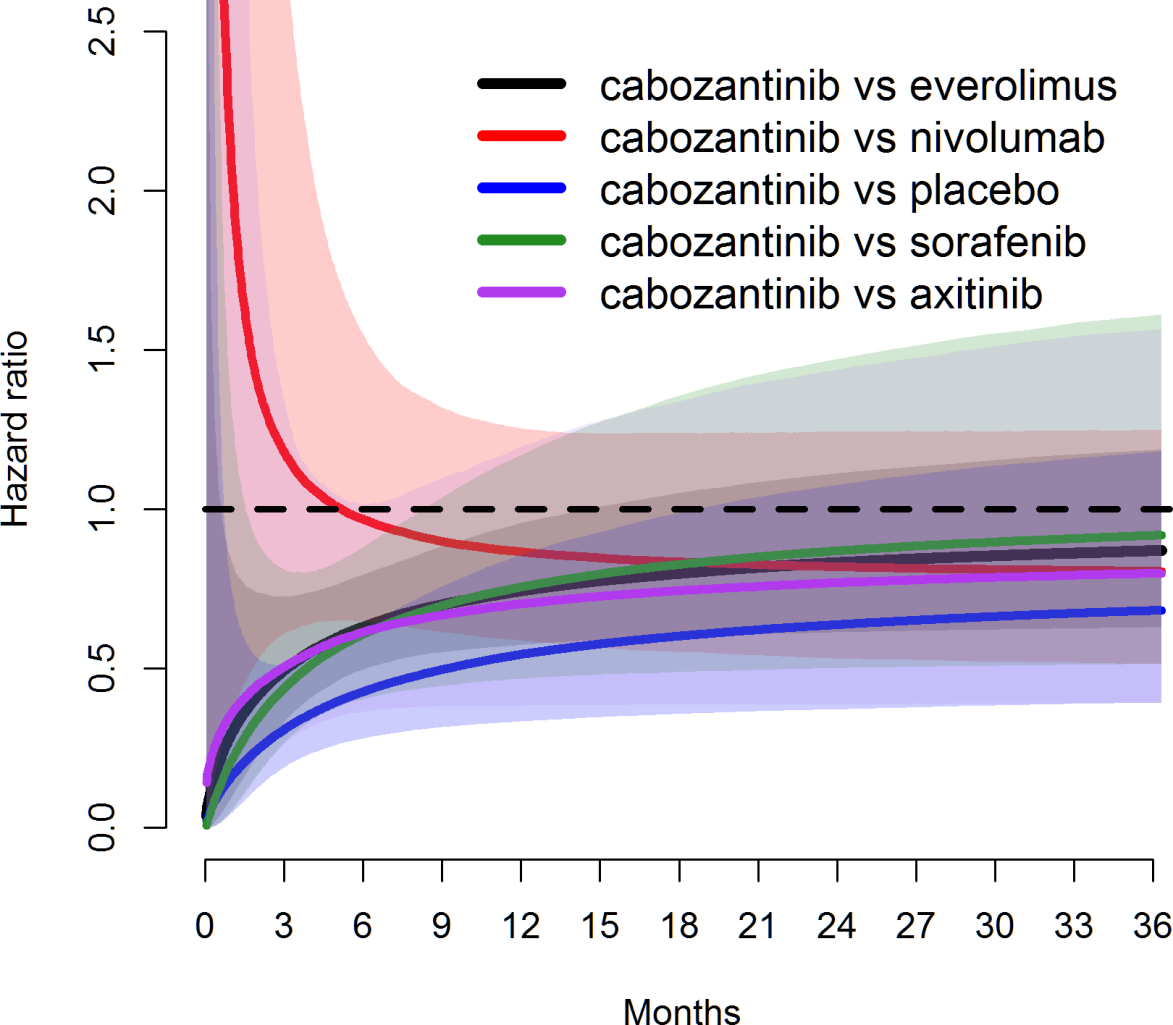
Estimated hazard ratios over time for cabozantinib vs other treatments for OS under log-normal distribution, with solid line representing median and shaded areas representing 95% credible intervals.

**Fig 8:**
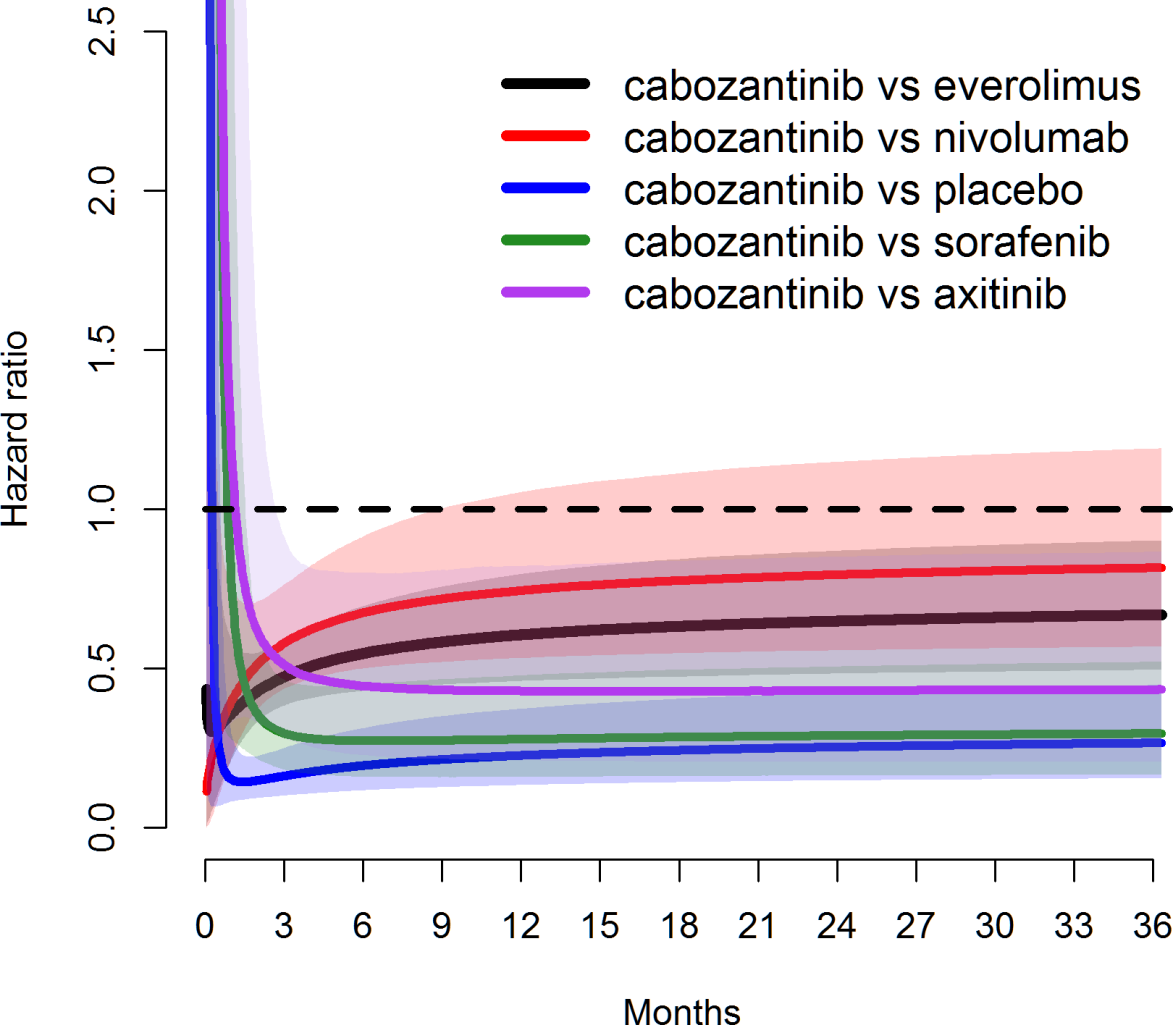
Estimated hazard ratios over time for cabozantinib vs other treatments for PFS under log-normal distribution, with solid line representing median and shaded areas representing 95% credible intervals.

Model fit statistics indicate that log-normal model provided the best statistical fit for both OS and PFS—see Tables 14 and 15, respectively. While log-normal model provided the best overall statistical fit for the whole network, the best statistical fit for each individual study varied. The fitted PFS and OS curves were superimposed on the extracted Kaplan-Meier data (Figs 3 and 4) to observe the visual fit of extracted data versus modelled data. Visually log-normal model provided a good fit for PFS and OS data across all treatments, with the exception of PFS for sorafenib and axitinib. For the best fitting model (log-normal), PFS and OS for patients under cabozantinib were predicted to be superior compared to all other treatments up to 36 months Figs 5 and 6). Other models (Weibull, Gompertz, log-logistic and exponential) showed similar results, exception for the PFS endpoint under the Gompertz model where nivolumab was preferred after 24 months (additional PFS and OS results are shown in S9 File Additional results for fixed effect model (full network)). The estimated hazard ratios for cabozantinib versus other treatments became more favorable to cabozantinib over time; after the first month for PFS and after the first four months for OS–see Figs 7 and 8.

## Discussion

Comparison of cabozantinib and its comparators is possible using both the NMA of constant HRs and comparison of parametric survival curves, when considering data availability. In our main analysis, we applied a Bayesian parametric NMA method to compare PFS and OS for cabozantinib and its comparators over time using five families of distributions. Although PH assumption was violated in the CheckMate025 and TARGET studies, we also carried out a comparison of HRs as an alternative method.

A recent NICE STA for nivolumab included a NMA comparing nivolumab to everolimus, axitinib, sorafenib and BSC [17]. This NMA was based on HRs, which we deemed an inappropriate approach due to violation of PHs assumptions. Regardless of the short-comings of the chosen method in the nivolumab NICE STA, the findings were in line with this NMA; nivolumab provided longer OS benefit compared to comparators other than cabozantinib. Wiecek & Karcher (2016) on the other hand applied a Bayesian parametric survival NMA method in order to compare OS for cabozantinib and nivolumab [16]. This analysis found that patients on cabozantinib exhibited a lower hazard of death over nivolumab until the fifth month of treatment, whereas patients on nivolumab exhibited a lower hazard of death after that time point. However, these analyses were based on immature OS data from the METEOR trial [13], and no superiority was found when the full data were incorporated [49].

The results of the HR NMA on the OS endpoint show a trend of improvement with cabozantinib therapy versus axitinib, everolimus, nivolumab and best supportive care. No statistically significant difference between cabozantinib and its comparators were shown, with the exception of comparison to everolimus. Cabozantinib differed significantly from its comparators with regard to PFS improvement. The parametric model that best fitted both the PFS and OS data was the log-normal distribution. In this model, the estimated PFS and OS probabilities for cabozantinib were 0.11 and 0.48 at 24 months. However, for its best comparator nivolumab, those estimated survival probabilities were 0.04 and 0.43 respectively. The model that provided the worst statistical fit to the data was found to be the exponential distribution, which assumed a constant HR over time as in the traditional approach. It, thus, provided further justification for our choice of model that did not require the PHs assumption. The results of the HR NMA are consistent with the results of the survival curve NMA; the PFS for patients receiving cabozantinib was predicted to be superior and the OS showed a trend towards improvement (OS) compared to axitinib, everolimus, nivolumab and best supportive care.

In the Bayesian analysis, the fixed-effects model has been chosen and the random-effects model has also been implemented for a sensitivity analysis, and heterogeneity & inconsistency checking. Even if it enables estimation of an additional between-study covariance matrix, the random-effects model returned quite similar comparison results to the fixed-effects model, which proved the homogeneity and consistency of the NMA at the network level. In the Bayesian framework, the fixed-effects model was favored because of its simplicity and robustness, where “robustness” meant the quickly-reached convergence of simulated Markov chain. However, if experts’ opinion were available, random-effects model would have an advantage by permitting the definition of a group of hyper-parameters which could reflect prior belief on the study heterogeneity. In the Bayesian framework, informative prior distributions may have improved the parameter estimation and reduced the uncertainty of estimation.

A limitation of the PFS analysis was the differing definitions of disease progression assessment (investigator versus independent). Independent review committee PFS was not available from CheckMate025 study, and hence investigator assessed PFS was used in this analysis instead. The results of this analysis were reported over 36 months, while the data were extracted from RCTs with shorter follow-up time. The OS in real-life may differ from the estimated OS, given that the treatment persistence could differ from the persistence observed in the RCTs. A possible direction for future work is to implement the generalized gamma distribution, which includes various commonly used parametric survival distributions, such as Weibull, exponential, log-normal distributions. An improved model fit over Weibull or log-normal would then be envisaged [50]. The NMA model extended the classical meta-analysis model by comparing multiple studies with multiple arms. Provided that the network would remain connected, i.e. neighbor studies shared a common treatment, new studies and treatments could be easily added to the model and the approach remains feasible.

## Conclusions

Our NMA reviewed and analyzed the existing literature for RCTs examining PFS and OS of cabozantinib, everolimus, axitinib, sorafenib, nivolumab and best supportive care treatments for aRCC in the second- and subsequent line settings. Our review has identified that cabozantinib significantly improves PFS outcomes in aRCC. The results of our NMA did not show a statistical difference between cabozantinib and comparator therapies with regards to OS, except when compared to everolimus. However, the results of the HR NMA and the parametric curve NMA favored cabozantinib.

## Supporting information

S1 File PRISMA checklist. (DOC)

S2 File Search protocol. (DOCX)

S3 File Algorithm for fixed effect model. (DOCX)

S4 File Algorithm for random effect model. (DOCX)

S5 File Programming code for NMA. (DOCX)

S6 File Transitivity property. (DOCX)

S7 File Heterogeneity & inconsistency. (DOCX)

S8 File Goodness of fit. (DOCX)

S9 File Additional results for fixed effect model (full network). (DOCX)

S10 File List of abbreviations. (DOCX)

S11 File Additional results for fixed effect model (narrow network). (DOCX)

S12 File Additional results for random effect model (full network). (DOCX)

S13 File Proportion of subsequent treatments received in different trials. (DOCX)

## References

[1] Ferlay J, Soerjomataram I, Dikshit R, Eser S, Mathers C, Rebelo M, et al Cancer incidence and mortality worldwide: sources, methods and major patterns in GLOBOCAN 2012. Int J Cancer 2015 3 1;136(5):E359–E386. doi: 10.1002/ijc.29210 25220842 10.1002/ijc.29210

[2] Ferlay J, Steliarova-Foucher E, Lortet-Tieulent J, Rosso S, Coebergh JW, Comber H, et al Cancer incidence and mortality patterns in Europe: estimates for 40 countries in 2012. Eur J Cancer 2013 4;49(6):1374–403. doi: 10.1016/j.ejca.2012.12.027 23485231 10.1016/j.ejca.2012.12.027

[3] Gupta K, Miller JD, Li JZ, Russell MW, Charbonneau C. Epidemiologic and socioeconomic burden of metastatic renal cell carcinoma (mRCC): a literature review. Cancer Treat Rev 2008 5;34(3):193–205. doi: 10.1016/j.ctrv.2007.12.001 18313224 10.1016/j.ctrv.2007.12.001

[4] Znaor A, Lortet-Tieulent J, Laversanne M, Jemal A, Bray F. International variations and trends in renal cell carcinoma incidence and mortality. Eur Urol 2015 3;67(3):519–30. doi: 10.1016/j.eururo.2014.10.002 25449206 10.1016/j.eururo.2014.10.002

[5] Cohen HT, McGovern FJ. Renal-cell carcinoma. N Engl J Med 2005 12 8;353(23):2477–90. doi: 10.1056/NEJMra043172 16339096 10.1056/NEJMra043172

[6] Holland JM. Natural history and staging of renal cell carcinoma. CA Cancer J Clin 1975 5;25(3):121–33. 805634 10.3322/canjclin.25.3.121

[7] Escudier B, Porta C, Schmidinger M, Algaba F, Patard JJ, Khoo V, et al Renal cell carcinoma: ESMO Clinical Practice Guidelines for diagnosis, treatment and follow-up. Ann Oncol 2014 9;25 Suppl 3:iii49–iii56. 25210086 10.1093/annonc/mdu259

[8] Powles T, Staehler M, Ljungberg B, Bensalah K, Canfield SE, Dabestani S, et al Updated EAU Guidelines for Clear Cell Renal Cancer Patients Who Fail VEGF Targeted Therapy. Eur Urol 2016 1;69(1):4–6. doi: 10.1016/j.eururo.2015.10.017 26508312 10.1016/j.eururo.2015.10.017

[9] Powles T, Staehler M, Ljungberg B, Bensalah K, Canfield SE, Dabestani S, et al European Association of Urology Guidelines for Clear Cell Renal Cancers That Are Resistant to Vascular Endothelial Growth Factor Receptor-Targeted Therapy. Eur Urol 2016 6 24. 10.1016/j.eururo.2016.06.009 27349614

[10] Zhou L, Liu XD, Sun M, Zhang X, German P, Bai S, et al Targeting MET and AXL overcomes resistance to sunitinib therapy in renal cell carcinoma. Oncogene 2016 5;35(21):2687–97. doi: 10.1038/onc.2015.343 26364599 10.1038/onc.2015.343 PMC4791213

[11] Ljungberg B, Bensalah K, Bex A, Canfield S, Dabestani S, Hofmann F, et al. Guidelines on renal cell carcinoma. European Association of Urology 2014 [cited 2016 Aug 2];Available from: URL: http://uroweb.org/wp-content/uploads/10-Renal-Cell-Carcinoma_LR.pdf

[12] National Comprehensive Cancer Network. NCCN clinical practice guidelines in oncology. Kidney cancer. Version 1.2017. National Comprehensive Cancer Network 2016 September 26 [cited 2016 Sep 30];Available from: URL: https://www.nccn.org/professionals/physician_gls/f_guidelines.asp

[13] Choueiri TK, Escudier B, Powles T, Mainwaring PN, Rini BI, Donskov F, et al Cabozantinib versus Everolimus in Advanced Renal-Cell Carcinoma. N Engl J Med 2015 11 5;373(19):1814–23. doi: 10.1056/NEJMoa1510016 26406150 10.1056/NEJMoa1510016 PMC5024539

[14] Motzer RJ, Escudier B, McDermott DF, George S, Hammers HJ, Srinivas S, et al Nivolumab versus Everolimus in Advanced Renal-Cell Carcinoma. N Engl J Med 2015 11 5;373(19):1803–13. doi: 10.1056/NEJMoa1510665 26406148 10.1056/NEJMoa1510665 PMC5719487

[15] Choueiri TK, Escudier B, Powles T, Tannir NM, Mainwaring PN, Rini BI, et al. Cabozantinib versus everolimus in advanced renal cell carcinoma (METEOR): final results from a randomised, open-label, phase 3 trial. Lancet Oncol 2016 Jun 3. 10.1016/S1470-2045(16)30107-3 27279544

[16] Wiecek W, Karcher H. Nivolumab versus Cabozantinib: Comparing Overall Survival in Metastatic Renal Cell Carcinoma. PLoS One 2016;11(6):e0155389 doi: 10.1371/journal.pone.0155389 27271250 10.1371/journal.pone.0155389 PMC4894561

[17] National Institute for Health and Care Excellence. Single technology appraisal. Nivolumab for treated or metastatic renal cell carcinoma. National Institute for Health and Care Excellence 2016 May [cited 2016 Jul 26];Available from: URL: https://www.nice.org.uk/guidance/GID-TA10037/documents/committee-papers

[18] Ouwens MJ, Philips Z, Jansen JP. Network meta-analysis of parametric survival curves. Res Synth Methods 2010 7;1(3–4):258–71. doi: 10.1002/jrsm.25 26061470 10.1002/jrsm.25

[19] Guyot P, Ades AE, Ouwens MJ, Welton NJ. Enhanced secondary analysis of survival data: reconstructing the data from published Kaplan-Meier survival curves. BMC Med Res Methodol 2012;12:9 doi: 10.1186/1471-2288-12-9 22297116 10.1186/1471-2288-12-9 PMC3313891

[20] Motzer RJ, Escudier B, Oudard S, Hutson TE, Porta C, Bracarda S, et al Phase 3 trial of everolimus for metastatic renal cell carcinoma: final results and analysis of prognostic factors. Cancer 2010 9 15;116(18):4256–65. doi: 10.1002/cncr.25219 20549832 10.1002/cncr.25219

[21] Escudier B, Eisen T, Stadler WM, Szczylik C, Oudard S, Staehler M, et al Sorafenib for treatment of renal cell carcinoma: Final efficacy and safety results of the phase III treatment approaches in renal cancer global evaluation trial. J Clin Oncol 2009 7 10;27(20):3312–8. doi: 10.1200/JCO.2008.19.5511 19451442 10.1200/JCO.2008.19.5511

[22] Rini BI, Escudier B, Tomczak P, Kaprin A, Szczylik C, Hutson TE, et al Comparative effectiveness of axitinib versus sorafenib in advanced renal cell carcinoma (AXIS): a randomised phase 3 trial. Lancet 2011 12 3;378(9807):1931–9. doi: 10.1016/S0140-6736(11)61613-9 22056247 10.1016/S0140-6736(11)61613-9

[23] Spiegelhalter DJ, Best NG, Carlin BP, van der Linde A. Bayesian measures of model complexity and fit. J Royal Stat Soc Ser B 2002;64(4):583–639.

[24] Caldwell DM, Ades AE, Higgins JP. Simultaneous comparison of multiple treatments: combining direct and indirect evidence. BMJ 2005 10 15;331(7521):897–900. doi: 10.1136/bmj.331.7521.897 16223826 10.1136/bmj.331.7521.897 PMC1255806

[25] Gelman A, Rubin DB. Inference from iterative simulation using multiple sequences. Statist Sci 1992;7(4):457–72.

[26] Guo J, Nan Sheng X, Chi Z, Cui C, Si L, Ming Li S, et al A randomized, open-label, multi-center phase II study to compare bevacizumab plus sorafenib versus sorafenib for the third-line treatment of patients with metastatic renal cell carcinoma (NCT02330783). J Clin Oncol 33[15]. 2015.

[27] Hutson TE, Escudier B, Esteban E, Bjarnason GA, Lim HY, Pittman KB, et al Randomized phase III trial of temsirolimus versus sorafenib as second-line therapy after sunitinib in patients with metastatic renal cell carcinoma. J Clin Oncol 2014 3 10;32(8):760–7. doi: 10.1200/JCO.2013.50.3961 24297950 10.1200/JCO.2013.50.3961 PMC5569683

[28] Jonasch E, Corn PG, Pagliaro LC, Lara P, Wang X, Do KA, et al Randomized phase II CTEP study of MK2206 versus everolimus in VEGF inhibitor refractory renal cell carcinoma patients. J Clin Oncol 31[15 Suppl1]. 2013.

[29] Motzer RJ, Nosov D, Eisen T, Bondarenko I, Lesovoy V, Lipatov O, et al Tivozanib versus sorafenib as initial targeted therapy for patients with metastatic renal cell carcinoma: results from a phase III trial. J Clin Oncol 2013 10 20;31(30):3791–9. doi: 10.1200/JCO.2012.47.4940 24019545 10.1200/JCO.2012.47.4940 PMC5569677

[30] Motzer RJ, Porta C, Vogelzang NJ, Sternberg CN, Szczylik C, Zolnierek J, et al Dovitinib versus sorafenib for third-line targeted treatment of patients with metastatic renal cell carcinoma: an open-label, randomised phase 3 trial. Lancet Oncol 2014 3;15(3):286–96. doi: 10.1016/S1470-2045(14)70030-0 24556040 10.1016/S1470-2045(14)70030-0 PMC5719485

[31] Pal S, Azad A, Bhatia S, Drabkin H, Costello B, Sarantopoulos J, et al A Phase I/II Trial of BNC105P with Everolimus in Metastatic Renal Cell Carcinoma. Clin Cancer Res 2015 8 1;21(15):3420–7. doi: 10.1158/1078-0432.CCR-14-3370 25788492 10.1158/1078-0432.CCR-14-3370 PMC4526387

[32] Powles T, Oudard S, Escudier BJ, Brown JE, Hawkins RE, Castellano DE, et al A randomized phase II study of GDC-0980 versus everolimus in metastatic renal cell carcinoma (mRCC) patients (pts) after VEGF-targeted therapy (VEGF-TT). J Clin Oncol 32[15 Suppl1]. 2014.

[33] Powles T, Lackner MR, Oudard S, Escudier B, Ralph C, Brown JE, et al Randomized Open-Label Phase II Trial of Apitolisib (GDC-0980), a Novel Inhibitor of the PI3K/Mammalian Target of Rapamycin Pathway, Versus Everolimus in Patients With Metastatic Renal Cell Carcinoma. J Clin Oncol 2016 5 10;34(14):1660–8. doi: 10.1200/JCO.2015.64.8808 10.1200/JCO.2015.64.8808 PMC5569691 26951309

[34] Powles T, Wheater M, Din O, Geldart T, Boleti E, Stockdale A, et al A Randomised Phase 2 Study of AZD2014 Versus Everolimus in Patients with VEGF-Refractory Metastatic Clear Cell Renal Cancer. Eur Urol 2016 3;69(3):450–6. doi: 10.1016/j.eururo.2015.08.035 26364551 10.1016/j.eururo.2015.08.035

[35] Motzer RJ, Hutson TE, Glen H, Michaelson MD, Molina A, Eisen T, et al Lenvatinib, everolimus, and the combination in patients with metastatic renal cell carcinoma: a randomised, phase 2, open-label, multicentre trial. Lancet Oncol 2015 11;16(15):1473–82. doi: 10.1016/S1470-2045(15)00290-9 26482279 10.1016/S1470-2045(15)00290-9

[36] Motzer RJ, Barrios CH, Kim TM, Falcon S, Cosgriff T, Harker WG, et al Phase II randomized trial comparing sequential first-line everolimus and second-line sunitinib versus first-line sunitinib and second-line everolimus in patients with metastatic renal cell carcinoma. J Clin Oncol 2014 9 1;32(25):2765–72. doi: 10.1200/JCO.2013.54.6911 25049330 10.1200/JCO.2013.54.6911 PMC5569681

[37] Eichelberg C, Fischer Von Weikersthal L, Goebell P, LerchenmÃ¼ller C, Zimmermann U, Freier W, et al Phase III randomized sequential open-label study to evaluate efcacy and safety of sorafenib (SO) followed by sunitinib (SU) vs. sunitinib followed by sorafenib in patients with advanced/meta-static renal cell carcinoma (mRCC) without prior systemic therapy (SWITCH Study)—Safety interim analysis results. Urologe A 51[Suppl 1], 35 2012.

[38] Tannir NM, Jonasch E, Altinmakas E, Ng CS, Qiao W, Tamboli P, et al Everolimus versus sunitinib prospective evaluation in metastatic non-clear cell renal cell carcinoma (The ESPN Trial): A multicenter randomized phase 2 trial. J Clin Oncol 32[15]. 2014. 10.1016/j.eururo.2015.10.049 PMC4879109 26626617

[39] Ratain MJ, Eisen T, Stadler WM, Flaherty KT, Kaye SB, Rosner GL, et al Phase II placebo-controlled randomized discontinuation trial of sorafenib in patients with metastatic renal cell carcinoma. J Clin Oncol 2006 6 1;24(16):2505–12. doi: 10.1200/JCO.2005.03.6723 16636341 10.1200/JCO.2005.03.6723

[40] National Institute for Health and Care Excellence. NICE in development [GID-TA10037]. Renal cell carcinoma (metastatic, treated)—nivolumab [ID853]. National Institute for Health and Care Excellence 2016 October [cited 2016 Aug 3];Available from: URL: https://www.nice.org.uk/guidance/indevelopment/gid-ta10037

[41] Hoaglin DC, Hawkins N, Jansen J, Scott DA, Itzler R, Cappelleri JC. Conducting indirect treatment comparison and network meta-analysis studies: Report of the ISPOR Task Force on indirect treatment comparisons—Part 2. International Society for Pharmacoeconomics and Outcomes Research (ISPOR) 2011 [cited 2016 Aug 16];Available from: URL: http://www.ispor.org/taskforces/documents/Indirect-Treatment-Comparisons-GRP-for-Researchers-Part-2-FOR-COMMENT.pdf 10.1016/j.jval.2011.01.011 21669367

[42] Negrier S, Jager E, Porta C, McDermott D, Moore M, Bellmunt J, et al Efficacy and safety of sorafenib in patients with advanced renal cell carcinoma with and without prior cytokine therapy, a subanalysis of TARGET. Med Oncol 2010 9;27(3):899–906. doi: 10.1007/s12032-009-9303-z 19757215 10.1007/s12032-009-9303-z

[43] Korhonen P, Malangone E, Sherman S, Casciano R, Motzer RJ, Baladi J, et al Overall survival (OS) of metastatic renal cell carcinoma (mRCC) patients corrected for crossover using inverse probability of censoring weights (IPCW) and rank preserving structural failure time (RPSFT) models: Two analyses from the RECORD-1 trial. J Clin Oncol 28[15]. 2010.

[44] Hollaender N. Methods to estimate survival time after treatment switching in oncology- overview and practical considerations. Institute for Quality and Efficiency in Healthcare (IQWiG) 2014 June 27 [cited 2016 Aug 25];Available from: URL: https://www.iqwig.de/download/14-06-27_IQWiG_im_Dialog_Norbert_Hollaender.pdf

[45] Motzer RJ, Escudier B, Oudard S, Hutson TE, Porta C, Bracarda S, et al Efficacy of everolimus in advanced renal cell carcinoma: a double-blind, randomised, placebo-controlled phase III trial. Lancet 2008 8 9;372(9637):449–56. doi: 10.1016/S0140-6736(08)61039-9 18653228 10.1016/S0140-6736(08)61039-9

[46] Motzer RJ, Escudier B, Tomczak P, Hutson TE, Michaelson MD, Negrier S, et al Axitinib versus sorafenib as second-line treatment for advanced renal cell carcinoma: overall survival analysis and updated results from a randomised phase 3 trial. Lancet Oncol 2013 5;14(6):552–62. doi: 10.1016/S1470-2045(13)70093-7 23598172 10.1016/S1470-2045(13)70093-7

[47] Escudier B, Eisen T, Stadler WM, Szczylik C, Oudard S, Siebels M, et al Sorafenib in advanced clear-cell renal-cell carcinoma. N Engl J Med 2007 1 11;356(2):125–34. doi: 10.1056/NEJMoa060655 17215530 10.1056/NEJMoa060655

[48] Korhonen P, Zuber E, Branson M, Hollaender N, Yateman N, Katiskalahti T, et al Correcting overall survival for the impact of crossover via a rank-preserving structural failure time (RPSFT) model in the RECORD-1 trial of everolimus in metastatic renal-cell carcinoma. J Biopharm Stat 2012;22(6):1258–71. doi: 10.1080/10543406.2011.592233 23075021 10.1080/10543406.2011.592233

[49] Karcher H. Nivolumab versus Cabozantinib: Comparing Overall Survival in Metastatic Renal Cell Carcinoma. Reader Comment: Revised analyses with new input data for cabozantinib vs. everolimus, presented at ASCO 2016. PLoS One 2016 July 18Available from: URL: http://journals.plos.org/plosone/article/comment?id=10.1371/annotation/e22c0931-c56f-4f6a-9186-651e42c32779 10.1371/journal.pone.0155389 PMC4894561 27271250

[50] Cox C, Chu H, Schneider MF, Munoz A. Parametric survival analysis and taxonomy of hazard functions for the generalized gamma distribution. Stat Med 2007 10 15;26(23):4352–74. doi: 10.1002/sim.2836 17342754 10.1002/sim.2836

