# Supplementary material for: Cabozantinib versus everolimus, nivolumab, axitinib, sorafenib and best supportive care: A network meta-analysis of progression-free survival and overall survival in second line treatment of advanced renal cell carcinoma
Source: PLoS One. 2017 Sep 8;12(9):e0184423. doi: 10.1371/journal.pone.0184423 (PMC5590935; doi:10.1371/journal.pone.0184423)
Supplement: S2 File — (DOCX) [file pone.0184423.s002.docx]

# S2

Appendix 1. Search Strategy

**Objective**

A systematic literature search was be conducted to identify randomized controlled studies with cabozantinib and its comparators (everolimus, axitinib, nivolumab, sorafenib, sunitinib, lenvatinib) in advanced RCC. Systematic reviews, meta-analyses and HTA were searched too (for screening of reference lists only).

**Methods**

**PICOS Framework**

| **Category** | **Details** |
| --- | --- |
| Population | Renal cell cancer (advanced / metastatic, previously treated) |
| Interventions | Cabozantinib |
| Comparators | Everolimus, axitinib, nivolumab, sorafenib, sunitinib, lenvatinib |
| Outcomes | PFS, OS, complete or partial response,, drug discontinuation, opioid use, other safety outcomes, clinical benefit (RR+SD), duration of response, rate of progression (as best response)/ rate of refractory disease, quality of life and other PRO, biomarkers for efficacy and safety |
| Study Design | RCT  Systematic reviews, meta-analyses, HTA (identified for checking the reference lists [for screening reference lists in respect of identification of additional, relevant studies] |

**Further parameters and restrictions**

| Timeframe of Search | No time restriction |
| --- | --- |
| Language | No language restriction |
| Exclude | Animal Studies |

**Bibliographic databases searched**

| Databases | Date of Search |
| --- | --- |
| Medline (includes Medline in Process and other non-indexed citations with status: publisher, in-data review or Pubmed-not-Medline) | Jun 03, 2016 |
| Embase | Jun 03, 2016 |
| Cochrane Library (includes Cochrane Central Register of Controlled Trials, Cochrane Reviews, DARE, HTA Database, NHSEED) | Jun 03, 2016 |

**Search Terms per Database**

**Medline (includes Medline in Process and other non-indexed citations (with status: publisher, in-data review or Pubmed-not-Medline))**

**Renal Cell Cancer**

| **Search Terms Medline, Pos. 10** |
| --- |
| CT=CARCINOMA, RENAL CELL |
| CT=KIDNEY NEOPLASMS |
| RENAL # # (CARCINOMA#; ADENOCARCINOMA#; CANCER#; NEOPLASM#; TUMO#R#; MALIGNANC###)/(TI; AB; UT) |
| (CARCINOMA#; ADENOCARCINOMA#; CANCER#; NEOPLASM#; TUMO#R#; MALIGNANC###) # RENAL/(TI; AB; UT) |
| KIDNEY # # (CARCINOMA#; ADENOCARCINOMA#; CANCER#; NEOPLASM#; TUMO#R#; MALIGNANC###)/(TI; AB; UT) |
| (CARCINOMA#; ADENOCARCINOMA#; CANCER#; NEOPLASM#; TUMO#R#; MALIGNANC###) # # KIDNEY/(TI; AB; UT) |
| (HYPERNEPHROMA# OR NEPHROID CARCINOMA# OR HYPERNEPHROID CARCINOMA# OR GRAWITZ TUMO#R)/(TI; AB; UT) |
| RCC/(TI; AB; UT) OR MRCC/(TI; AB; UT) |

**Cabozantinib, Everolimus, Axitinib, Nivolumab, Sorafenib, Sunitinib, Lenvatinib**

| **Search Terms Medline, Pos. 47** |
| --- |
| TE=CABOZANTINIB |
| CABOZANTINIB?/(TI; AB; UT) |
| (BMS 907351 OR BMS907351 OR XL 184 OR XL184 OR CABOMETYX? OR COMETRIQ?)/(TI; AB; UT) |
| RNO=1C39JW444G OR RNO=DR7ST46X58 |
| CR=1140909-48-3 OR CR=849217-68-1 |
| CT=EVEROLIMUS |
| EVEROLIMUS?/(TI; AB; UT) |
| (RAD 001 OR RAD001 OR SDZ RAD OR AFINITOR? OR CERTICAN? OR ZORTRESS? OR VOTUBIA?)/(TI; AB; UT) |
| RNO=9HW64Q8G6G |
| CR=159351-69-6 |
| TE=AXITINIB |
| AXITINIB?/(TI; AB; UT) |
| (AG 013736 OR AG013736 OR INLYTA?)/(TI; AB; UT) |
| RNO=C9LVQ0YUXG |
| CR=319460-85-0 |
| TE=NIVOLUMAB |
| NIVOLUMAB?/(TI; AB; UT) |
| (MDX-1106 OR MDX1106 OR ONO-4538 OR ONO4538 OR BMS-936558 OR BMS936558 OR OPDIVO?)/(TI; AB; UT) |
| RNO=31YO63LBSN |
| CR=946414-94-4 |
| TE=SORAFENIB |
| SORAFENIB?/(TI; AB; UT) |
| (BAY 43-9006 OR BAY 439006 OR BAY43 9006 OR BAY439006 OR BAY 545-9085 OR BAY 5459085 OR BAY545-9085 OR BAY5459085 OR BAY 54-9085 OR BAY 549085 OR BAY54-9085 OR BAY549085 OR NEXAVAR?)/(TI; AB; UT) |
| RNO=5T62Q3B36J OR RNO=9ZOQ3TZI87 |
| CR=284461-73-0 OR CR=475207-59-1 |
| TE=SUNITINIB |
| SUNITINIB?/(TI; AB; UT) |
| (PHA-290940AD OR PHA290940AD OR SU 011248 OR SU011248 OR SU 11248 OR SU11248 OR SU010398 OR SU 010398 OR SU 10398 OR SU10398 OR SUTENT?)/(TI; AB; UT) |
| RNO=LVX8N1UT73 |
| CR=341031-54-7 |
| TE=LENVATINIB |
| LENVATINIB?/(TI; AB; UT) |
| (LENVIMA? OR E7080 OR E 7080 OR ER-203492-00)/(TI; AB; UT) |
| RNO=EE083865G2 |
| CR=417716-92-8 |
| CT=PROTEIN KINASE INHIBITORS |

**Randomized Controlled Trials**

Cochrane Highly Sensitive Search Strategy for identifying randomized trials in MEDLINE: sensitivity-maximizing version (2008 revision); PubMed format (adapted to DIMDI format)

*Source: Higgins JPT, Green S (editors). Cochrane Handbook for Systematic Reviews of Interventions Version 5.1.0 [updated March 2011]. The Cochrane Collaboration, 2011. Available from:* [*www.cochrane-handbook.org*](file:///\\la-ser.lan\DFS\LORRACH\Projects\_Active%20Projects\Ipsen\Cabozantinib%20EU%20MA%202016\06%20Systematic%20Literature%20Review\02%20Clinical%20Evidence\01%20Search%20Strategy\www.cochrane-handbook.org)*.*

| **Search Terms Medline, Pos.58** |
| --- |
| DT=RANDOMIZED CONTROLLED TRIAL |
| DT=CONTROLLED CLINICAL TRIAL |
| RANDOMI%ED/(TI;AB;UT) |
| PLACEBO/(TI;AB;UT) |
| QF=DRUG THERAPY |
| RANDOMLY/(TI;AB) |
| TRIAL/(TI;AB) |
| GROUPS/(TI;AB) |
| .. NOT (CT D ANIMALS NOT CT=HUMANS) |

**Systematic Reviews, Meta-Analyses, HTA**

SIGN Search Strategy Systematic Reviews, OVID format (adapted to DIMDI format)

*Source: Scottish Intermediate Guidelines Network (SIGN), Filter Systematic Reviews*

*URL:* [*http://www.sign.ac.uk/methodology/filters.html#systematic*](http://www.sign.ac.uk/methodology/filters.html%23systematic), [*Website last modified 27/08/15]*

| **Search Terms Medline, Pos. 92** |
| --- |
| CT=META-ANALYSIS AS TOPIC |
| (META-ANALY? OR METAANALY?)/(TI;AB;UT) |
| DT=META-ANALYSIS |
| SYSTEMATIC # (REVIEW#;OVERVIEW#)/(TI;AB;UT) |
| CT D REVIEW LITERATURE AS TOPIC |
| COCHRANE/AB |
| EMBASE/AB |
| (PSYCHLIT OR PSYCLIT)/AB |
| (PSYCHINFO OR PSYCINFO)/AB |
| (CINAHL OR CINHAL)/AB |
| SCIENCE CITATION INDEX/AB |
| REFERENCE LIST?/AB |
| BIBLIOGRAPH?/AB |
| HAND-SEARCH?/AB |
| RELEVANT JOURNALS/AB |
| MANUAL SEARCH?/AB |
| SELECTION CRITERIA/AB. AND DT=REVIEW |
| DATA EXTRACTION/AB AND DT=REVIEW |
| NOT (DT=COMMENT OR DT=LETTER OR DT=EDITORIAL) |
| NOT (CT D ANIMALS NOT CT=HUMANS) |

**Embase**

**Renal Cell Cancer**

| **Search Terms Embase, Pos. #6** |
| --- |
| 'kidney carcinoma'/exp |
| 'kidney tumor'/exp |
| (renal NEAR/3 (carcinoma* OR adenocarcinoma* OR cancer* OR neoplasm* OR tumor* OR tumour*)):ab,ti |
| (kidney NEAR/3 (carcinoma* OR adenocarcinoma* OR cancer* OR neoplasm* OR tumor* OR tumour*)):ab,ti |
| mrcc:ab,ti OR rcc:ab,ti OR hypernephroma*:ab,ti OR (nephroid NEXT/1 carcinoma*):ab,ti OR (hypernephroid NEXT/1 carcinoma*):ab,ti OR (grawitz NEXT/1 tumor*):ab,ti OR (grawitz NEXT/1 tumour*):ab,ti |

**Cabozantinib, Everolimus, Axitinib, Nivolumab, Sorafenib, Sunitinib, Lenvatinib**

| **Search Terms Embase, Pos.#36** |
| --- |
| 'cabozantinib'/de |
| cabozantinib*:tn,ab,ti |
| 'bms 907351':tn,ab,ti OR bms907351:tn,ab,ti OR 'xl 184':tn,ab,ti OR xl184:tn,ab,ti OR cabometyx*:tn,ab,ti OR cometriq*:tn,ab,ti |
| '1140909-48-3':rn OR '849217-68-1':rn |
| 'everolimus'/de |
| everolimus*:tn,ab,ti |
| 'rad 001':tn,ab,ti OR rad001:tn,ab,ti OR 'sdz rad':tn,ab,ti OR afinitor*:tn,ab,ti OR zortress*:tn,ab,ti OR votubia*:tn,ab,ti |
| '159351-69-6':rn |
| 'axitinib':de |
| axitinib*:tn,ab,ti |
| 'ag 013736':tn,ab,ti OR ag013736:tn,ab,ti OR inlyta:tn,ab,ti |
| '319460-85-0':rn |
| 'nivolumab'/de |
| nivolumab*:tn,ab,ti |
| 'mdx-1106':tn,ab,ti OR mdx1106:tn,ab,ti OR 'ono-4538':tn,ab,ti OR ono4538:tn,ab,ti OR 'bms-936558':tn,ab,ti OR bms936558:tn,ab,ti OR opdivo*:tn,ab,ti |
| '946414-94-4':rn |
| 'sorafenib'/de |
| sorafenib*:tn,ab,ti |
| 'bay 43-9006':tn,ab,ti OR 'bay 439006':tn,ab,ti OR 'bay43 9006':tn,ab,ti OR bay439006:tn,ab,ti OR 'bay 545-9085':tn,ab,ti OR 'bay 5459085':tn,ab,ti OR 'bay545-9085':tn,ab,ti OR bay5459085:tn,ab,ti OR 'bay 54-9085':tn,ab,ti OR 'bay 549085':tn,ab,ti OR 'bay54-9085':tn,ab,ti OR bay549085:tn,ab,ti OR nexavar:tn,ab,ti |
| '284461-73-0':rn OR '475207-59-1':rn |
| 'sunitinib'/de |
| sunitinib*:tn,ab,ti |
| 'pha-290940ad':tn,ab,ti OR 'pha290940ad':tn,ab,ti OR 'su 011248':tn,ab,ti OR 'su011248':tn,ab,ti OR 'su 11248':tn,ab,ti OR 'su11248':tn,ab,ti OR 'su010398':tn,ab,ti OR 'su 010398':tn,ab,ti OR 'su 10398':tn,ab,ti OR 'su10398':tn,ab,ti OR sutent*:tn,ab,ti |
| '341031-54-7':rn |
| 'lenvatinib'/de |
| lenvatinib*:tn,ab,ti |
| 'e7080':tn,ab,ti OR 'e 7080':tn,ab,ti OR 'er-203492-00':tn,ab,ti OR lenvima*:tn,ab,ti |
| '417716-92-8':rn |
| 'protein kinase inhibitor'/de OR 'protein serine threonine kinase inhibitor'/de OR 'protein tyrosine kinase inhibitor'/de |

**Randomized Controlled Trials**

SIGN Search Strategy Randomized Controlled Trials, OVID format (adapted to DIMDI format)

*Source: Scottish Intermediate Guidelines Network (SIGN), Filter Randomized Controlled Trials*

*URL:* [*http://www.sign.ac.uk/methodology/filters.html#random*](http://www.sign.ac.uk/methodology/filters.html%23random) *[cited 20160512]*

| **Search Terms Embase, Pos.#54** |
| --- |
| ‘Clinical trial’/de |
| ‘Randomized controlled trial’/de |
| Randomization/de |
| ‘Single blind procedure’/de |
| ‘Double blind procedure’/de |
| ‘Crossover procedure’/de |
| Placebo/de |
| ((Randomized OR randomised) NEXT/1 controlled NEXT/1 trial*):ab,ti,tn |
| Rct:ab,ti,tn |
| ‘Random allocation’:ab,ti,tn OR ‘Randomly allocated':ab,ti,tn OR ‘allocated randomly’:ab,ti,tn |
| (Allocated NEAR/2 RANDOM):ab,ti,tn |
| ((Single OR double) NEXT/1 blind*):ab,ti,tn |
| ((Treble OR Triple) NEXT/1 blind*):ab,ti,tn |
| Placebo*:ab,ti,tn |
| ‘Prospective study’/de |
| .. NOT (‘Case study’/de OR ‘Case report’:ab,ti,dn or ‘Abstract report’:it or letter:it) |

**Systematic Reviews**

SIGN Search Strategy Systematic Reviews, OVID format (adapted to DIMDI format)

*Source: Scottish Intermediate Guidelines Network (SIGN), Filter Systematic Reviews*

*URL:* [http://www.sign.ac.uk/methodology/filters.html#systematic](http://www.sign.ac.uk/methodology/filters.html%23systematic) *[cited 20160512]*

| **Search Terms Embase, Pos.#87** |
| --- |
| 'meta analysis'/exp |
| (meta NEXT/1 analy*):ab,ti,tn OR metaanalys*:ab,ti,tn |
| (systematic NEXT/1 (review* OR overview*)):ab,ti,tn |
| cancerlit:ab |
| cochrane:ab |
| embase:ab |
| psychlit:ab OR psyclit:ab |
| psychinfo:ab OR psycinfo:ab |
| cinahl:ab OR cinhal:ab |
| 'science citation index':ab |
| bids:ab |
| 'reference lists':ab |
| bibliograph*:ab |
| (hand NEXT/1 search*):ab |
| (manual NEXT/1 search*):ab |
| 'relevant journals':ab |
| 'data extraction':ab AND ('review'/exp OR review:it) |
| 'selection criteria':ab AND ('review'/exp OR review:it) |
| NOT (letter:it OR editorial:it OR ('animal'/exp NOT ('animal'/exp NOT 'human'/exp)) |

**Cochrane Library**

**Renal Cell Cancer**

| **Search Terms Cochrane Library, Pos. #6** |
| --- |
| [mh ^"Carcinoma, Renal Cell"] |
| [mh ^"kidney neoplasms"] |
| (renal near/3 (carcinoma* or adenocarcinoma* or cancer* or neoplasm* or tumor* or tumour*)):ab,ti,kw |
| (kidney near/3 (carcinoma* or adenocarcinoma* or cancer* or neoplasm* or tumor* or tumour*)):ab,ti,kw |
| mrcc:ab,ti,kw or rcc:ab,ti,kw or hypernephroma*:ab,ti,kw or (nephroid next/1 carcinoma*):ab,ti,kw or (hypernephroid next/1 carcinoma*):ab,ti,kw or (grawitz next/1 tumor*):ab,ti,kw or (grawitz next/1 tumour*):ab,ti,kw |

**Cabozantinib, Everolimus, Axitinib, Nivolumab, Sorafenib, Sunitinib, Lenvatinib**

| **Search Terms Cochrane Library, Pos. #24** |
| --- |
| cabozantinib*:ab,ti,kw |
| ("bms 907351" or bms907351 or "xl 184" or xl184 or cabometyx* or cometriq*):ab,ti,kw |
| [mh everolimus] |
| everolimus*:ab,ti,kw |
| ("rad 001" or rad001 or "sdz rad" or afinitor* or certican* or zortress* or votubia*):ab,ti,kw |
| axitinib*:ab,ti,kw |
| ("ag 013736" or ag013736 or inlyta*):ab,ti,kw |
| nivolumab*:ab,ti,kw |
| ("mdx-1106" or mdx1106 or "ono-4538" or ono4538 or "bms-936558" or bms936558 or opdivo*):ab,ti,kw |
| sorafenib:ab,ti,kw |
| ("bay 43-9006" or bay 439006 or "bay43 9006" or bay439006 or "bay 545-9085" or "bay 5459085" or "bay545-9085" or bay5459085 or "bay 54-9085" or "bay 549085" or "bay54-9085" or bay549085 or nexavar*):ab,ti,kw |
| Sunitinib*:ab,ti,kw |
| (sutent* or "pha-290940ad" or pha290940ad or "su 011248" or su011248 or "su 11248" or su11248 or su010398 or "su 010398" or "su 10398" or su10398):ab,ti,kw |
| Lenvatinib*:ab,ti,kw |
| ("E7080" or "E 7080" or "ER-203492-00" or lenvima*) ab,ti,kw |
| [mh ^"Protein Kinase Inhibitors"] |
| "protein kinase inhibitor":kw or "protein serine threonine kinase inhibitor":kw or "protein tyrosine kinase inhibitor":kw |

**Results**

**Overview of the results**

| **Database** | **RCT** | **Systematic Reviews** | **Combined Finding s RCT + Systematic Reviews / Meta-Analyses / HTA** |
| --- | --- | --- | --- |
| Medline | 2539 | 107 | 2556 |
| Embase | 3482 | 298 | 3598 |
| Cochrane Library | - | - | 458 |
| Total |  |  | 6612 (including duplicates) |

**Results per database**

***Medline (includes Medline in Process and other non-indexed citations (with status: publisher, in-data review or Pubmed-not-Medline)***

| Renal Cell Cancer |  | 10 | 75005 |
| --- | --- | --- | --- |
| AND Cabozantinib, Everolimus, Axitinib, Nivolumab, Sorafenib, Sunitinib, lenvatinib | 10 AND 47 | 48 | 3393 |
| AND RCT (COCHRANE HIGHLY SENSITIVE SEARCH STRATEGY FOR IDENTIFYING RANDOMIZED TRIALS IN MEDLINE: SENSITIVITY-MAXIMIZING VERSION (2008 REVISION); OVID FORMAT (APDAPTED TO DIMDI FORMAT)) | 48 AND 58 | 59 | 2539 |
| AND Systematic Reviews (SIGN FILTER) | 48 AND 92 | 93 | 107 |
| **Combined Results (RCT + Systematic Reviews etc.)** | **59 OR 93** | **94** | **2556** |

***Embase***

| **Short description** | **Search Concepts and Combinations** | **Position in Search Protocol** | **No. of findings** |
| --- | --- | --- | --- |
|  |  |  |  |
| Renal Cell Cancer |  | 6 | 124831 |
| AND Cabozantinib, Everolimus, Axitinib, Nivolumab, Sorafenib, Sunitinib, lenvatinib | #6 AND #36 | #37 | 11260 |
| AND RCT (SIGN Filter) | #37 AND #54 | #55 | 3482 |
| AND SYSTEMATIC REVIEWS (SIGN Filter) | #37 AND #87 | #88 | 298 |
| **Combined Results (RCT + Systematic Reviews etc.)** | **#55 OR #88** | **#89** | **3598** |

***Cochrane Library***

| **Short description** | **Search Concepts and Combinations** | **Position in Search Protocol** | **No. of findings** |
| --- | --- | --- | --- |
|  |  |  |  |
| Renal Cell Cancer |  | #6 | 1861 |
| AND Cabozantinib, Everolimus, Axitinib, Nivolumab, Sorafenib, Sunitinib, lenvatinib | #6 and #24 | #25 | 458 |
| **Cochrane Library (includes Cochrane Central Register of Controlled Trials, Cochrane Reviews, DARE, HTA Database, NHSEED)** |  | **#26** | **458** |

**Search Strategies by Database**

***Search Strategy Medline including Medline in Process (and other non-indexed citations with status: publisher, in-data review or Pubmed-not-Medline****)*

| Database | | Medline (includes Medline in Process and other non-indexed citations (with status: publisher, in-data review or Pubmed-not-Medline) | |
| --- | --- | --- | --- |
| Search Platform: | | DIMDI Classic Search | |
| Date of Search: | | June 3, 2016 [Last Database Update: June 3, 2016] | |
| Date Range Searched: | | 1966-2016 | |
| Search Filters Used | | RCT: Cochrane Highly Sensitive Search Strategy for identifying randomized trials in MEDLINE: sensitivity-maximizing version  Systematic Reviews: SIGN Search Filter Systematic Reviews | |
| # | **Search Terms** | | **Hits** |
| 1 | ME66 | | 23722961 |
| 2 | CT=CARCINOMA, RENAL CELL | | 25382 |
| 3 | CT=KIDNEY NEOPLASMS | | 56261 |
| 4 | RENAL # # (CARCINOMA#; ADENOCARCINOMA#; CANCER#; NEOPLASM#; TUMO#R#; MALIGNANC###)/(TI; AB; UT) | | 43314 |
| 5 | (CARCINOMA#; ADENOCARCINOMA#; CANCER#; NEOPLASM#; TUMO#R#; MALIGNANC###) # RENAL/(TI; AB; UT) | | 3977 |
| 6 | KIDNEY # # (CARCINOMA#; ADENOCARCINOMA#; CANCER#; NEOPLASM#; TUMO#R#; MALIGNANC###)/(TI; AB; UT) | | 7468 |
| 7 | (CARCINOMA#; ADENOCARCINOMA#; CANCER#; NEOPLASM#; TUMO#R#; MALIGNANC###) # # KIDNEY/(TI; AB; UT) | | 5072 |
| 8 | (HYPERNEPHROMA# OR NEPHROID CARCINOMA# OR HYPERNEPHROID CARCINOMA# OR GRAWITZ TUMO#R)/(TI; AB; UT) | | 1108 |
| 9 | RCC/(TI; AB; UT) OR MRCC/(TI; AB; UT) | | 11303 |
| 10 | 2 TO 9 | | 75005 |
| 11 | TE=CABOZANTINIB | | 100 |
| 12 | CABOZANTINIB?/(TI; AB; UT) | | 247 |
| 13 | (BMS 907351 OR BMS907351 OR XL 184 OR XL184 OR CABOMETYX? OR COMETRIQ?)/(TI; AB; UT) | | 65 |
| 14 | RNO=1C39JW444G OR RNO=DR7ST46X58 | | 100 |
| 15 | CR=1140909-48-3 OR CR=849217-68-1 | | 0 |
| 16 | CT=EVEROLIMUS | | 2845 |
| 17 | EVEROLIMUS?/(TI; AB; UT) | | 4002 |
| 18 | (RAD 001 OR RAD001 OR SDZ RAD OR AFINITOR? OR CERTICAN? OR ZORTRESS? OR VOTUBIA?)/(TI; AB; UT) | | 599 |
| 19 | RNO=9HW64Q8G6G | | 2845 |
| 20 | CR=159351-69-6 | | 0 |
| 21 | TE=AXITINIB | | 259 |
| 22 | AXITINIB?/(TI; AB; UT) | | 484 |
| 23 | (AG 013736 OR AG013736 OR INLYTA?)/(TI; AB; UT) | | 54 |
| 24 | RNO=C9LVQ0YUXG | | 259 |
| 25 | CR=319460-85-0 | | 0 |
| 26 | TE=NIVOLUMAB | | 152 |
| 27 | NIVOLUMAB?/(TI; AB; UT) | | 427 |
| 28 | (MDX-1106 OR MDX1106 OR ONO-4538 OR ONO4538 OR BMS-936558 OR BMS936558 OR OPDIVO?)/(TI; AB; UT) | | 41 |
| 29 | RNO=31YO63LBSN | | 152 |
| 30 | CR=946414-94-4 | | 0 |
| 31 | TE=SORAFENIB | | 3100 |
| 32 | SORAFENIB?/(TI; AB; UT) | | 5022 |
| 33 | (BAY 43-9006 OR BAY 439006 OR BAY43 9006 OR BAY439006 OR BAY 545-9085 OR BAY 5459085 OR BAY545-9085 OR BAY5459085 OR BAY 54-9085 OR BAY 549085 OR BAY54-9085 OR BAY549085 OR NEXAVAR?)/(TI; AB; UT) | | 270 |
| 34 | RNO=5T62Q3B36J OR RNO=9ZOQ3TZI87 | | 3100 |
| 35 | CR=284461-73-0 OR CR=475207-59-1 | | 0 |
| 36 | TE=SUNITINIB | | 2489 |
| 37 | SUNITINIB?/(TI; AB; UT) | | 3873 |
| 38 | (PHA-290940AD OR PHA290940AD OR SU 011248 OR SU011248 OR SU 11248 OR SU11248 OR SU010398 OR SU 010398 OR SU 10398 OR SU10398 OR SUTENT?)/(TI; AB; UT) | | 255 |
| 39 | RNO=LVX8N1UT73 | | 0 |
| 40 | CR=341031-54-7 | | 0 |
| 41 | TE=LENVATINIB | | 57 |
| 42 | LENVATINIB?/(TI; AB; UT) | | 103 |
| 43 | (LENVIMA? OR E7080 OR E 7080 OR ER-203492-00)/(TI; AB; UT) | | 43 |
| 44 | RNO=EE083865G2 | | 57 |
| 45 | CR=417716-92-8 | | 0 |
| 46 | CT=PROTEIN KINASE INHIBITORS | | 30047 |
| 47 | 11 TO 46 | | 41423 |
| 48 | 10 AND 47 | | 3393 |
| 49 | DT=RANDOMIZED CONTROLLED TRIAL | | 412252 |
| 50 | DT=CONTROLLED CLINICAL TRIAL | | 90287 |
| 51 | RANDOMI%ED/(TI; AB; UT) | | 449770 |
| 52 | PLACEBO/(TI; AB; UT) | | 175332 |
| 53 | QF=DRUG THERAPY | | 1835793 |
| 54 | RANDOMLY/(TI; AB) | | 253055 |
| 55 | TRIAL/(TI; AB) | | 424170 |
| 56 | GROUPS/(TI; AB) | | 1588506 |
| 57 | 49 TO 56 | | 3797749 |
| 58 | 57 NOT (CT D ANIMALS NOT CT=HUMANS) | | 3275978 |
| 59 | 48 AND 58 | | 2539 |
| 60 | CT=META-ANALYSIS AS TOPIC | | 14690 |
| 61 | (META-ANALY? OR METAANALY?)/(TI; AB; UT) | | 94481 |
| 62 | DT=META-ANALYSIS | | 64644 |
| 63 | SYSTEMATIC # (REVIEW#; OVERVIEW#)/(TI; AB; UT) | | 87628 |
| 64 | CT D REVIEW LITERATURE AS TOPIC | | 8547 |
| 65 | 60 TO 64 | | 175414 |
| 66 | COCHRANE/AB | | 44067 |
| 67 | EMBASE/AB | | 45124 |
| 68 | (PSYCHLIT OR PSYCLIT)/AB | | 891 |
| 69 | (PSYCHINFO OR PSYCINFO)/AB | | 13009 |
| 70 | (CINAHL OR CINHAL)/AB | | 14910 |
| 71 | SCIENCE CITATION INDEX/AB | | 2358 |
| 72 | 66 TO 71 | | 72089 |
| 73 | REFERENCE LIST?/AB | | 12313 |
| 74 | BIBLIOGRAPH?/AB | | 13533 |
| 75 | HAND-SEARCH?/AB | | 4885 |
| 76 | RELEVANT JOURNALS/AB | | 879 |
| 77 | MANUAL SEARCH?/AB | | 3030 |
| 78 | 73 TO 77 | | 31036 |
| 79 | SELECTION CRITERIA/AB | | 23188 |
| 80 | DATA EXTRACTION/AB | | 12580 |
| 81 | 79 OR 80 | | 33953 |
| 82 | DT=REVIEW | | 2095686 |
| 83 | 81 AND 82 | | 22438 |
| 84 | DT=LETTER | | 917385 |
| 85 | DT=EDITORIAL | | 402915 |
| 86 | 84 OR 85 | | 1320221 |
| 87 | CT D ANIMALS | | 18398988 |
| 88 | CT=HUMANS | | 14382672 |
| 89 | 87 NOT (87 AND 88) | | 4016316 |
| 90 | 86 OR 89 | | 5294613 |
| 91 | 65 OR 72 OR 78 OR 83 | | 209441 |
| 92 | 91 NOT 90 | | 199564 |
| 93 | 48 AND 92 | | 107 |
| 94 | **59 OR 93** | | **2556** |

**Search Strategy Embase**

| Database | | Embase | |
| --- | --- | --- | --- |
| Search Platform: | | Embase.com | |
| Date of Search: | | June 3, 2016 [Last Database Update: June 3, 2016 | |
| Date Range Searched: | | 1974-2016 | |
| Search Filters Used | | RCT: SIGN Search Filter Randomized Controlled Studies  Systematic Reviews: SIGN Search Filter Systematic Reviews | |
| # | **Search Terms** | | **Hits** |
| #1 | 'kidney carcinoma'/exp | | 53140 |
| #2 | 'kidney tumor'/exp | | 109794 |
| #3 | (renal NEAR/3 (carcinoma* OR adenocarcinoma* OR cancer* OR neoplasm* OR tumor* OR tumour*)):ab,ti | | 64350 |
| #4 | (kidney NEAR/3 (carcinoma* OR adenocarcinoma* OR cancer* OR neoplasm* OR tumor* OR tumour*)):ab,ti | | 15655 |
| #5 | mrcc:ab,ti OR rcc:ab,ti OR hypernephroma*:ab,ti OR (nephroid NEXT/1 carcinoma*):ab,ti OR (hypernephroid NEXT/1 carcinoma*):ab,ti OR (grawitz NEXT/1 tumor*):ab,ti OR (grawitz NEXT/1 tumour*):ab,ti | | 20539 |
| #6 | #1 OR #2 OR #3 OR #4 OR #5 | | 124831 |
| #7 | 'cabozantinib'/de | | 1456 |
| #8 | cabozantinib*:tn,ab,ti | | 453 |
| #9 | 'bms 907351':tn,ab,ti OR bms907351:tn,ab,ti OR 'xl 184':tn,ab,ti OR xl184:tn,ab,ti OR cabometyx*:tn,ab,ti OR cometriq*:tn,ab,ti | | 675 |
| #10 | '1140909-48-3':rn OR '849217-68-1':rn | | 993 |
| #11 | 'everolimus'/de | | 18432 |
| #12 | everolimus*:tn,ab,ti | | 9385 |
| #13 | 'rad 001':tn,ab,ti OR rad001:tn,ab,ti OR 'sdz rad':tn,ab,ti OR afinitor*:tn,ab,ti OR zortress*:tn,ab,ti OR votubia*:tn,ab,ti | | 3234 |
| #14 | '159351-69-6':rn | | 12624 |
| #15 | 'axitinib':de | | 2807 |
| #16 | axitinib*:tn,ab,ti | | 964 |
| #17 | 'ag 013736':tn,ab,ti OR ag013736:tn,ab,ti OR inlyta:tn,ab,ti | | 719 |
| #18 | '319460-85-0':rn | | 2299 |
| #19 | 'nivolumab'/de | | 1852 |
| #20 | nivolumab*:tn,ab,ti | | 682 |
| #21 | 'mdx-1106':tn,ab,ti OR mdx1106:tn,ab,ti OR 'ono-4538':tn,ab,ti OR ono4538:tn,ab,ti OR 'bms-936558':tn,ab,ti OR bms936558:tn,ab,ti OR opdivo*:tn,ab,ti | | 585 |
| #22 | '946414-94-4':rn | | 1366 |
| #23 | 'sorafenib'/de | | 19687 |
| #24 | sorafenib*:tn,ab,ti | | 9811 |
| #25 | 'bay 43-9006':tn,ab,ti OR 'bay 439006':tn,ab,ti OR 'bay43 9006':tn,ab,ti OR bay439006:tn,ab,ti OR 'bay 545-9085':tn,ab,ti OR 'bay 5459085':tn,ab,ti OR 'bay545-9085':tn,ab,ti OR bay5459085:tn,ab,ti OR 'bay 54-9085':tn,ab,ti OR 'bay 549085':tn,ab,ti OR 'bay54-9085':tn,ab,ti OR bay549085:tn,ab,ti OR nexavar:tn,ab,ti | | 3554 |
| #26 | '284461-73-0':rn OR '475207-59-1':rn | | 15065 |
| #27 | 'sunitinib'/de | | 16366 |
| #28 | sunitinib*:tn,ab,ti | | 7460 |
| #29 | 'pha-290940ad':tn,ab,ti OR 'pha290940ad':tn,ab,ti OR 'su 011248':tn,ab,ti OR 'su011248':tn,ab,ti OR 'su 11248':tn,ab,ti OR 'su11248':tn,ab,ti OR 'su010398':tn,ab,ti OR 'su 010398':tn,ab,ti OR 'su 10398':tn,ab,ti OR 'su10398':tn,ab,ti OR sutent*:tn,ab,ti | | 3413 |
| #30 | '341031-54-7':rn | | 12932 |
| #31 | 'lenvatinib'/de | | 505 |
| #32 | lenvatinib*:tn,ab,ti | | 197 |
| #33 | 'e7080':tn,ab,ti OR 'e 7080':tn,ab,ti OR 'er-203492-00':tn,ab,ti OR lenvima*:tn,ab,ti | | 242 |
| #34 | '417716-92-8':rn | | 349 |
| #35 | 'protein kinase inhibitor'/de OR 'protein serine threonine kinase inhibitor'/de OR 'protein tyrosine kinase inhibitor'/de | | 34258 |
| #36 | #7 OR #8 OR #9 OR #10 OR #11 OR #12 OR #13 OR #14 OR #15 OR #16 OR #17 OR #18 OR #19 OR #20 OR #21 OR #22 OR #23 OR #24 OR #25 OR #26 OR #27 OR #28 OR #29 OR #30 OR #31 OR #32 OR #33 OR #34 OR #35 | | 75686 |
| #37 | #6 AND #36 | | 11260 |
| #38 | 'clinical trial'/de | | 859671 |
| #39 | 'randomized controlled trial'/de | | 402299 |
| #40 | 'randomization'/de | | 69802 |
| #41 | 'single blind procedure'/de | | 22057 |
| #42 | 'double blind procedure'/de | | 128878 |
| #43 | 'crossover procedure'/de | | 46791 |
| #44 | 'placebo'/de | | 289437 |
| #45 | ((randomized OR randomised) NEXT/1 controlled NEXT/1 trial*):tn,ab,ti | | 135585 |
| #46 | rct:tn,ab,ti | | 20611 |
| #47 | 'random allocation':tn,ab,ti OR 'randomly allocated':tn,ab,ti OR 'allocated randomly':tn,ab,ti | | 28517 |
| #48 | (allocated NEAR/2 random):tn,ab,ti | | 837 |
| #49 | ((single OR double) NEXT/1 blind*):tn,ab,ti | | 183656 |
| #50 | ((treble OR triple) NEXT/1 blind*):tn,ab,ti | | 575 |
| #51 | placebo*:tn,ab,ti | | 237163 |
| #52 | 'prospective study'/de | | 325975 |
| #53 | #38 OR #39 OR #40 OR #41 OR #42 OR #43 OR #44 OR #45 OR #46 OR #47 OR #48 OR #49 OR #50 OR #51 OR #52 | | 1582043 |
| #54 | #53 NOT ('case study'/de OR 'case report':tn,ab,ti OR 'abstract report':it OR letter:it) | | 1542170 |
| #55 | #37 AND #54 | | 3482 |
| #56 | 'meta analysis'/exp | | 108931 |
| #57 | (meta NEAR/1 analy*):tn,ab,ti OR metaanalys*:tn,ab,ti | | 119809 |
| #58 | (systematic NEXT/1 (review* OR overview*)):tn,ab,ti | | 99716 |
| #59 | #56 OR #57 OR #58 | | 212763 |
| #60 | cancerlit:ab | | 672 |
| #61 | cochrane:ab | | 53904 |
| #62 | embase:ab | | 54166 |
| #63 | psychlit:ab OR psyclit:ab | | 956 |
| #64 | psychinfo:ab OR psycinfo:ab | | 13200 |
| #65 | cinahl:ab OR cinhal:ab | | 16412 |
| #66 | 'science citation index':ab | | 2625 |
| #67 | bids:ab | | 496 |
| #68 | #60 OR #61 OR #62 OR #63 OR #64 OR #65 OR #66 OR #67 | | 86050 |
| #69 | 'reference lists':ab | | 12686 |
| #70 | bibliograph*:ab | | 16934 |
| #71 | (hand NEXT/1 search*):ab | | 5622 |
| #72 | (manual NEXT/1 search*):ab | | 3453 |
| #73 | 'relevant journals':ab | | 1015 |
| #74 | #69 OR #70 OR #71 OR #72 OR #73 | | 35774 |
| #75 | 'data extraction':ab | | 15045 |
| #76 | 'selection criteria':ab | | 24861 |
| #77 | #75 OR #76 | | 38411 |
| #78 | 'review'/exp OR review:it | | 2220273 |
| #79 | #77 AND #78 | | 18392 |
| #80 | letter:it | | 925412 |
| #81 | editorial:it | | 498965 |
| #82 | 'animal'/exp | | 21824906 |
| #83 | 'human'/exp | | 17149838 |
| #84 | #82 NOT (#82 AND #83) | | 4675068 |
| #85 | #80 OR #81 OR #84 | | 6067998 |
| #86 | #59 OR #68 OR #74 OR #79 | | 254976 |
| #87 | #86 NOT #85 | | 246140 |
| #88 | #37 AND #87 | | 298 |
| #89 | #55 OR #88 | | 3598 |

**Search Strategy Cochrane Library**

| Databases | | Cochrane Library (incl. Cochrane CENTRAL, Cochrane Reviews, DARE, NHSEED, HTA) | |
| --- | --- | --- | --- |
| Search Platform: | | Cochrane Library (Wiley), all databases included, see below | |
| Date of Search: | | June 3, 2016 [Last Database Update: Cochrane Central Register of Controlled Trials: May 2016, Cochrane Reviews: June 2016, Other Reviews (DARE): April 2015, HTA Database: April 2016, NHSEED: April 2015) | |
| Date Range Searched: | | No restriction | |
| Search Filters Used | | none | |
| # | **Search Terms** | | **Hits** |
| #1 | [mh ^"Carcinoma, Renal Cell"] | | 545 |
| #2 | [mh ^"kidney neoplasms"] | | 719 |
| #3 | (renal near/3 (carcinoma* or adenocarcinoma* or cancer* or neoplasm* or tumor* or tumour*)):ab,ti,kw | | 1414 |
| #4 | (kidney near/3 (carcinoma* or adenocarcinoma* or cancer* or neoplasm* or tumor* or tumour*)):ab,ti,kw | | 1303 |
| #5 | mrcc:ab,ti,kw or rcc:ab,ti,kw or hypernephroma*:ab,ti,kw or (nephroid next/1 carcinoma*):ab,ti,kw or (hypernephroid next/1 carcinoma*):ab,ti,kw or (grawitz next/1 tumor*):ab,ti,kw or (grawitz next/1 tumour*):ab,ti,kw | | 551 |
| #6 | #1 or #2 or #3 or #4 or #5 | | 1861 |
| #7 | cabozantinib*:ab,ti,kw | | 46 |
| #8 | ("bms 907351" or bms907351 or "xl 184" or xl184 or cabometyx* or cometriq*):ab,ti,kw | | 22 |
| #9 | [mh everolimus] | | 390 |
| #10 | everolimus*:ab,ti,kw | | 1425 |
| #11 | ("rad 001" or rad001 or "sdz rad" or afinitor* or certican* or zortress* or votubia*):ab,ti,kw | | 110 |
| #12 | axitinib*:ab,ti,kw | | 91 |
| #13 | ("ag 013736" or ag013736 or inlyta*):ab,ti,kw | | 16 |
| #14 | nivolumab*:ab,ti,kw | | 67 |
| #15 | ("mdx-1106" or mdx1106 or "ono-4538" or ono4538 or "bms-936558" or bms936558 or opdivo*):ab,ti,kw | | 17 |
| #16 | sorafenib:ab,ti,kw | | 562 |
| #17 | ("bay 43-9006" or bay 439006 or "bay43 9006" or bay439006 or "bay 545-9085" or "bay 5459085" or "bay545-9085" or bay5459085 or "bay 54-9085" or "bay 549085" or "bay54-9085" or bay549085 or nexavar*):ab,ti,kw | | 29 |
| #18 | Sunitinib*:ab,ti,kw | | 410 |
| #19 | (sutent* or "pha-290940ad" or pha290940ad or "su 011248" or su011248 or "su 11248" or su11248 or su010398 or "su 010398" or "su 10398" or su10398):ab,ti,kw | | 19 |
| #20 | Lenvatinib*:ab,ti,kw | | 31 |
| #21 | ("E7080" or "E 7080" or "ER-203492-00" or lenvima*) ab,ti,kw | | 0 |
| #22 | [mh ^"Protein Kinase Inhibitors"] | | 604 |
| #23 | "protein kinase inhibitor":kw or "protein serine threonine kinase inhibitor":kw or "protein tyrosine kinase inhibitor":kw | | 245 |
| #24 | #7 or #8 or #9 or #10 or #11 or #12 or #13 or #14 or #15 or #16 or #17 or #18 or #19 or #20 or #21 or #22 or #23 | | 3126 |
| #25 | #6 and #24 | | 458 |
| #26 | #6 and #24 in Cochrane Reviews (Reviews and Protocols), Other Reviews, Trials, Technology Assessments and Economic Evaluations | | 458 |

**Considerations concerning the choice of search filters in the identification of RCT and systematic reviews, meta-analyses**

NICE STA User guide^[[1]](#footnote-2)^ for company evidence submission recommends CRD Guidance^[[2]](#footnote-3)^ as source of information concerning the performance of systematic reviews.

In respect of search filters the CRD guidance mainly refers to two sources. One is the Hedges Project^[[3]](#footnote-4)^, the other one is the InterTASC Information Specialists’ Sub-Group (ISSG) Search Filter Resource^[[4]](#footnote-5)^. For projects intending to identify all papers on a topic filters with high sensitivity are recommended.

In the following table the chosen search filters and the reasons for the choice are listed:

| **Randomized Controlled Trials** | | | |
| --- | --- | --- | --- |
|  | **Medline** | **Embase** | **Cochrane Library** |
| Filter chosen | Cochrane Highly Sensitive Search Strategy for identifying randomized trials in MEDLINE: sensitivity-maximizing version (2008 revision)  *Source: Higgins JPT, Green S (editors). Cochrane Handbook for Systematic Reviews of Interventions Version 5.1.0 [updated March 2011]. The Cochrane Collaboration, 2011. Available from:* [*www.cochrane-handbook.org*](file:///\\la-ser.lan\DFS\LORRACH\Projects\_Active%20Projects\Ipsen\Cabozantinib%20EU%20MA%202016\06%20Systematic%20Literature%20Review\02%20Clinical%20Evidence\01%20Search%20Strategy\www.cochrane-handbook.org)*.* | SIGN Search Strategy Randomized Controlled Trials  *Source: Scottish Intermediate Guidelines Network (SIGN), Filter Randomized Controlled Trials*  *URL:* [*http://www.sign.ac.uk/methodology/filters.html#random*](http://www.sign.ac.uk/methodology/filters.html%23random) *[cited 20160512]* | No filter needed |
| Number of findings in the proposed search using this filter | 2539 findings | 3482 findings | 458 findings as a whole (RCT + Systematic Reviews, Meta-Analyses, HTA), same as below |
| Reasons for choice | - The Cochrane RCT-filter with High Sensitivity is listed on the ISSG Search Filter Resource. - NICE recommends it in the Guidelines Manual for use in the development of their guidelines^[[5]](#footnote-6)^ - With the use of the sensitivity-maximizing filter the findings to expect are still somehow manageable | - The SIGN search strategy is listed on the ISSG Search Filter Resource. - In the Axitinib submission it was used too and the search strategy was accepted. - An alternative would have been the filter from the Hedges Project.   However using the most sensitive filter would have resulted in a lot more findings.  Also there would have been the possibility to use the Hedges filter with best balance of sensitivity and specificity. That would have resulted in a manageable amount However as there is a more sensitive filter it is not sure how accepted this will be. | - |
| **Systematic Reviews** | | | |
|  | **Medline** | **Embase** | **Cochrane Library** |
| Filter chosen | SIGN Search Strategy Systematic Reviews  *Source: Scottish Intermediate Guidelines Network (SIGN), Filter Systematic Reviews*  *URL:* [*http://www.sign.ac.uk/methodology/filters.html#systematic*](http://www.sign.ac.uk/methodology/filters.html%23systematic), [Website l*ast modified 27/08/15]* | SIGN Search Strategy Systematic Reviews  *Source: Scottish Intermediate Guidelines Network (SIGN), Filter Systematic Reviews URL:* [*http://www.sign.ac.uk/methodology/filters.html#systematic*](http://www.sign.ac.uk/methodology/filters.html%23systematic), [Website l*ast modified 27/08/15]* | No filter needed |
| Number of findings in the proposed search using this filter | 107 findings | 298 findings | 458 findings as a whole (RCT + Systematic Reviews, Meta-Analyses, HTA), same as above |
| Reasons for choice | - The SIGN search strategy is listed on the ISSG Search Filter Resource. - The number of findings is manageable | - The SIGN search strategy is listed on the ISSG Search Filter Resource. - The number of findings is manageable | - |

1. Quality Assessment of Included Trials

Table 1 Critical appraisal of study quality

| **Author and year [author_year]** | **Study acronym or NCT number** | **Was randomisation carried out appropriately?** | | **Was the concealment of treatment allocation adequate?** | | **Were the groups similar at the outset of the study in terms of prognostic factors?** | | **Were the care providers, participants and outcome assessors blind to treatment allocation?** | | **Were there any unexpected imbalances in drop-outs between groups?** | | **Is there any evidence to suggest that the authors measured more outcomes than they reported?** | | **Did the analysis include an intention-to-treat analysis? If so, was this appropriate and were appropriate methods used to account for missing data?** | |
| --- | --- | --- | --- | --- | --- | --- | --- | --- | --- | --- | --- | --- | --- | --- | --- |
|  |  |  | **Justification** |  | **Justification** |  | **Justification** |  | **Justification** |  | **Justification** |  | **Justification** |  | **Justification** |
| Choueiri_2016; Choueiri_2015; Clinical Study Report | METEOR; NCT01865747 | Yes | 1:1 allocation, block size unknown; stratification by number of prior VEGFR-targeting TKI therapies (1 vs. 2 or more), number of risk factors per MSKCC prognostic criteria for previously treated patients with RCC (0 vs. 1 vs. 2 or 3). The study site used an interactive voice record system (IVRS) or interactive Web record system (IWRS) for randomization. | Yes | The study site used an interactive voice record system (IVRS) or interactive Web record system (IWRS) for randomization. | Yes | Demographic and baseline characteristics were balanced between the cabozantinib and everolimus groups. | No | Patients and investigators were not blinded to study treatment. A masked independent radiology comittee assessed progression-free survival, overall survival, tumor response, duration of response, and changes on bone scans. | No | There were no unexpected imbalances in drop-outs between groups for efficacy or safety analyses. | No | There is no evidence suggesting that the authors measured more outcomes than have been reported. | Yes | Efficacy was assessed in the intent-to-treat population. Safety analysis was conducted using a safety set (all patients receiving at least study drug once). |
| Escudier_2007 | TARGET; NCT00073307 | Not clear | 1:1 allocation with block size of four; stratification of patients by country and MSKCC prognostic score (low, intermediate); generation of randomization sequence unclear. | Not clear | No information | Yes | Demographic and baseline characteristics were balanced between the sorafenib and placebo groups. | Yes | It was a double-blind study. Patients and investigators were masked to study treatment. A blinded independent data and safety monitoring committee assessed outcomes. | No | There were no unexpected imbalances in drop-outs between groups for efficacy or safety analyses. | No | There is no evidence suggesting that the authors measured more outcomes than have been reported. | Yes | All randomized patients were included in the intent-to-treat population for efficacy analysis. Safety analysis was conducted using a safety set (all patients receiving at least study drug once). |
| Motzer_2015 | CheckMate 025; NCT01668784 | Not clear | 1:1 allocation with block size of four; stratification of patients according to region (United States or Canada, Western Europe, and the rest of the world), MSKCC prognostic risk group, and the number of previous antiangiogenig therapy regimes (one or two) for advanced renal cell carcinoma. Generation of randomization sequence unclear. | Not clear | No information | Yes | Demographic and baseline characteristics were balanced between the nivolumab and everolimus groups. | No | It was an open-label study. Patients and investigators were not blinded to study treatment. | No | There were no unexpected imbalances in drop-outs between groups for efficacy or safety analyses. | No | There is no evidence suggesting that the authors measured more outcomes than have been reported. | Yes | All randomized patients were included in the intent-to-treat population for efficacy analysis.Safety analysis was conducted using a safety set (all patients receiving at least study drug once). |
| Jonasch_2013 | NCT01239342 | Not clear | 2:1 allocation, no further information. Generation of randomization sequence unclear. | Not clear | No information | Not clear | No information | Not clear | No information | Not clear | No information | Not clear | No information | Not clear | No information |
| Motzer_2014 | GOLD;  NCT01223027 | Yes | 1:1 allocation; stratification by MSKCC prognostic score and region. The randomisation list for the patients was produced by the provider of the interactive web-based and voice response system using a validated system that automated the random assignment of patient numbers to randomisation numbers, which were linked to the two treatment groups. The randomisation scheme was reviewed and approved by the Novartis Randomization Offi ce (East Hanover, NJ, USA). | Yes | The study site used an interactive voice record system (IVRS) or interactive Web record system (IWRS) for randomization. | Yes | Demographic and baseline characteristics were balanced between the dovitinib and sorafenib groups. | No | It was an open-label study. Patients and investigators were not blinded to study treatment. A masked central review committee assessed progression-free survival. | No | There were no unexpected imbalances in drop-outs between groups for efficacy or safety analyses. | No | There is no evidence suggesting that the authors measured more outcomes than have been reported. | Yes | Efficacy was assessed in the intent-to-treat population. Safety analysis was conducted using a safety set (all patients receiving at least study drug once). |
| Motzer_2013 | TIVO-1; NCT01030783 | Not clear | 1:1 allocation; random assignment of patients was stratified by geographic region, number of prior treatments for metastatic disease, and number of metastatic sites/organs involved. Generation of randomization sequence unclear. | Not clear | No information | No | Demographic and baseline characteristics were balanced between study arms with the exception of ECOG performance status (applies to overall population). | No | It was an open-label study. Patients and investigators were not blinded to study treatment. A masked independent radiology comittee assessed progression-free survival and tumor response | No | There were no unexpected imbalances in drop-outs between groups for efficacy or safety analyses. | No | There is no evidence suggesting that the authors measured more outcomes than have been reported. | Yes | Efficacy was assessed in the intent-to-treat population. Safety analysis was conducted using a safety set (all patients receiving at least study drug once). |
| Motzer_2015 | NCT01136733 | Yes | Patients were randomly allocated in a 1:1:1 ratio to receive either lenvatinib plus everolimus, single-agent lenvatinib, or single-agent everolimus. An external interactive voice response system vendor (Parexel Informatics, NJ, USA) did randomisation centrally using a Pocock and Simon dynamic balancing procedure. | Not clear | Treatment concealment method was not addressed. | Yes | Baseline characteristics were well balanced between study groups. | No | It was an open-label study. Patients or investigators were not masked to study treatment. The funder was unaware of the aggregated by-treatment data summary until database lock. | No | There were no unexpected imbalances in drop-outs between groups for efficacy or safety analyses. | No | There is no evidence suggesting that the authors measured more outcomes than have been reported. | Yes | Efficacy was assessed in the intention-to-treat population. Safety was assessed in patients who received at least one dose of study drug. |
| Motzer_2013; Rini_2011 | AXIS; NCT00678392 | Yes | Patients were stratified according to ECOG status (0 or 1) and type of previous treatment (i.e., regimen containing sunitinib, bevacizumab, temsirolimus, or cytokine), and then randomly assigned them (1:1) to receive either axitinib or sorafenib. Randomisation lists were generated from an independent randomisation group using a permuted block design of size four (two to axitinib and two to sorafenib) within each stratum. | Yes | A web-enabled centralised registration system concealed treatment allocation before registration and allowed centres to enrol patients directly. Patients and investigators were not masked to study treatment. | Yes | Demographic and baseline characetristics were typical of a population with advanced RCC and were well balanced between the axitinib and sorafenib arm. | No | It was an open-label study. Patients and investigators were not masked to study treatment. Progression-free survival and objective response rate were assessed by a masked independent radiology review. | No | In the axitinib arm, 318/361 discontinued treatment (240 patients due to disease progression/relapse) and in sorafenib arm, 325/362 patients discontinued treatment (226 patients due to disease progression/relapse). There were no imbalances for drop-outs between groups for efficacy and safety analyses. | No | There is no evidence suggesting that the authors measured more outcomes than have been reported. | Yes | Efficacy was assessed in the intention-to-treat population. Safety was assessed in patients who received at least one dose of study drug. |
| Motzer_2008, Motzer_2010 | RECORD 1; NCT00410124 | Not clear | Patients were stratified according to a Memorial Sloan-Kettering Cancer Center (MSKCC) prognostic score (favourable vs intermediate vs poor risk) and previous anticancer therapy (one vs two previous VEGF receptor tyrosine kinase inhibitors). Patients were randomly assigned in a two to one ratio to everolimus or placebo with the use of permuted blocks of six (four to everolimus, two to placebo) within each stratum. Generation of randomization sequence unclear. | Not clear | Treatment concealment method was not addressed. | Yes | Baseline characteristics were well balanced between study groups. | Yes | Patient and investigator were blinded. Outcome analyses by independent review committee and by investigator review | No | There were no unexpected imbalances in drop-outs between groups for efficacy or safety analyses. | No | There is no evidence suggesting that the authors measured more outcomes than have been reported. | Yes | All randomly assigned patients were assessable for efficacy (intention-to-treat analysis). All patients receiving at least one dose of everolimus were eligible for safety analysis. Patients without tumour progression or death at the time of the data cutoff for the analysis or at the time of receiving an additional anticancer therapy were censored at their last date of adequate tumour evaluation. |
| Powles_2016 | ZEBRA; NCT01793636 | Yes | 1:1 allocation; Stratified randomisation was done with Cenduit software (Cenduit, Durham, NC, USA) using a password-protected computer database. Stratification factors included Memorial Sloan Kettering Cancer Centre (MSKCC) risk categories (favourable, intermediate, and poor) and PFS on initial targeted therapy (>6 vs <6 mo). | Yes | Randomisation was done with Cenduit software (Cenduit, Durham, NC, USA) using a password-protected computer database | No | Imbalances were observed in key factors such as median number of prior systemic treatments, and in MKSCC intermediate and poor risk scores. | No | It was an open-label study. Patients and investigators were not blinded to study treatment. | No | The trial was terminated early. In the AZD2014 arm, 21/26 patients discontinued treatment (15 patients due to disease progression) and in everolimus arm, 19/23 patients discontinued treatment (11 patients due to disease progression). Small number of participants may lead to imbalances in the final analyses. | No | There is no evidence suggesting that the authors measured more outcomes than have been reported. | Yes | Trial was terminated early after enrolment of 49 patients. All patients randomized were analysed for efficacy and safety outcomes. |
| Powles_2016 | ROVER; NCT01442090 | Yes | Patients were randomized 1:1 by using a dynamic hierarchical randomization algorithm through an interactive voice response system. Patients were stratified according to Memorial Sloan Kettering Cancer Center (MSKCC) prognostic score (favorable, intermediate, poor) and time to progression after starting their first VEGFR-targeted therapy (progressive disease [PD] <= 6 months or >6 months). | Yes | The study site used an interactive voice record system (IVRS) or interactive Web record system (IWRS) for randomization. | No | Imbalances were observed in key factors such as median number of prior systemic treatments, number of prior VEGF taregeted treatments received, and in number of target lesions. | No | It was an open-label study. Patients and investigators were not blinded to study treatment. | No | There were no unexpected imbalances in drop-outs between groups for efficacy or safety analyses. | No | There is no evidence suggesting that the authors measured more outcomes than have been reported. | Yes | All randomized patients were included for efficacy and safety analysis. |
| Guo_2015 | NCT02330783 | Not clear | 1:1 allocation, no further information. Generation of randomization sequence unclear. | Not clear | No information | Not clear | No information | Not clear | No information | Not clear | No information | Not clear | No information | Not clear | No information |
| Tannir_2016 | ESPN trial; NCT01185366 | Not clear | 1:1 allocation; Stratfication by MSKCC risk group and histologic RCC subtype (papillary vs, other). No further information. | Not clear | Treatment concealment method was not addressed. | No | Imbalances concerning gender and ECOG performance status. | No | Due to cross-over design, no blinding of patients and investigators. A masked independent radiological committee assessed tumor response. | No | There were no unexpected imbalances in drop-outs between groups for efficacy or safety analyses. | No | There is no evidence suggesting that the authors measured more outcomes than have been reported. | Yes | All randomized patients were included for efficacy and safety analysis. |
| Motzer_2014 | RECORD-3 | Not clear | 1:1 allocation; Stratfication by MSKCC risk group . No further information. | Not clear | Treatment concealment method was not addressed. | No | Characteristics were balanced between treatment arms in the first-line setting, except for Karnofsky performance status, which favored sunitinib. Characteristics also were balanced between arms in the second-line setting | No | It was an open-label study. Patients and investigators were not blinded to study treatment. | No | There were no unexpected imbalances in drop-outs between groups for efficacy or safety analyses. | No | There is no evidence suggesting that the authors measured more outcomes than have been reported. | Yes | All randomized patients were included for efficacy analysis. The safety population excluded two patients that never received study drug. |
| Eichelberg_2015 | SWITCH | Yes | Patients were randomised to sorafenib 400 mg twice daily followed by sunitinib 50 mg once daily (4 wk on, 2 wk off) (So Su) or vice versa (Su-So). Randomisation was stratified by MSKCC score (favourable vs intermediate). No further information | Yes | Centralised randomisation via fax coordinated by external company. The randomisation list was generated by an external company using an SAS program | Yes | Patient demographics and baseline characteristics were well balanced between the treatment groups | No | It was an open-label study. Patients and investigators were not blinded to study treatment. | No | There were no unexpected imbalances in drop-outs between groups for efficacy or safety analyses. | No | There is no evidence suggesting that the authors measured more outcomes than have been reported. | Yes | Efficacy was assessed in the intent-to-treat population. Safety analysis was conducted using a safety set (all patients receiving at least study drug once). |
| Pal_2015 | DisrupTOR-1 | Not clear | 1:1 allocation, no further information. Generation of randomization sequence unclear. | Not clear | Treatment concealment method was not addressed. | Yes | Patient demographics and baseline characteristics were well balanced between the treatment groups | Not Clear | No information | No | There were no unexpected imbalances in drop-outs between groups for efficacy or safety analyses. | No | There is no evidence suggesting that the authors measured more outcomes than have been reported. | Yes | All randomized patients were included for efficacy and safety analysis. |
| Hutson_2014 | INTORSECT | Not clear | Randomization was stratified according to baseline factors: prior nephrectomy (yes or no), duration of sunitinib therapy (<= or >180 days), tumor histology (clear or non–clear cell), and MSKCC (favorable, intermediate, or poor). Generation of randomization sequence unclear. | Yes | A computerized, centrally located randomization system was used to assign patient identification and treatment. | Yes | Patient demographics and baseline characteristics were well balanced between the treatment groups | No | It was an open-label study. Patients and investigators were not blinded to study treatment. | No | There were no unexpected imbalances in drop-outs between groups for efficacy or safety analyses. | No | There is no evidence suggesting that the authors measured more outcomes than have been reported. | Yes | Efficacy was assessed in the intent-to-treat population. Safety analysis was conducted using a safety set (all patients receiving at least study drug once). |
| Powles_2014 | ROVER NCT01442090 | Not clear | Conference abstract. No further information | Not clear | Treatment concealment method was not addressed. | Not clear | Conference abstract. No further information | Not clear | Conference abstract. No further information | Not clear | Conference abstract. No further information | Not clear | Conference abstract. No further information | Not clear | Conference abstract. No further information |
| Ratain_2006 | Ratain_2006 | Not clear | Generation of randomization sequence unclear. | Yes | Randomisation was done by centralized allocation via a telephone randomization system | Yes | In the randomized phase, the distribution ofmen and women differed between the treatment groups. However, there were no significant differences between groups for this or any of the other measured baseline characteristics. | Yes | Patients who had a change in tumor size of less than25%were randomly assigned to either sorafenib (at current dose) or matching placebo in a double-blinded fashion. In order to verify investigator observations in an unbiased manner, independent assessment of radiologic scans was performed retrospectively for 152 (75%) of 202 patients. | No | There were no unexpected imbalances in drop-outs between groups for efficacy or safety analyses. | No | There is no evidence suggesting that the authors measured more outcomes than have been reported. | Yes | Efficacy was assessed in the intent-to-treat population. Safety analysis was conducted using a safety set (all patients receiving at least study drug once). |

Table 2 Summary of critical appraisal of study quality assessment

1. User guide for company submission of evidence - 2015 version, <https://www.nice.org.uk/article/pmg24> [↑](#footnote-ref-2)
2. Systematic reviews: CRD's guidance for undertaking reviews in health care (University of York Centre for Reviews and Dissemination), <http://www.york.ac.uk/inst/crd/pdf/Systematic_Reviews.pdf> [↑](#footnote-ref-3)
3. Hedges Project, <http://hiru.mcmaster.ca/hiru/HIRU_Hedges_home.aspx> [↑](#footnote-ref-4)
4. InterTASC Information Specialists’ Sub-Group (ISSG) Search Filter Resource, <https://sites.google.com/a/york.ac.uk/issg-search-filters-resource/home> [↑](#footnote-ref-5)
5. NICE Guidelines Manual, <https://www.nice.org.uk/article/pmg6/chapter/5-identifying-the-evidence-literature-searching-and-evidence-submission> [↑](#footnote-ref-6)
