# Supplementary material for: Cabozantinib versus everolimus, nivolumab, axitinib, sorafenib and best supportive care: A network meta-analysis of progression-free survival and overall survival in second line treatment of advanced renal cell carcinoma
Source: PLoS One. 2017 Sep 8;12(9):e0184423. doi: 10.1371/journal.pone.0184423 (PMC5590935; doi:10.1371/journal.pone.0184423)
Supplement: S3 File — (DOCX) [file pone.0184423.s003.docx]

1. **Fixed-effects Model**

$\log\left( h_{jkt} \right)= \nu_{jk}+\theta_{jk}log(t)$ (1) $\binom{\nu_{jk}}{\theta_{jk}}=\binom{\nu_{jk}}{\theta_{jk}}=\left\{ \begin{aligned} \binom{\mu_{1jb}}{\mu_{2jb}}, \mathrm{if}k=b, b=\{everolimus,placebo,sorafenib\} (3 "baseline" treatments) \\ \binom{\mu_{1jb}}{\mu_{2jb}}+\binom{\delta_{1jbk}}{\delta_{2jbk}}, \mathrm{if}k\text{ }\text{is different from }\text{b}, b=\{everolimus,placebo,sorafenib\} \end{aligned} \right.$

$\mathrm{with} \binom{\delta_{1jbk}}{\delta_{2jbk}}=\binom{d_{1Ak}}{d_{2Ak}}-\binom{d_{1Ab}}{d_{2Ab}} for an indirect comparison$.

In this formula, $h_{jkt}$ represents the underlying hazard rate in study $j$ for treatment $k$ at time point $t$. The vector $\binom{\mu_{1jb}}{\mu_{2jb}}$ is treatment-specific and reflects the scale $\nu$ and shape $\theta$ of the “baseline” treatment$b$ in study $j$. The vector $\binom{\delta_{1jbk}}{\delta_{2jbk}}$ reflects the study-specific difference in parameters $\nu$ and $\theta$ of the effect for treatment$k$ relative to the “baseline” treatment $b$ in study $j$, where both treatments $k$ and $b$ are pooled with help of the reference treatment $A$. For example, cabozantinib (from METEOR) and nivolumab (from CheckMate025**)** could be compared indirectly through their reference treatment everolimus, as follows.

$$\binom{\delta_{1cabo,nivo}}{\delta_{2cabo,nivo}}=\binom{d_{1ever,nivo}}{d_{2ever,nivo}}-\binom{d_{1ever,cabo}}{d_{2ever,cabo}}$$

In our case study (see Figure 1 for the evidence network used in the NMA), everolimus was the “baseline” treatment in METEOR, CheckMate025, and RECORD-1; placebo was the “baseline” treatment in TARGET and sorafenib was the “baseline” treatment in AXIS. They also played the role of reference treatments to ensure indirect comparisons. For example, cabozantinib in METEOR could be compared indirectly with axitinib in AXIS through a combination of reference treatments of everolimus, placebo and sorafenib. Estimation of model parameters of interest – baseline and effect vectors – was performed in Bayesian framework. Without losing generality, the prior distributions as used for the parameters of the fixed-effects model were chosen non-informative, assuming that no prior information on the mean scale or location parameters or their correlation was provided (Ouwens et al. 2010 [17]). The prior variances $T_{\mu}$ and $T_{\mu}$ of the parameters were chosen to be large (but finite) to ensure the inferences were not sensitive to the choice of non-informative prior distribution.

$$\binom{\mu_{1jb}}{\mu_{2jb}} \sim N\left( \binom{0}{0}, T_{\mu} \right), T_{\mu}= \binom{{10}^{4} 0}{0 {10}^{4}}$$

$$\binom{d_{1Ak}}{d_{2Ak}} \sim N\left( \binom{0}{0}, T_{d} \right), T_{d}= \binom{{10}^{4} 0}{0 {10}^{4}}$$
