# Supplementary material for: Cabozantinib versus everolimus, nivolumab, axitinib, sorafenib and best supportive care: A network meta-analysis of progression-free survival and overall survival in second line treatment of advanced renal cell carcinoma
Source: PLoS One. 2017 Sep 8;12(9):e0184423. doi: 10.1371/journal.pone.0184423 (PMC5590935; doi:10.1371/journal.pone.0184423)
Supplement: S4 File — (DOCX) [file pone.0184423.s004.docx]

1. **Random-effects Model**

As presented in Ouwens et al. (2010) [17] the random-effects model was defined as follows:

$\log\left( h_{jkt} \right)= \nu_{jk}+\theta_{jk}log(t)$ (1)

$$\binom{\nu_{jk}}{\theta_{jk}}=\left\{ \begin{aligned} \binom{\mu_{1jb}}{\mu_{2jb}}, if k=b, b=\{everolimus,placebo,sorafenib\} (3 "baseline" treatments) \\ \binom{\mu_{1jb}}{\mu_{2jb}}+\binom{\delta_{1jbk}}{\delta_{2jbk}}, if k diffrent from b \end{aligned} \right.$$

$$\binom{\delta_{1jbk}}{\delta_{2jbk}}\sim N\left( \binom{d_{1Ak}}{d_{2Ak}}-\binom{d_{1Ab}}{d_{2Ab}}, \Sigma\right), \Sigma= \left( \begin{matrix} \sigma_{1}^{2} & \sigma_{1}\sigma_{2}\rho\\ \sigma_{1}\sigma_{2}\rho& \sigma_{2}^{2} \end{matrix} \right)$$

In this formula, $h_{jkt}$ represents the underlying hazard rate in study $j$ for treatment $k$ at time point $t$. The vectors $\binom{\mu_{1jb}}{\mu_{2jb}}$ are treatment-specific and reflect the parameters $\nu$ and $\theta$ of the “baseline” treatment in study $j$. In our case study, everolimus was the “baseline” treatment in METEOR, CheckMate025, and RECORD-1; placebo was the “baseline” treatment in TARGET and sorafenib was the “baseline” treatment in AXIS. The vector $\binom{\delta_{1jbk}}{\delta_{2jbk}}$ reflects the study-specific difference in scale $\nu$ and shape $\theta$ of the log-hazard curve for treatment$k$ relative to the “baseline” treatment in study $j$. In our case study, treatment $k$ corresponded to cabozantinib in METEOR, nivolumab in CheckMate025, placebo in RECORD-1, sorafenib in TARGET and axitinib in AXIS.

Estimation of model parameters of interest – baseline and effect vectors – was performed in Bayesian framework. The prior distributions as used for the parameters of the random-effects model were chosen non-informative as follows:

$$\binom{\mu_{1jb}}{\mu_{2jb}} \sim N\left( \binom{0}{0}, T_{\mu} \right), T_{\mu}= \binom{{10}^{4} 0}{0 {10}^{4}}$$

$$\binom{d_{1Ak}}{d_{2Ak}} \sim N\left( \binom{0}{0}, T_{d} \right), T_{d}= \binom{{10}^{4} 0}{0 {10}^{4}}$$

$$\Sigma\sim Wishart\left( \Omega, 2 \right), \Omega= \binom{{10}^{3} 0}{0 {10}^{3}}$$

$\binom{\mu_{1jb}}{\mu_{2jb}}$ was sampled from a non-informative bivariate normal distribution with 0 correlation. The same holded for the vector of treatment effect$\binom{d_{1Ak}}{d_{2Ak}}$. A Wishart prior distribution $Wishart\left( \Omega, 2 \right)$was specified for the variance matrix $\Sigma$, with $\Omega$ the scale matrix and 2 the smallest degrees of freedom. In our experiment, the values of $\Omega$ have been changed from $\binom{{10}^{2} 0}{0 {10}^{2}}$ to $\binom{{10}^{3} 0}{0 {10}^{3}}$ to accelerate the convergence rate. In fact, with the smaller variance, the algorithm did not converge with 100 000 iterations but we got a good convergence with 80 000 iterations once we increased the prior mean of $\Sigma(scale matrix \Omega)$. On the contrary, we did not meet any convergence issue with the fixed-effects model.
