# Supplementary material for: Cabozantinib versus everolimus, nivolumab, axitinib, sorafenib and best supportive care: A network meta-analysis of progression-free survival and overall survival in second line treatment of advanced renal cell carcinoma
Source: PLoS One. 2017 Sep 8;12(9):e0184423. doi: 10.1371/journal.pone.0184423 (PMC5590935; doi:10.1371/journal.pone.0184423)
Supplement: S5 File — (DOCX) [file pone.0184423.s005.docx]

### Algorithm estimation

1. **Programming Code for Parametric Survival Curve NMA**

#####################################################################

######### Bayesian network meta-analysis ###########

#####################################################################

#####################################################################

### PREPARING INPUT DATA AND INITIAL VALUES FOR WINBUGS

#####################################################################

library(R2WinBUGS)

library(ggplot2)

*### Computing inputs via Guyot-Ouwens algorithm*

ns_convert <- function(input, t, b, s) {

input$time <- c(input[-1,"V1"], 1000)

input$dt <- input$time - c(0, input[-nrow(input),"time"])

input$n <- input$V2 # number of patients at risk in the interval

input$r <- input$V3 # number of deaths in the interval

input$t <- t # treatment

input$b <- b # baseline treatment

input$s <- s # study

return(input[-nrow(input),c("time", "dt", "n", "r", "t", "b", "s")])

}

winbugsi_new <- rbind(ns_convert(read.table("METEOR_OS_cabozantinib_KMdata.txt", header=T), 2, 1, 1), ns_convert(read.table("METEOR_OS_everolimus_KMdata.txt", header=T), 1, 1, 1), ns_convert(read.table("checkmate_OS_nivolumab_KMdata.txt", header=T), 3, 1, 2), ns_convert(read.table("checkmate_OS_everolimus_KMdata.txt", header=T), 1, 1, 2), ns_convert(read.table("NCT_OS_lenvatinib_everolimus_KMdata.txt", header=T), 4, 1, 3), ns_convert(read.table("NCT_OS_everolimus_KMdata.txt", header=T), 1, 1, 3), ns_convert(read.table("RECORD2_OS_placebo_KMdata.txt", header=T), 5, 1, 4), ns_convert(read.table("RECORD2_OS_everolimus_KMdata.txt", header=T), 1, 1, 4), ns_convert(read.table("TARGET_OS_sorafenib_KMdata.txt", header=T), 6, 5, 5), ns_convert(read.table("TARGET_OS_placebo_KMdata.txt", header=T), 5, 5, 5), ns_convert(read.table("AXIS_OS_Sunitinib_based_axitinib_KMdata.txt", header=T),7, 6, 6), ns_convert(read.table("AXIS_OS_Sunitinib_based_sorafenib_KMdata.txt", header=T), 6, 6, 6))

winbugsi <- winbugsi_new[winbugsi_new$n > 0,]

*### Input data:*

bugs_input <- list("N"=nrow(winbugsi), "NS"=6, "NT"=7, "mean"=c(0,0), "prec2"=matrix(c(.0001,0,0,.0001),2,2), "R"=matrix(c(.01,0,0,.01),2,2), "time"=winbugsi[,1], "dt"=winbugsi[,2], "n"=winbugsi[,3], "r"=winbugsi[,4], "t"=winbugsi[,5], "b"=winbugsi[,6], "s"=winbugsi[,7], "ts"=c(2,3,4,5,6,7), "bs"=c(1,1,1,1,5,6))

bugs_input_fixed <- bugs_input

bugs_input_fixed$R <- NULL

### *Initial values:*

bugs_inits <- list(

list("mu"=matrix(-c(1,1,1,1,1,1,.1,.1,.1,.1,.1,.1),6,2), "d"=matrix(c(NA, 2, 2,-2, -2,-2,-2,NA,.1,.1, -.1, -.1, -.1, -.1),7,2)),

list("mu"=matrix(-c(1,1,1,1,1,1,.1,.1,.1,.1,.1,.1),6,2), "d"=matrix(c(NA,-2, 2,2,2,-2,2,NA, -.1,.1, .1, -.1, -.1, -.1),7,2)),

list("mu"=matrix( c(-1,-1,-1,-1,-1,-1,-.1,-.1,-.1,-.1,-.1,-.1),6,2), "d"=matrix(c(NA, 2, 2,2,2,2,2,NA,.1, .1, .1, -.1, -.1, -.1),7,2)),

list("mu"=matrix(-c(-1,-1,-1,-1,-1,-1,-.1,-.1,-.1,-.1,-.1,-.1),6,2), "d"=matrix(c(NA,-2,-2,-2,-2,-2,-2,NA,-.1,-.1,-.1, -.1, -.1, -.1),7,2))

)

#####################################################################

### WINBUGS MODELLING

#####################################################################

*# weibull*

weib.fixed <- bugs(data = bugs_input_fixed, inits=bugs_inits, "BUGS run", model.file="bugs_model_Weibull.txt", bugs.directory="C:/Users/janedoe/Documents/WinBUGS14", parameters=c("mu", "d"), n.chains=4, n.iter=50000, n.burnin=25000, n.thin=10)

*# gompertz*

gompertz.fixed <- bugs(data = bugs_input_fixed, inits=bugs_inits, "BUGS run", model.file="bugs_model_Gompertz.txt", bugs.directory="C:/Users/janedoe/Documents/WinBUGS14", parameters=c("mu", "d"), n.chains=4, n.iter=50000, n.burnin=25000, n.thin=10)

*# loglogistic*

log.fixed <- bugs(data = bugs_input_fixed, inits=bugs_inits, "BUGS run", model.file="bugs_model_loglogistic.txt", bugs.directory="C:/Users/janedoe/Documents/WinBUGS14", parameters=c("mu", "d"), n.chains=4, n.iter=50000, n.burnin=25000, n.thin=10, debug=FALSE)

*# lognormal*

lognorm.fixed <- bugs(data = bugs_input_fixed, inits=bugs_inits, "BUGS run", model.file="bugs_model_lognormal.txt", bugs.directory="C:/Users/janedoe/Documents/WinBUGS14", parameters=c("mu", "d"), n.chains=4, n.iter=50000, n.burnin=25000, n.thin=10, debug=FALSE)

*# exponential: needs other initial values as unidimensional*

bugs_inits_exp <- list(

list("mu"=c(1,1,1,1,1,1), "d"=c(NA, .1,.1, -.1, -.1, -.1, -.1)),

list("mu"=c(1,1,1,1,1,1), "d"=c(NA,.1,.1, -.1, -.1, -.1, -.1)),

list("mu"=c(-1,-1,-1,-1,-1,-1), "d"=c(NA, .1,.1, -.1, -.1, -.1, -.1)),

list("mu"=c(-1,-1,-1,-1,-1,-1), "d"=c(NA,.1,.1, -.1, -.1, -.1, -.1))

)

exp.fixed <- bugs(data = bugs_input_fixed, inits=bugs_inits_exp, "BUGS run", model.file="bugs_model_exponential.txt", bugs.directory="C:/Users/janedoe/Documents/WinBUGS14", parameters=c("mu", "d"), n.chains=4, n.iter=50000, n.burnin=25000, n.thin=10)

#####################################################################

### COMPUTING RESULTS

#####################################################################

*### Sample from posterior*

xsim <- seq(0,24, length=500) # 24 months

sm.weib <- weib.fixed$sims.matrix

sm.gomp <- gompertz.fixed$sims.matrix

sm.log <- log.fixed$sims.matrix

sm.exp <- exp.fixed$sims.matrix

sm.lognorm <- lognorm.fixed$sims.matrix

*### Estimated survival function with help of estimated parameters*

gomp.surv <- function(xsim, sm) {

mean.para <- apply(sm[,1:2],2,mean)

para <- rep(0, 2)

para[1] <- exp(mean.para[1])

para[2] <- mean.para[2]

res <- exp(-para[1]/para[2]*(exp(xsim*para[2]) - 1))

return(res)

}

weib.surv <- function(xsim, sm) {

mean.para <- apply(sm[,1:2],2,mean)

para <- rep(0, 2)

para[1] <- exp(mean.para[1])/(mean.para[2]+1)

para[2] <- mean.para[2] + 1

res <- exp(-((xsim^para[2])*para[1]))

return(res)

}

log.surv <- function(xsim, sm) {

mean.para <- apply(sm[,1:2],2,mean)

para <- rep(0, 2)

para[1] <- exp(-exp(mean.para[2])*mean.para[1])

para[2] <- exp(mean.para[2])

res <- 1/(1+para[1]*xsim^para[2])

return(res)

}

exp.surv <- function(xsim, sm) {

para <- exp(mean(sm))

res <- exp(-xsim*para)

return(res)

}

lognorm.surv <- function(xsim, sm) {

mean.para <- apply(sm[,1:2],2,mean)

para <- rep(0, 2)

para[1] <- mean.para[1]

para[2] <- exp(mean.para[2])

res <- pnorm(-(log(xsim)-para[1])/para[2])

return(res)

}

*###By choosing columns of sm matrix we change baseline / effect definitions, to adjust treatment to different studies*

*### weibull, survival with estimated parameter*

grweib1.esti <- weib.surv(xsim, sm.weib[,1:2]) #using parameters for everolimus in study 1

grweib7.esti <- weib.surv(xsim, sm.weib[,1:2]+sm.weib[,c(13,14)]) #using parameters for cabo in study 1

grweib8.adjusted.esti <- weib.surv(xsim, sm.weib[,1:2]+sm.weib[,c(15,16)]) #using parameters for nivo adjusted to study 1

grweib9.adjusted.esti <- weib.surv(xsim, sm.weib[,1:2]+sm.weib[,c(17,18)]) #using parameters for lenv+ever adjusted to study 1

grweib10.adjusted.esti <- weib.surv(xsim, sm.weib[,1:2]+sm.weib[,c(19,20)]) #using parameters for placebo adjusted to study 1

grweib11.adjusted.esti <- weib.surv(xsim, sm.weib[,1:2]+sm.weib[,c(21,22)]) #using parameters for sora adjusted to study 1

grweib12.adjusted.esti <- weib.surv(xsim, sm.weib[,1:2]+sm.weib[,c(23,24)]) #using parameters for axit adjusted to study 1

*### gompertz, survival with estimated parameter*

grgomp1.esti <- gomp.surv(xsim, sm.gomp[,1:2]) #using parameters for everolimus in study 1

grgomp7.esti <- gomp.surv(xsim, sm.gomp[,1:2]+sm.gomp[,c(13,14)]) #using parameters for cabo in study 1

grgomp8.adjusted.esti <- gomp.surv(xsim, sm.gomp[,1:2]+sm.gomp[,c(15,16)]) #using parameters for nivo adjusted to study 1

grgomp9.adjusted.esti <- gomp.surv(xsim, sm.gomp[,1:2]+sm.gomp[,c(17,18)]) #using parameters for lenv+ever adjusted to study 1

grgomp10.adjusted.esti <- gomp.surv(xsim, sm.gomp[,1:2]+sm.gomp[,c(19,20)]) #using parameters for placebo adjusted to study 1

grgomp11.adjusted.esti <- gomp.surv(xsim, sm.gomp[,1:2]+sm.gomp[,c(21,22)]) #using parameters for sora adjusted to study 1

grgomp12.adjusted.esti <- gomp.surv(xsim, sm.gomp[,1:2]+sm.gomp[,c(23,24)]) #using parameters for axit adjusted to study 1

*### log-logistic, survival with estimated parameter:*

grlog1.esti <- log.surv(xsim, sm.log[,1:2]) #using parameters for everolimus in study 1

grlog7.esti <- log.surv(xsim, sm.log[,1:2]+sm.log[,c(13,14)]) #using parameters for cabo in study 1

grlog8.adjusted.esti <- log.surv(xsim, sm.log[,1:2]+sm.log[,c(15,16)]) #using parameters for nivo adjusted to study 1

grlog9.adjusted.esti <- log.surv(xsim, sm.log[,1:2]+sm.log[,c(17,18)]) #using parameters for lenv+ever adjusted to study 1

grlog10.adjusted.esti <- log.surv(xsim, sm.log[,1:2]+sm.log[,c(19,20)]) #using parameters for placebo adjusted to study 1

grlog11.adjusted.esti <- log.surv(xsim, sm.log[,1:2]+sm.log[,c(21,22)]) #using parameters for sora adjusted to study 1

grlog12.adjusted.esti <- log.surv(xsim, sm.log[,1:2]+sm.log[,c(23,24)]) #using parameters for axit adjusted to study 1

*### exponential, survival with estimated parameter:*

grexp1.esti <- exp.surv(xsim, sm.exp[,1]) #using parameters for everolimus in study 1

grexp7.esti <- exp.surv(xsim, sm.exp[,1]+sm.exp[,c(7)]) #using parameters for cabo in study 1

grexp8.adjusted.esti <- exp.surv(xsim, sm.exp[,1]+sm.exp[,c(8)]) #using parameters for nivo adjusted to study 1

grexp9.adjusted.esti <- exp.surv(xsim, sm.exp[,1]+sm.exp[,c(9)]) #using parameters for lenv+ever adjusted to study 1

grexp10.adjusted.esti <- exp.surv(xsim, sm.exp[,1]+sm.exp[,c(10)]) #using parameters for placebo adjusted to study 1

grexp11.adjusted.esti <- exp.surv(xsim, sm.exp[,1]+sm.exp[,c(11)]) #using parameters for sora adjusted to study 1

grexp12.adjusted.esti <- exp.surv(xsim, sm.exp[,1]+sm.exp[,c(12)])#using parameters for axit adjusted to study 1

*### lognormal, survival with estimated parameter*

grlognorm1.esti <- lognorm.surv(xsim, sm.lognorm[,1:2]) #using parameters for everolimus in study 1

grlognorm7.esti <- lognorm.surv(xsim, sm.lognorm[,1:2]+sm.lognorm[,c(13,14)]) #using parameters for cabo in study 1

grlognorm8.adjusted.esti <- lognorm.surv(xsim, sm.lognorm[,1:2]+sm.lognorm[,c(15,16)]) #using parameters for nivo adjusted to study 1

grlognorm9.adjusted.esti <- lognorm.surv(xsim, sm.lognorm[,1:2]+sm.lognorm[,c(17,18)]) #using parameters for lenv+ever adjusted to study 1

grlognorm10.adjusted.esti <- lognorm.surv(xsim, sm.lognorm[,1:2]+sm.lognorm[,c(19,20)]) #using parameters for placebo adjusted to study 1

grlognorm11.adjusted.esti <- lognorm.surv(xsim, sm.lognorm[,1:2]+sm.lognorm[,c(21,22)]) #using parameters for sora adjusted to study 1

grlognorm12.adjusted.esti <- lognorm.surv(xsim, sm.lognorm[,1:2]+sm.lognorm[,c(23,24)]) #using parameters for axit adjusted to study 1

#####################################################################

### WINBUGS MODELS

#####################################################################

*### Weibull*

Model{

for (i in 1:N){ # N=number of data points in dataset

#likelihood

r[i]~ dbin(p[i],n[i])

p[i]<-1-exp(-h[i]*dt[i])

log(h[i])<-nu[i] + log(time[i])*theta[i]

nu[i]<-mu[s[i],1]+delta[s[i],1]*(1-equals(t[i],b[i]))

theta[i]<-mu[s[i],2]+ delta[s[i],2]*(1-equals(t[i],b[i]))

}

for(k in 1 :NS){ # NS=number of studies in dataset

delta[k,1] <- md[k,1]

delta[k,2] <- md[k,2]

md[k,1]<-d[ts[k],1]-d[bs[k],1]

md[k,2]<-d[ts[k],2]-d[bs[k],2]

}

# priors

d[1,1]<-0

d[1,2]<-0

for(j in 2 :NT){ # NT=number of treatments

d[j,1:2] ~ dmnorm(mean[1:2],prec2[,])

}

for(k in 1 :NS){

mu[k,1:2] ~ dmnorm(mean[1:2],prec2[,])

}

}

*### Gompertz*

Model{

for (i in 1:N){ # N=number of data points in dataset

#likelihood

r[i]~ dbin(p[i],n[i])

p[i]<-1-exp(-h[i]*dt[i])

log(h[i])<-nu[i] + (time[i]*theta[i])

nu[i]<-mu[s[i],1]+delta[s[i],1]*(1-equals(t[i],b[i]))

theta[i]<-mu[s[i],2]+ delta[s[i],2]*(1-equals(t[i],b[i]))

}

for(k in 1 :NS){ # NS=number of studies in dataset

delta[k,1] <- md[k,1]

delta[k,2] <- md[k,2]

md[k,1]<-d[ts[k],1]-d[bs[k],1]

md[k,2]<-d[ts[k],2]-d[bs[k],2]

}

# priors

d[1,1]<-0

d[1,2]<-0

for(j in 2 :NT){ # NT=number of treatments

d[j,1:2] ~ dmnorm(mean[1:2],prec2[,])

}

for(k in 1 :NS){

mu[k,1:2] ~ dmnorm(mean[1:2],prec2[,])

}

}

*### Log-logistic*

Model{

for (i in 1:N){ # N=number of data points in dataset

#likelihood

r[i]~ dbin(p[i],n[i])

p[i]<-1-exp(-h[i]*dt[i])

h[i]<-(exp(theta[i])/exp(nu[i]))*pow(time[i]/exp(nu[i]), exp(theta[i])-1)/(1+pow(time[i]/exp(nu[i]), exp(theta[i])))

nu[i] <- mu[s[i],1] + (delta[s[i],1]*(1-equals(t[i],b[i])))

#log(nu[i])<-mu[s[i],1]+delta[s[i],1]*(1-equals(t[i],b[i]))

theta[i] <- mu[s[i],2]+ (delta[s[i],2]*(1-equals(t[i],b[i])))

#log(theta[i])<-mu[s[i],2]+ delta[s[i],2]*(1-equals(t[i],b[i]))

}

for(k in 1 :NS){ # NS=number of studies in dataset

delta[k,1] <- md[k,1]

delta[k,2] <- md[k,2]

md[k,1]<-d[ts[k],1]-d[bs[k],1]

md[k,2]<-d[ts[k],2]-d[bs[k],2]

}

# priors

d[1,1]<-0

d[1,2]<-0

for(j in 2 :NT){ # NT=number of treatments

d[j,1:2] ~ dmnorm(mean[1:2],prec2[,])

}

for(k in 1 :NS){

mu[k,1:2] ~ dmnorm(mean[1:2],prec2[,])

}

}

*### Log-normal*

Model{

for (i in 1:N){ # N=number of data points in dataset

#likelihood

r[i]~ dbin(p[i],n[i])

p[i]<-1-exp(-h[i]*dt[i])

#h[i]<-pow(2*3.1415926,-0.5)*exp(-pow(-(log(time[i]-nu[i]))/theta[i],2)*0.5)/(theta[i]*time[i]*phi(-(log(time[i]-nu[i]))/theta[i]))

h[i]<-pow(2*3.1415926,-0.5)*exp(-pow((log(time[i])-nu[i])/exp(theta[i]),2)*0.5) / (exp(theta[i])*time[i]*phi(-(log(time[i])-nu[i])/exp(theta[i])))

nu[i] <- mu[s[i],1] + (delta[s[i],1]*(1-equals(t[i],b[i])))

theta[i] <- mu[s[i],2]+ (delta[s[i],2]*(1-equals(t[i],b[i])))

}

for(k in 1 :NS){ # NS=number of studies in dataset

delta[k,1] <- md[k,1]

delta[k,2] <- md[k,2]

md[k,1]<-d[ts[k],1]-d[bs[k],1]

md[k,2]<-d[ts[k],2]-d[bs[k],2]

}

# priors

d[1,1]<-0

d[1,2]<-0

for(j in 2 :NT){ # NT=number of treatments

d[j,1:2] ~ dmnorm(mean[1:2],prec2[,])

}

for(k in 1 :NS){

mu[k,1:2] ~ dmnorm(mean[1:2],prec2[,])

}

}

*### Exponential*

Model{

for (i in 1:N){ # N=number of data points in dataset

#likelihood

r[i]~ dbin(p[i],n[i])

p[i]<-1-exp(-h[i]*dt[i])

log(h[i])<-nu[i] + log(time[i])*theta[i]

nu[i]<-mu[s[i]]+delta[s[i]]*(1-equals(t[i],b[i]))

theta[i]<-0

}

for(k in 1 :NS){ # NS=number of studies in dataset

delta[k] <- md[k]

md[k]<-d[ts[k]]-d[bs[k]]

}

# priors

d[1]<-0

for(j in 2 :NT){ # NT=number of treatments

d[j] ~ dnorm(mean[1],prec2[1,1])

}

for(k in 1 :NS){

mu[k] ~ dnorm(mean[1],prec2[1,1])

}

}
