# Supplementary material for: Cabozantinib versus everolimus, nivolumab, axitinib, sorafenib and best supportive care: A network meta-analysis of progression-free survival and overall survival in second line treatment of advanced renal cell carcinoma
Source: PLoS One. 2017 Sep 8;12(9):e0184423. doi: 10.1371/journal.pone.0184423 (PMC5590935; doi:10.1371/journal.pone.0184423)
Supplement: S6 File — (DOCX) [file pone.0184423.s006.docx]

### Transitivity Property

1. **Description of the transitivity property for each survival distribution**

Weibull distribution. The hazard function is

$$h(t)=\lambdaϒt^{ϒ-1}$$

This can be written as

$\log\left( h\left( t \right) \right)=\nu+ \theta\log\left( t \right)$ , with $\nu=log \left( \lambdaϒ \right), \theta=\left( ϒ-1 \right)$.

Transitivity of log HR at time t can be proven as

$\log\left( \psi_{BC}\left( t \right) \right)= \nu_{C}-\nu_{B}+{(\theta}_{C}-\theta_{B}$)$\log\left( t \right)$

$=\nu_{C}-\nu_{A}+{(\theta}_{C}-\theta_{A}$)$\log\left( t \right)-(\nu_{B}-\nu_{A}+{(\theta}_{B}-\theta_{A}) \log\left( t \right))$

$$=log \left( \psi_{AC}\left( t \right) \right)- \log\left( \psi_{AB}\left( t \right) \right)$$

Exponential distribution. The hazard function is

$$h(t)=\lambda$$

This can be written as

$\log\left( h\left( t \right) \right)=\nu$ , with $\nu=log \left( \lambda\right)$

Transitivity of log HR at time t can be proven as

$$\log\left( \psi_{BC}\left( t \right) \right)= \nu_{C}-\nu_{B}=\nu_{C}-\nu_{A}-{(\nu}_{B}-\nu_{A})$$

$$=log \left( \psi_{AC}\left( t \right) \right)- \log\left( \psi_{AB}\left( t \right) \right)$$

Gompertz distribution. The hazard function is

$$h\left( t \right)=ae^{bt}$$

This can be written as

$\log\left( h\left( t \right) \right)=\nu+ \theta t$ , with $\nu=log \left( a \right), \theta=b$.

Transitivity of log HR at time t can be proven as

$\log\left( \psi_{BC}\left( t \right) \right)= \nu_{C}-\nu_{B}+{(\theta}_{C}-\theta_{B}$) $t$

$=\nu_{C}-\nu_{A}+{(\theta}_{C}-\theta_{A}$)$t -(\nu_{B}-\nu_{A}+{(\theta}_{B}-\theta_{A}) t)$

$$=log \left( \psi_{AC}\left( t \right) \right)- \log\left( \psi_{AB}\left( t \right) \right)$$

Log-logistic distribution. The failure odds in a log-logistic model (Poyston and Parmar, 2002) is

$$\frac{1-S(t)}{S(t)}=\lambda t^{\theta}$$

This can be written as

$\log\left( \frac{1-S(t)}{S(t)} \right)=\nu+ \theta log(t)$ , with $\nu=log \left( \lambda\right).$

Transitivity of log-odds ratio at time t can be proven as

$\log\left( {OR}_{BC}\left( t \right) \right)= \nu_{C}-\nu_{B}+{(\theta}_{C}-\theta_{B}$) $log(t)$

$=\nu_{C}-\nu_{A}+{(\theta}_{C}-\theta_{A}$)$log(t) -(\nu_{B}-\nu_{A}+{(\theta}_{B}-\theta_{A}) log(t))$

$$=log \left( {OR}_{AC}\left( t \right) \right)- \log\left( {OR}_{AB}\left( t \right) \right)$$

Log-normal distribution. The survival function is

$S\left( t \right)= \Phi(-\frac{\log\left( t \right)-\alpha}{\beta})$, with $\Phi$ the cumulative distribution function of the standard normal distribution.

This can be written as

$\Phi^{-1}\left( S\left( t \right) \right)=\varepsilon\left( t \right)=\nu+ \theta log(t)$, with $\nu=\frac{\alpha}{\beta}, \theta=-\frac{1}{\beta}.$

Transitivity of relative treatment effect at time t can be proven as

$\varepsilon_{BC}(t)= \nu_{C}-\nu_{B}+{(\theta}_{C}-\theta_{B}$) $log(t)$

$=\nu_{C}-\nu_{A}+{(\theta}_{C}-\theta_{A}$)$log(t) -(\nu_{B}-\nu_{A}+{(\theta}_{B}-\theta_{A}) log(t))$

$$=\varepsilon_{AC}(t)- \varepsilon_{AB}(t)$$

*NMA method - details*

With a fixed-effects model for network meta-analysis, it was assumed that there was no variation in the treatment effect between studies. As presented in Ouwens et al. (2010), the model was defined as follows:

$\log\left( h_{jkt} \right)= \nu_{jk}+\theta_{jk}log(t)$ (1)

$$\binom{\nu_{jk}}{\theta_{jk}}=\left\{ \begin{aligned} \binom{\mu_{1jb}}{\mu_{2jb}}, \mathrm{if}k=b, b=\{everolimus,placebo,sorafenib\} (3 "baseline" treatments) \\ \binom{\mu_{1jb}}{\mu_{2jb}}+\binom{\delta_{1jbk}}{\delta_{2jbk}}, \mathrm{if}k\text{"}\text{otherwise}b, b=\{everolimus,placebo,sorafenib\} \end{aligned} \right.$$

$$\binom{\delta_{1jbk}}{\delta_{2jbk}}=\binom{d_{1Ak}}{d_{2Ak}}-\binom{d_{1Ab}}{d_{2Ab}}$$

In this formula, $h_{jkt}$ represents the underlying hazard rate in study $j$ for treatment $k$ at time point $t$. The vectors $\binom{\mu_{1jb}}{\mu_{2jb}}$ are treatment-specific and reflect the parameters $\nu$ and $\theta$ of the “baseline” treatment in study $j$. In our case study, everolimus was the “baseline” treatment in METEOR, CheckMate025, and RECORD-1; placebo was the “baseline” treatment in TARGET and sorafenib was the “baseline” treatment in AXIS. The vector $\binom{\delta_{1jbk}}{\delta_{2jbk}}$ reflects the study-specific difference in scale $\nu$ and shape $\theta$ of the log-hazard curve for treatment$k$ relative to the “baseline” treatment in study $j$. In our case study, treatment $k$ corresponded to cabozantinib in METEOR, nivolumab in CheckMate025, placebo in RECORD-1, sorafenib in TARGET and axitinib in AXIS.

Estimation of model parameters of interest – baseline and effect vectors – was performed in Bayesian framework. The prior distributions as used for the parameters of the fixed-effects model were chosen non-informative as follows:

$$\binom{\mu_{1jb}}{\mu_{2jb}} \sim N\left( \binom{0}{0}, T_{\mu} \right), T_{\mu}= \binom{{10}^{4} 0}{0 {10}^{4}}$$

$$\binom{d_{1Ak}}{d_{2Ak}} \sim N\left( \binom{0}{0}, T_{d} \right), T_{d}= \binom{{10}^{4} 0}{0 {10}^{4}}$$
