# Supplementary material for: Cabozantinib versus everolimus, nivolumab, axitinib, sorafenib and best supportive care: A network meta-analysis of progression-free survival and overall survival in second line treatment of advanced renal cell carcinoma
Source: PLoS One. 2017 Sep 8;12(9):e0184423. doi: 10.1371/journal.pone.0184423 (PMC5590935; doi:10.1371/journal.pone.0184423)
Supplement: S7 File — (DOCX) [file pone.0184423.s007.docx]

### Heterogeneity & Inconsistency

1. **Heterogeneity & inconsistency**

Given the limitation in the available data, heterogeneity or inconsistency could only be tested at the network, and not specifically by contrast In fact, there was only one trial comparing the same pair of treatments and therefore, designs (groups of trials classified according to the sets of treatments tested) always coincided with trials and the estimation of between-trial heterogeneity was confounded with the estimation of treatment effect. As a result, it was not possible to investigate measures of heterogeneity or inconsistency in this treatment network and the design-based decomposition of Cochran’s Q for assessing the homogeneity within designs and the homogeneity & consistency between designs got always null value. However, we ran additional random-effects models were run for both PFS and OS to compare with the fixed-effects models, to provide heterogeneity & inconsistency checking. We found that in terms of DIC and residual deviance presented in the Table 3 and Table 4, both effects models provided almost identical data fitting results. It numerically ensured the homogeneity and absence of inconsistency.
