# Supplementary material for: Cabozantinib versus everolimus, nivolumab, axitinib, sorafenib and best supportive care: A network meta-analysis of progression-free survival and overall survival in second line treatment of advanced renal cell carcinoma
Source: PLoS One. 2017 Sep 8;12(9):e0184423. doi: 10.1371/journal.pone.0184423 (PMC5590935; doi:10.1371/journal.pone.0184423)
Supplement: S8 File — (DOCX) [file pone.0184423.s008.docx]

### Goodness-of-fit

1. **Goodness-of-fit**

The goodness-of-fit of the model prediction to the observed individual patient data (IPD) was measured by computing the posterior mean residual deviance, $\mathrm{Dbar}$ [33]. The deviance information criterion ($\mathrm{DIC}$) was used to compare different models and provided a measure of model fit that penalized model complexity according to Spiegelhalter et al. (2002) [29]:

$$\left\{ \begin{aligned} DIC=Dbar+pD \\ pD=Dbar-Dhat \end{aligned} \right.$$

$\mathrm{pD}$ is the effective number of parameters and $\mathrm{Dhat}$ is the deviance evaluated at the posterior mean of the model parameters. The model with the lowest $\mathrm{DIC}$ provided the best data fit. The model fits were visually inspected against original published KM curves.

Model fit statistics are presented in Table 3 and Table 4, for OS and PFS respectively. In both cases, the log-normal fixed-effects model provided the best model fit with the lowest DIC.
