# Supplementary material for: Cabozantinib versus everolimus, nivolumab, axitinib, sorafenib and best supportive care: A network meta-analysis of progression-free survival and overall survival in second line treatment of advanced renal cell carcinoma
Source: PLoS One. 2017 Sep 8;12(9):e0184423. doi: 10.1371/journal.pone.0184423 (PMC5590935; doi:10.1371/journal.pone.0184423)

### Additional results

1. **Additional results**

Estimates of parameters of each survival curve (Weibull, Gompertz, log-logistic, log-normal and exponential) with 95% credible intervals are presented in Table 2 below.

Table 2. Parameter estimates with 95% credible intervals of Weibull, Gompertz, log-logistic, log-normal and Exponential distributions for fixed-effects network meta-analysis

|  |  | **OS** | | **PFS** | |
| --- | --- | --- | --- | --- | --- |
| **Weibull** | **Distribution parameters** | **Scale** $\boldsymbol{\lambda}$ **(95% credible intervals)** | **Shape** $\boldsymbol{ϒ}$ **(95% credible intervals)** | **Scale** $\boldsymbol{\lambda}$ **(95% credible intervals)** | **Shape** $\boldsymbol{ϒ}$ **(95% credible intervals)** |
|  | **Everolimus** | 0.015 (0.009, 0.024) | 1.364 (1.194, 1.541) | 0.086 (0.064, 0.116) | 1.317 (1.181, 1.460) |
|  | **Cabozantinib** | 0.006 (0.003, 0.014) | 1.497 (1.261, 1.744) | 0.035 (0.023, 0.053) | 1.418 (1.239, 1.602) |
|  | **Nivolumab** | 0.005 (0.002, 0.012) | 1.627 (1.345, 1.930) | 0.100 (0.064, 0.157) | 1.167 (0.979, 1.365) |
|  | **Placebo** | 0.014 (0.003, 0.044) | 1.635 (1.165, 2.234) | 0.207 (0.101, 0.433) | 1.635 (1.207, 2.099) |
|  | **Sorafenib** | 0.016 (0.002, 0.066) | 1.388 (0.870, 2.116) | 0.053 (0.024, 0.112) | 2.088 (1.633, 2.588) |
|  | **Axitinib** | 0.014 (0.002, 0.073) | 1.427 (0.867, 2.176) | 0.042 (0.017, 0.103) | 2.015 (1.512, 2.568) |
| **Gompertz** | **Distribution parameters** | **Shape** $\boldsymbol{a}$ **(95% CI)** | **Scale** $\boldsymbol{b}$ **(95% CI)** | **Shape** $\boldsymbol{a}$ **(95% CI)** | **Scale** $\boldsymbol{b}$ **(95% CI)** |
|  | **Everolimus** | 0.028 (0.021, 0.037) | 0.041 (0.018, 0.065) | 0.140 (0.114, 0.171) | 0.026 (-0.016, 0.067) |
|  | **Cabozantinib** | 0.018 (0.012, 0.024) | 0.048 (0.020, 0.074) | 0.065 (0.050, 0.083) | 0.060 (0.019, 0.099) |
|  | **Nivolumab** | 0.016 (0.010, 0.025) | 0.067 (0.030, 0.104) | 0.153 (0.111, 0.203) | 0.012 (-0.064, 0.038) |
|  | **Placebo** | 0.026 (0.014, 0.050) | 0.143 (0.052, 0.229) | 0.279 (0.155, 0.475) | 0.248 (0.082, 0.425) |
|  | **Sorafenib** | 0.027 (0.013, 0.054) | 0.085 (-0.003, 0.176) | 0.104 (0.054, 0.186) | 0.364 (0.192, 0.547) |
|  | **Axitinib** | 0.028 (0.012, 0.063) | 0.082 (-0.008, 0.179) | 0.078 (0.038, 0.156) | 0.363 (0.176, 0.559) |
| **Log-logistic** | **Distribution parameters** | **Scale** $\boldsymbol{a}$ **(95% CI)** | **Shape** $\boldsymbol{b}$ **(95% CI)** | **Scale** $\boldsymbol{a}$ **(95% CI)** | **Shape** $\boldsymbol{b}$ **(95% CI)** |
|  | **Everolimus** | 0.010 (0.005, 0.018) | 1.631 (1.428, 1.849) | 0.056 (0.040, 0.079) | 1.968 (1.763, 2.189) |
|  | **Cabozantinib** | 0.004 (0.002, 0.009) | 1.724 (1.479, 1.998) | 0.024 (0.014, 0.037) | 1.825 (1.599, 2.070) |
|  | **Nivolumab** | 0.002 (0.001, 0.008) | 1.981 (1.591, 2.465) | 0.064 (0.036, 0.105) | 1.778 (1.501, 2.101) |
|  | **Placebo** | 0.014 (0.003, 0.041) | 1.761 (1.297, 2.364) | 0.093 (0.044, 0.173) | 3.033 (2.358, 3.901) |
|  | **Sorafenib** | 0.013 (0.002, 0.044) | 1.586 (1.122, 2.206) | 0.011 (0.002, 0.036) | 3.373 (2.532, 4.479) |
|  | **Axitinib** | 0.010 (0.001, 0.045) | 1.697 (1.138, 2.508) | 0.010 (0.001, 0.042) | 2.875 (1.984, 4.135) |
| **Log-normal** | **Distribution parameters** | **Location** $\boldsymbol{\mu}$ **(95% CI)** | **Scale** $\boldsymbol{\sigma}$ **(95% CI)** | **Location** $\boldsymbol{\mu}$ **(95% CI)** | **Scale** $\boldsymbol{\sigma}$ **(95% CI)** |
|  | **Everolimus** | 2.792 (2.658, 2.933) | 1.077 (0.963, 1.211) | 1.481 (1.381, 1.590) | 0.858 (0.779, 0.948) |
|  | **Cabozantinib** | 3.130 (2.985, 3.293) | 1.030 (0.911, 1.179) | 2.057 (1.936, 2.183) | 0.942 (0.840, 1.064) |
|  | **Nivolumab** | 3.033 (2.777, 3.293) | 0.917 (0.760, 1.113) | 1.620 (1.435, 1.802) | 0.932 (0.804, 1.085) |
|  | **Placebo** | 2.442 (2.158, 2.740) | 1.032 (0.802, 1.344) | 0.851 (0.666, 1.038) | 0.591 (0.471, 0.746) |
|  | **Sorafenib** | 2.760 (2.444, 3.091) | 1.131 (0.849, 1.531) | 1.310 (1.105, 1.528) | 0.572 (0.446, 0.744) |
|  | **Axitinib** | 2.755 (2.358, 3.159) | 1.028 (0.729, 1.479) | 1.594 (1.298, 1.904) | 0.660 (0.479, 0.915) |
| **Exponential** | **Distribution parameters** | **Rate** $\boldsymbol{\lambda}$ **(95% CI)** | | **Rate** $\boldsymbol{\lambda}$ **(95% CI)** | |
|  | **Everolimus** | 0.041 (0.036, 0.048) | | 0.155 (0.135, 0.177) | |
|  | **Cabozantinib** | 0.028 (0.024, 0.033) | | 0.086 (0.074, 0.100) | |
|  | **Nivolumab** | 0.032 (0.025, 0.040) | | 0.129 (0.105, 0.159) | |
|  | **Placebo** | 0.057 (0.041, 0.078) | | 0.371 (0.265, 0.515) | |
|  | **Sorafenib** | 0.041 (0.028, 0.059) | | 0.234 (0.163, 0.336) | |
|  | **Axitinib** | 0.041 (0.026, 0.064) | | 0.173 (0.109, 0.268) | |

Fig 7. Averaged PFS curves over time derived from the Weibull fixed-effects model, adjusted to the baseline from METEOR study, with shaded areas representing 95% credible intervals.


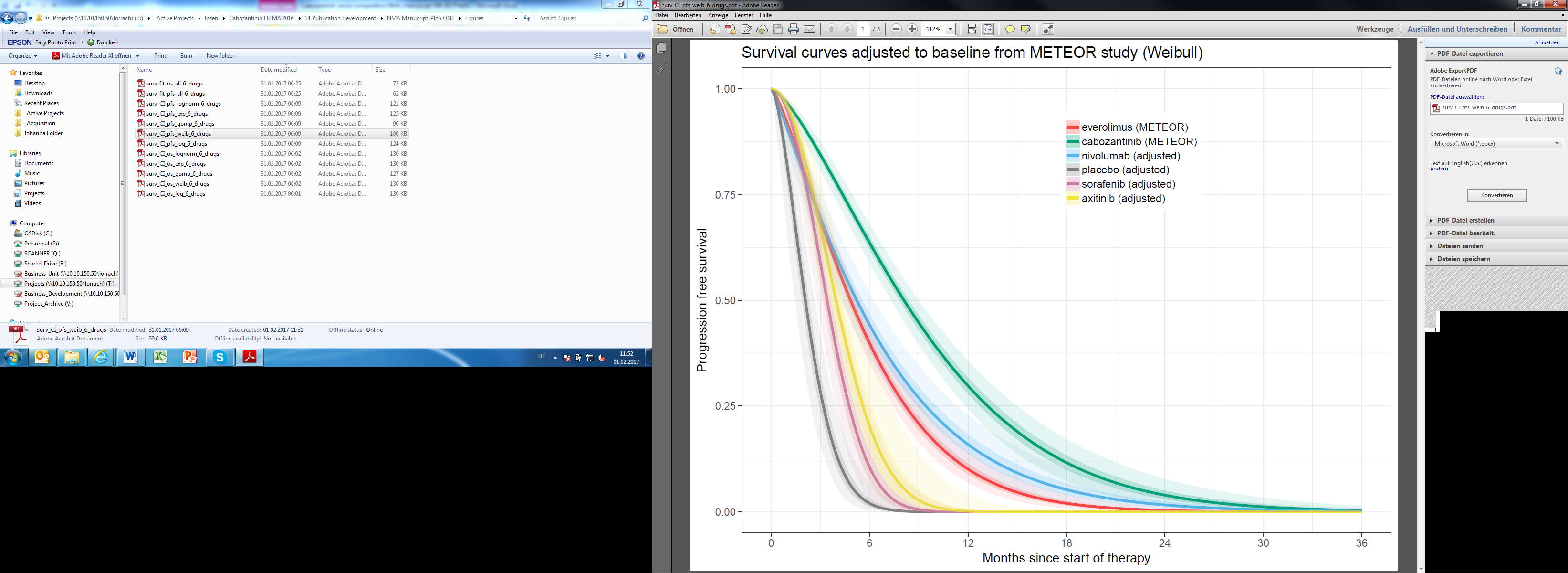


Fig 8. Averaged PFS over time derived from the Gompertz fixed-effects model, adjusted to the baseline from METEOR study, with shaded areas representing 95% credible intervals.


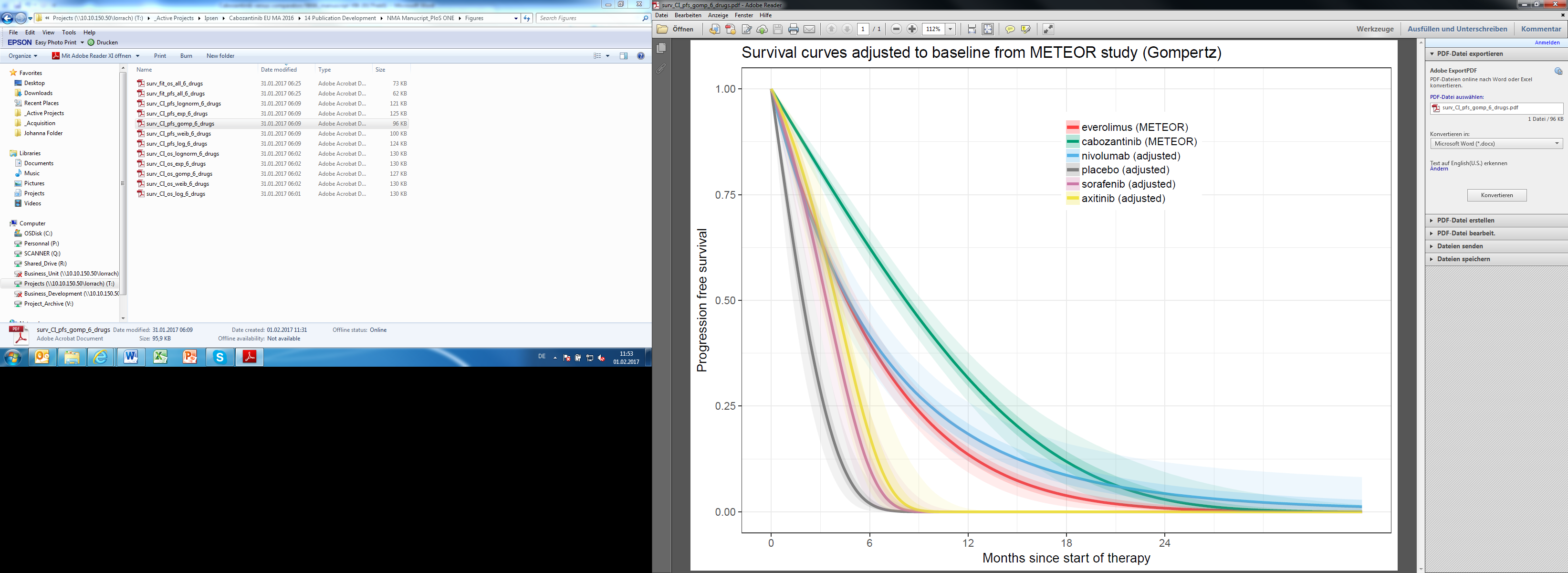


Fig 9. Averaged PFS over time derived from the Log-logistic fixed-effects model, adjusted to the baseline from METEOR study, with shaded areas representing 95% credible intervals.


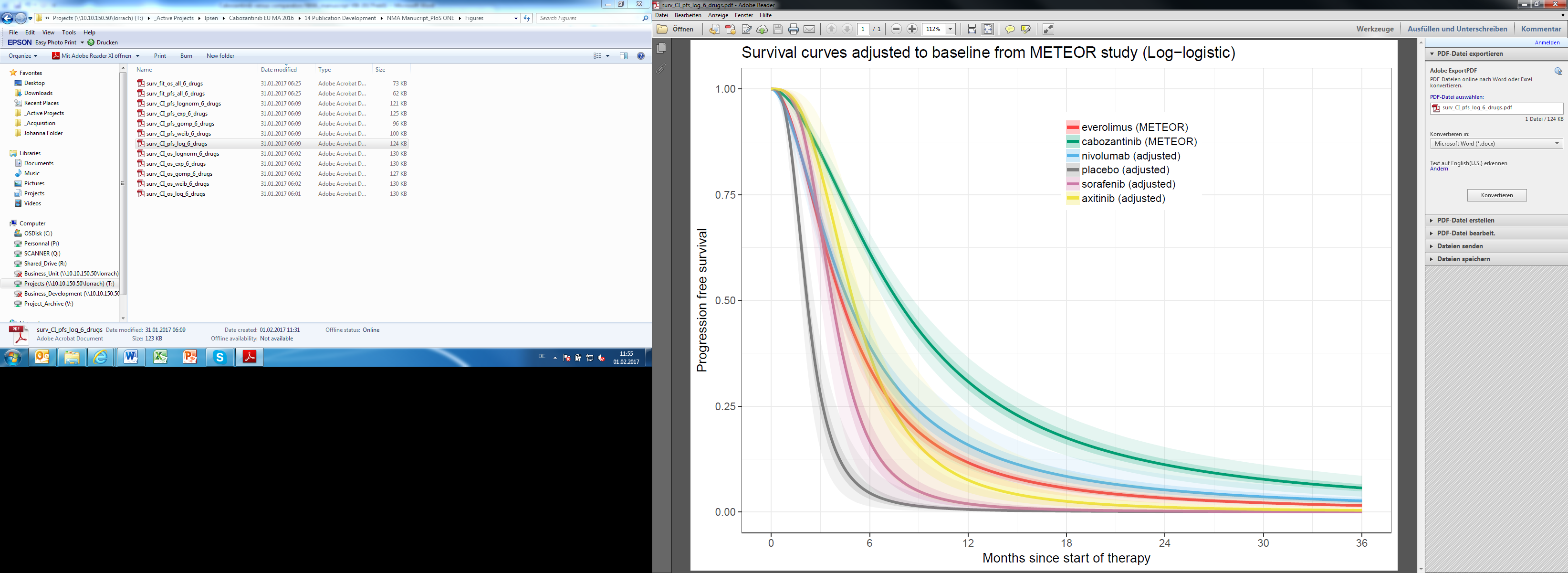


Fig 10. Averaged PFS over time derived from the exponential fixed-effects model, adjusted to the baseline from METEOR study, with shaded areas representing 95% credible intervals.


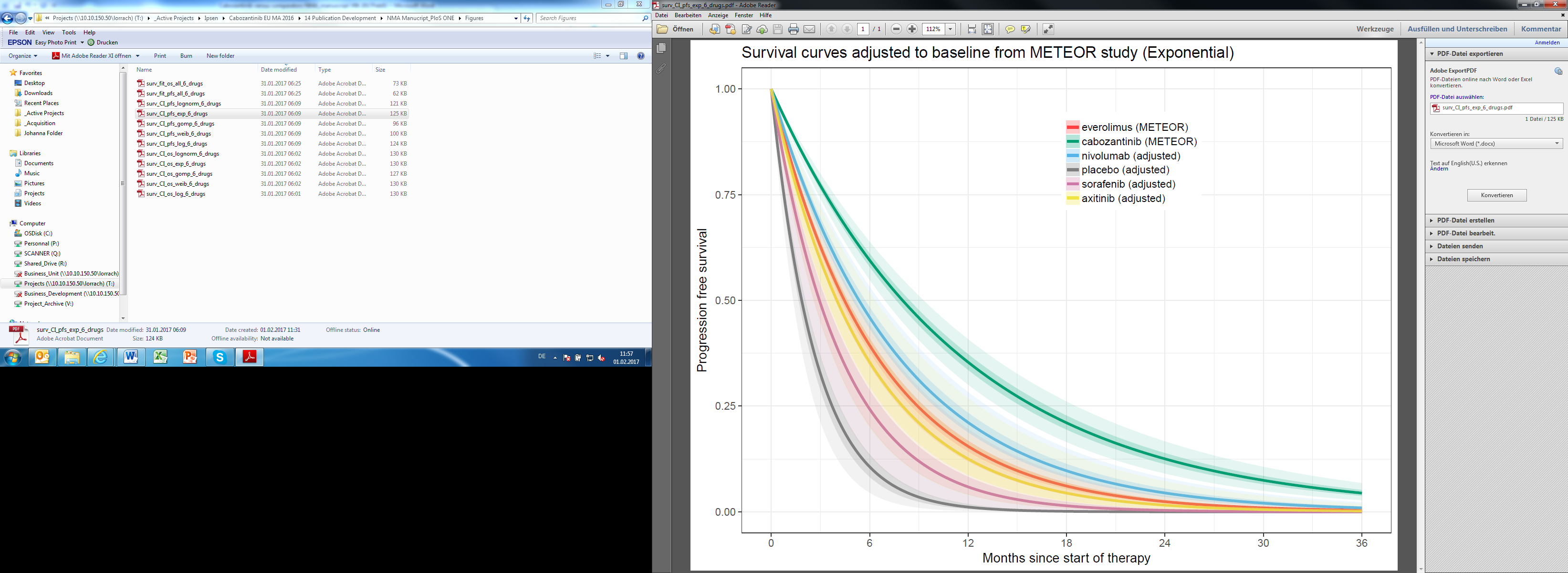


Fig 11. Averaged OS over time derived from the Weibull fixed-effects model, adjusted to the baseline from METEOR study, with shaded areas representing 95% credible intervals.


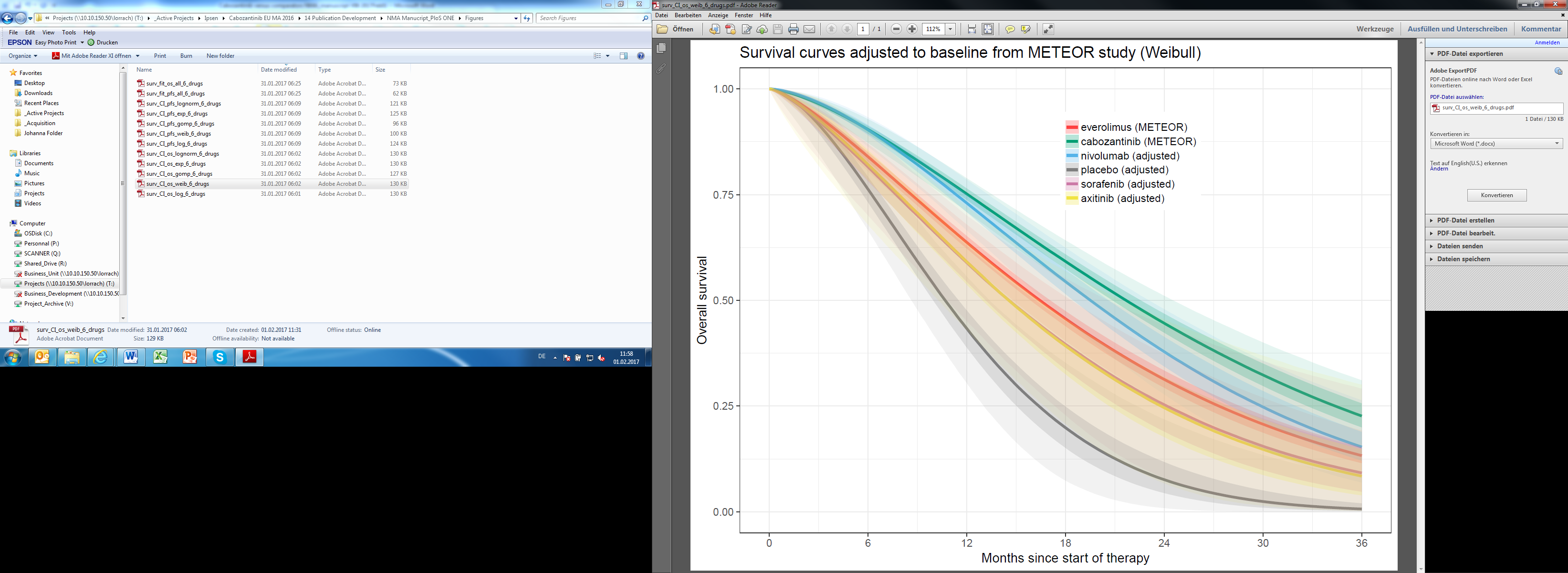


Fig 12. Averaged OS curves over time derived from the Gompertz fixed-effects model, adjusted to the baseline from METEOR study, with shaded areas representing 95% credible intervals.


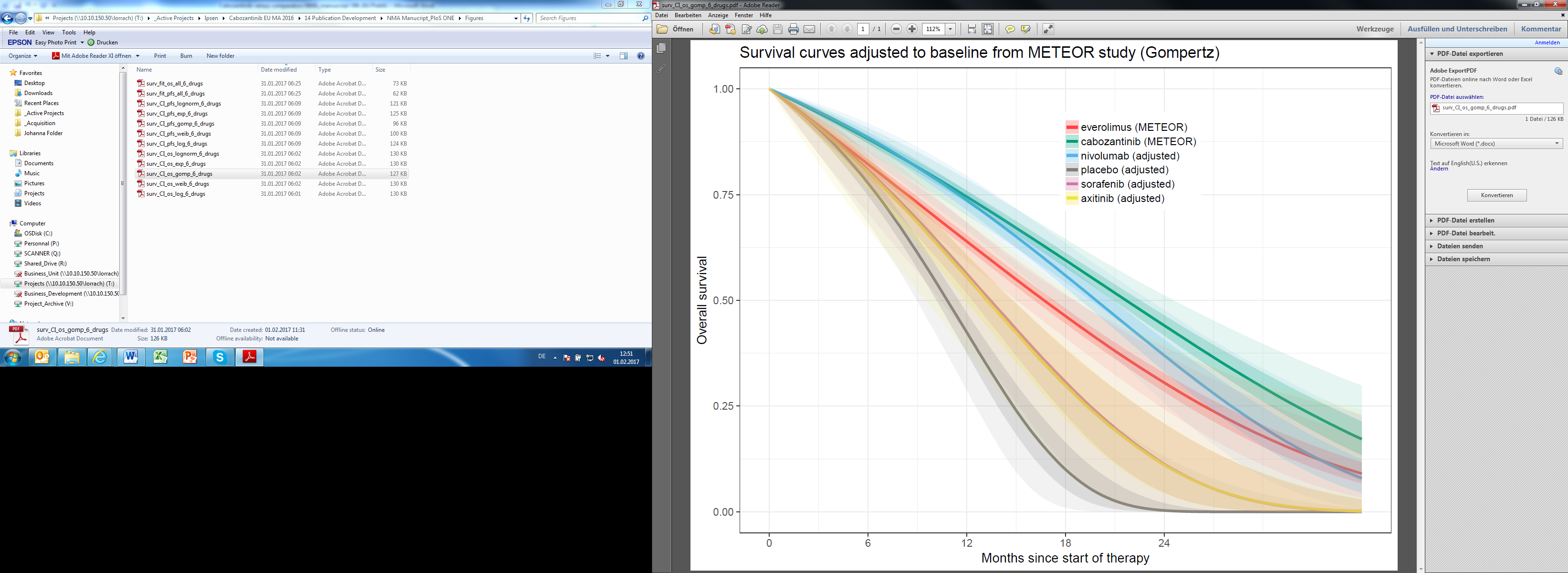


Fig 13. Averaged OS curves over time derived from the Log-logistic fixed-effects model, adjusted to the baseline from METEOR study, with shaded areas representing 95% credible intervals.


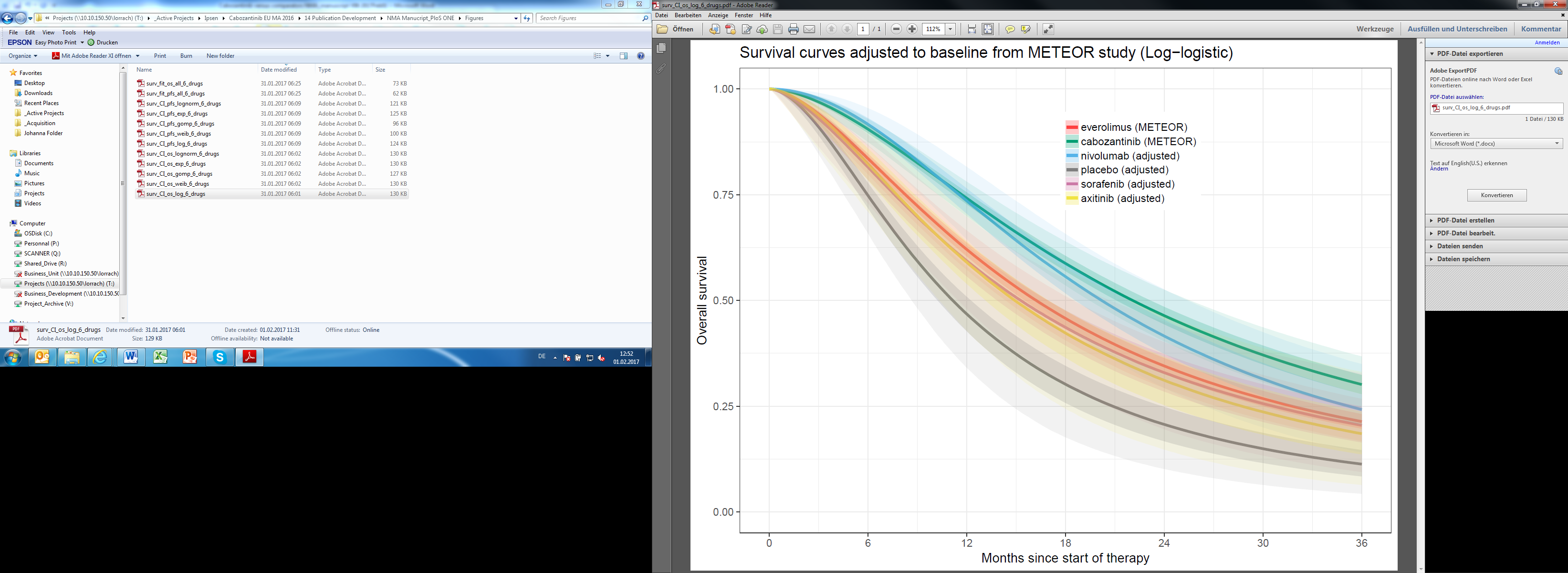


Fig 14. Averaged OS curves over time derived from the exponential fixed-effects model, adjusted to the baseline from METEOR study, with shaded areas representing 95% credible intervals.


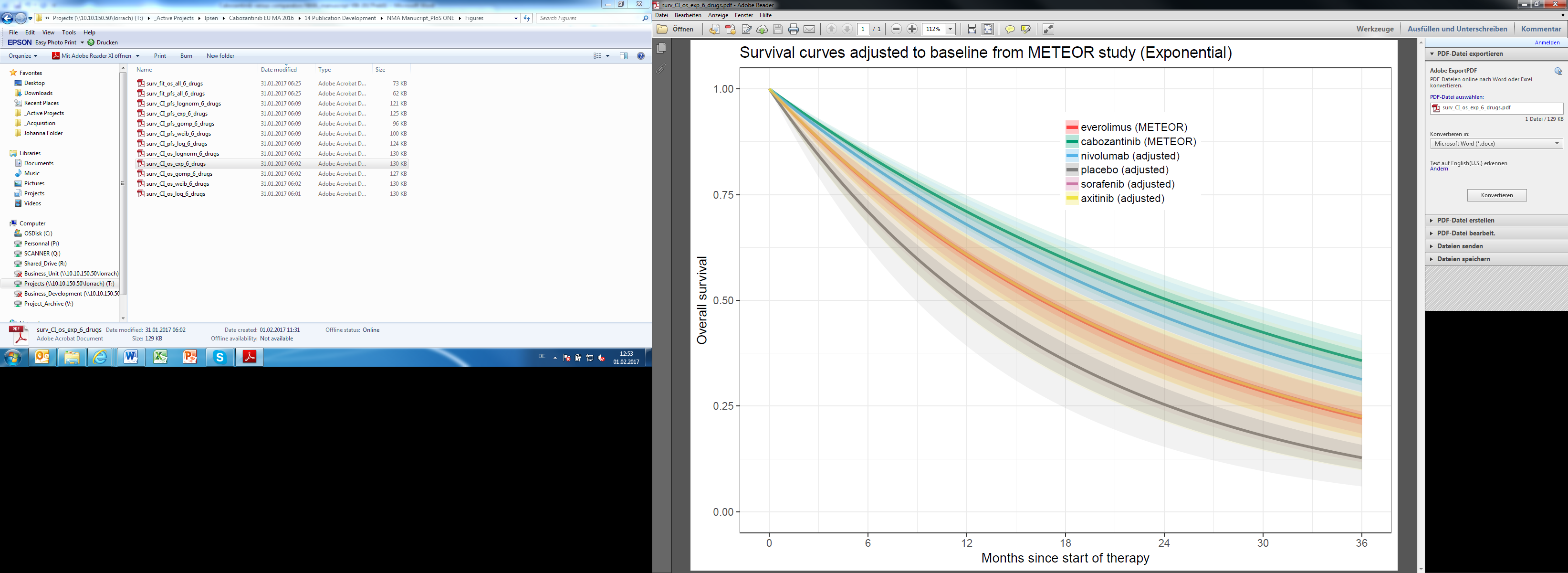

Supplement: S9 File — (DOCX) [file pone.0184423.s009.docx]
