# Supplementary material for: Cabozantinib versus everolimus, nivolumab, axitinib, sorafenib and best supportive care: A network meta-analysis of progression-free survival and overall survival in second line treatment of advanced renal cell carcinoma
Source: PLoS One. 2017 Sep 8;12(9):e0184423. doi: 10.1371/journal.pone.0184423 (PMC5590935; doi:10.1371/journal.pone.0184423)
Supplement: S10 File — (DOCX) [file pone.0184423.s010.docx]

**List of abbreviations**

**S10 List of abbreviations**

| aRCC | Advanced renal cell carcinoma |
| --- | --- |
| BSC | Best supportive care |
| CI | Confidence interval |
| ESMO | European Society for Medical Oncology |
| EUA | European Association of Urology |
| FE | Fixed effect |
| HRs | Hazard ratios |
| INV | Investigators |
| ITT | Intent-to-treat |
| IRC | Independent review committee |
| KM | Kaplan-Meier |
| MCMC | Markov Chain Monte Carlo |
| MSKCC | Memorial Sloan-Kettering Cancer Center |
| mTOR | Mammalian target of rapamycin |
| NCCN | National Comprehensive Cancer Network |
| NICE | National Institute for Health and Care Excellence |
| NMA | Network meta-analysis |
| PFS | Progression-free survival |
| PH | Proportional hazard |
| OS | Overall survival |
| RCC | Renal cell carcinoma |
| RCTs | Random controlled trials |
| RE | Random effect |
| STA | Single technology appraisal |
| TKI | Tyrosine-kinase inhibitor |
| VEGFR | Vascular endothelial growth factor receptor |
