# Supplementary material for: Cabozantinib versus everolimus, nivolumab, axitinib, sorafenib and best supportive care: A network meta-analysis of progression-free survival and overall survival in second line treatment of advanced renal cell carcinoma
Source: PLoS One. 2017 Sep 8;12(9):e0184423. doi: 10.1371/journal.pone.0184423 (PMC5590935; doi:10.1371/journal.pone.0184423)

### Additional results

**S 11 Additional results – Fixed effect, restricted network (METEOR and CheckMate only)**

Estimates of parameters of each survival curve (Weibull, Gompertz, log-logistic, log-normal and exponential) with 95% credible intervals are presented in Table 2 below.

Table 2. Parameter estimates with 95% credible intervals of Weibull, Gompertz, log-logistic, log-normal and Exponential distributions for fixed-effects network meta-analysis

|  |  | **OS** | | **PFS** | |
| --- | --- | --- | --- | --- | --- |
| **Weibull** | **Distribution parameters** | **Scale** $\boldsymbol{\lambda}$ **(95% credible intervals)** | **Shape** $\boldsymbol{ϒ}$ **(95% credible intervals)** | **Scale** $\boldsymbol{\lambda}$ **(95% credible intervals)** | **Shape** $\boldsymbol{ϒ}$ **(95% credible intervals)** |
|  | **Everolimus** | 0.017 (0.011, 0.024) | 1.334 (1.190, 1.491) | 0.090 (0.070, 0.117) | 1.300 (1.173, 1.423) |
|  | **Cabozantinib** | 0.006 (0.003, 0.011) | 1.559 (1.337, 1.796) | 0.033 (0.022, 0.050) | 1.449 (1.264, 1.632) |
|  | **Nivolumab** | 0.006 (0.002, 0.011) | 1.618 (1.387, 1.901) | 0.104 (0.069, 0.158) | 1.152 (0.973, 1.337) |
| **Gompertz** | **Distribution parameters** | **Shape** $\boldsymbol{a}$ **(95% CI)** | **Scale** $\boldsymbol{b}$ **(95% CI)** | **Shape** $\boldsymbol{a}$ **(95% CI)** | **Scale** $\boldsymbol{b}$ **(95% CI)** |
|  | **Everolimus** | 0.030 (0.024, 0.03  7) | 0.039 (0.017, 0.061) | 0.143 (0.120, 0.173) | 0.024 (-0.017, 0.061) |
|  | **Cabozantinib** | 0.017 (0.013, 0.024) | 0.051 (0.023, 0.077) | 0.064 (0.049, 0.081) | 0.063 (0.023, 0.102) |
|  | **Nivolumab** | 0.016 (0.010, 0.025) | 0.068 (0.033, 0.104) | 0.152 (0.115, 0.203) | -0.014 (-0.064, 0.035) |
| **Log-logistic** | **Distribution parameters** | **Scale** $\boldsymbol{a}$ **(95% CI)** | **Shape** $\boldsymbol{b}$ **(95% CI)** | **Scale** $\boldsymbol{a}$ **(95% CI)** | **Shape** $\boldsymbol{b}$ **(95% CI)** |
|  | **Everolimus** | 0.011 (0.006, 0.018) | 1.623 (1.439, 1.829) | 0.057 (0.040, 0.078) | 1.964 (1.772, 2.176) |
|  | **Cabozantinib** | 0.005 (0.002, 0.010) | 1.740 (1.478, 2.004) | 0.024 (0.015, 0.038) | 1.832 (1.598, 2.067) |
|  | **Nivolumab** | 0.003 (0.001, 0.008) | 1.963 (1.587, 2.441) | 0.065 (0.036, 0.105) | 1.771 (1.504, 2.093) |
| **Log-normal** | **Distribution parameters** | **Location** $\boldsymbol{\mu}$ **(95% CI)** | **Scale** $\boldsymbol{\sigma}$ **(95% CI)** | **Location** $\boldsymbol{\mu}$ **(95% CI)** | **Scale** $\boldsymbol{\sigma}$ **(95% CI)** |
|  | **Everolimus** | 2.799 (2.672, 2.936) | 1.079 (0.969, 1.214) | 1.486 (1.385, 1.591) | 0.858 (0.784, 0.947) |
|  | **Cabozantinib** | 3.124 (2.982, 3.279) | 1.027 (0.906, 1.168) | 2.054 (1.940, 2.173) | 0.941 (0.840, 1.056) |
|  | **Nivolumab** | 3.024 (2.796, 3.271) | 0.911 (0.753, 1.109) | 1.619 (1.452, 1.797) | 0.930 (0.807, 1.077) |
| **Exponential** | **Distribution parameters** | **Rate** $\boldsymbol{\lambda}$ **(95% CI)** | | **Rate** $\boldsymbol{\lambda}$ **(95% CI)** | |
|  | **Everolimus** | 0.042 (0.036, 0.048) | | 0.156 (0.135, 0.177) | |
|  | **Cabozantinib** | 0.028 (0.024, 0.033) | | 0.086 (0.074, 0.099) | |
|  | **Nivolumab** | 0.033 (0.025, 0.041) | | 0.131 (0.105, 0.161) | |

Fig 7. Averaged PFS curves over time derived from the Weibull fixed-effects model, adjusted to the baseline from METEOR study, with shaded areas representing 95% credible intervals.


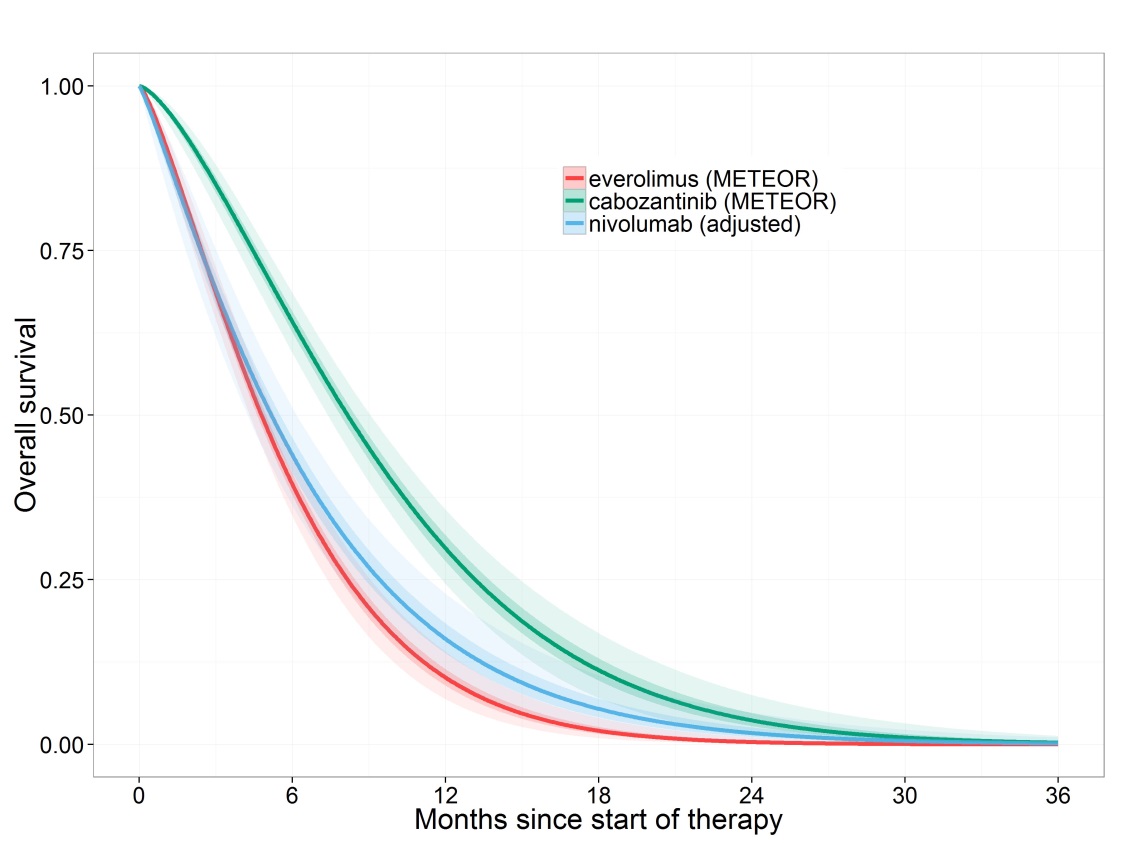


Fig 8. Averaged PFS over time derived from the Gompertz fixed-effects model, adjusted to the baseline from METEOR study, with shaded areas representing 95% credible intervals.

**
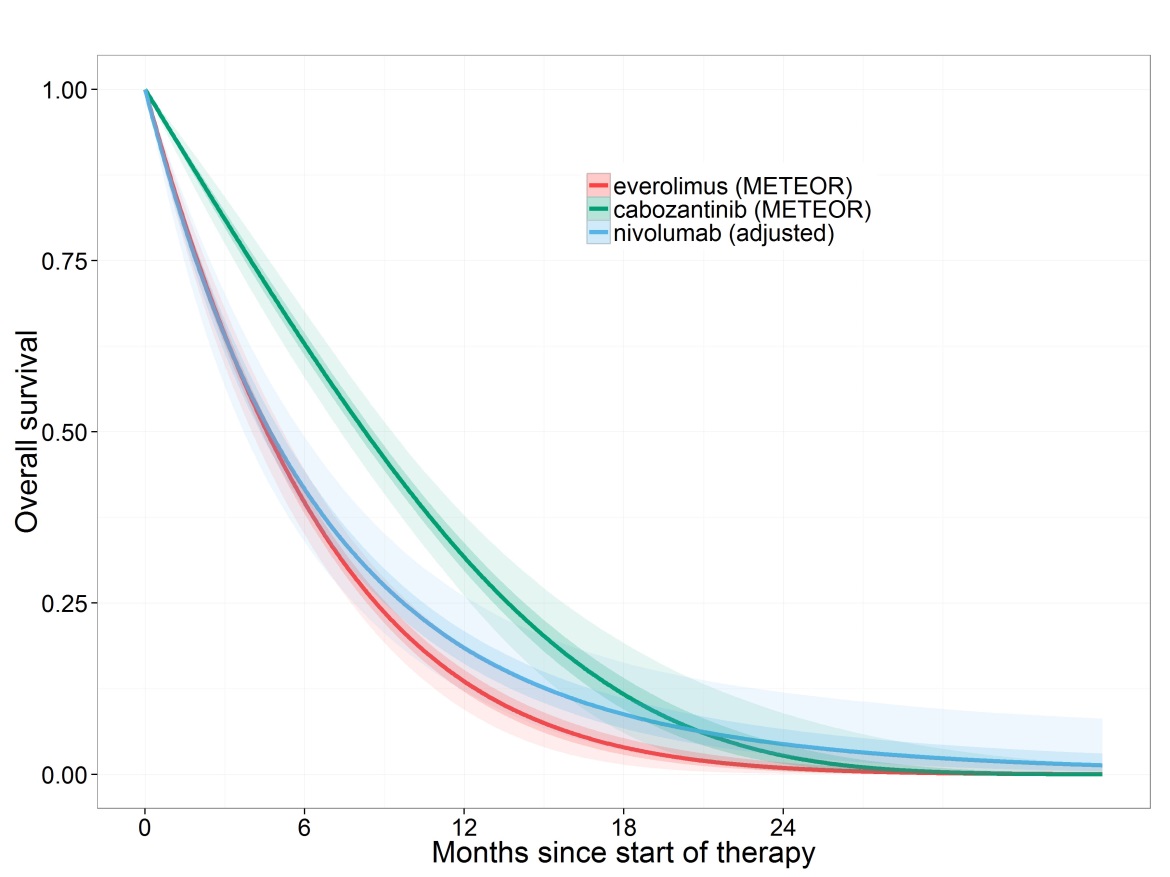
**

Fig 9. Averaged PFS over time derived from the Log-logistic fixed-effects model, adjusted to the baseline from METEOR study, with shaded areas representing 95% credible intervals.


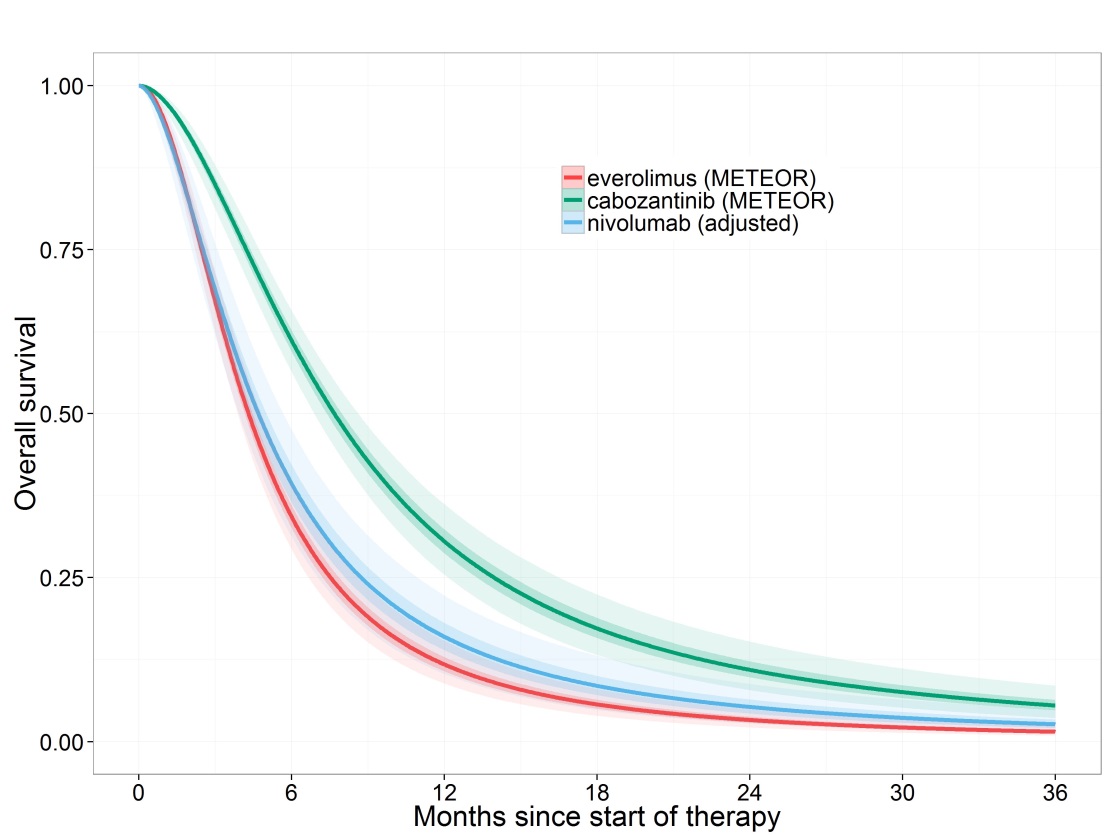


Fig 10. Averaged PFS over time derived from the exponential fixed-effects model, adjusted to the baseline from METEOR study, with shaded areas representing 95% credible intervals.

*
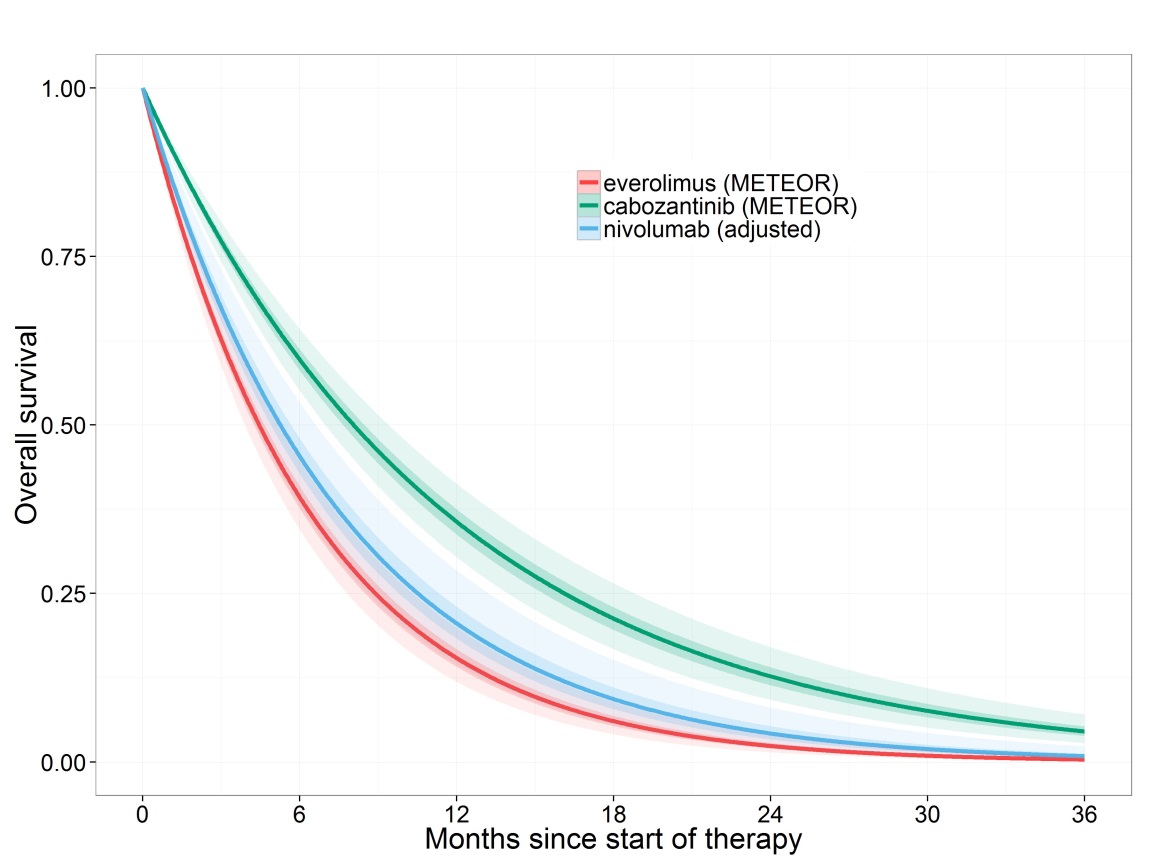
*

Fig 11. Averaged OS over time derived from the lognormal fixed-effects model, adjusted to the baseline from METEOR study, with shaded areas representing 95% credible intervals.

*
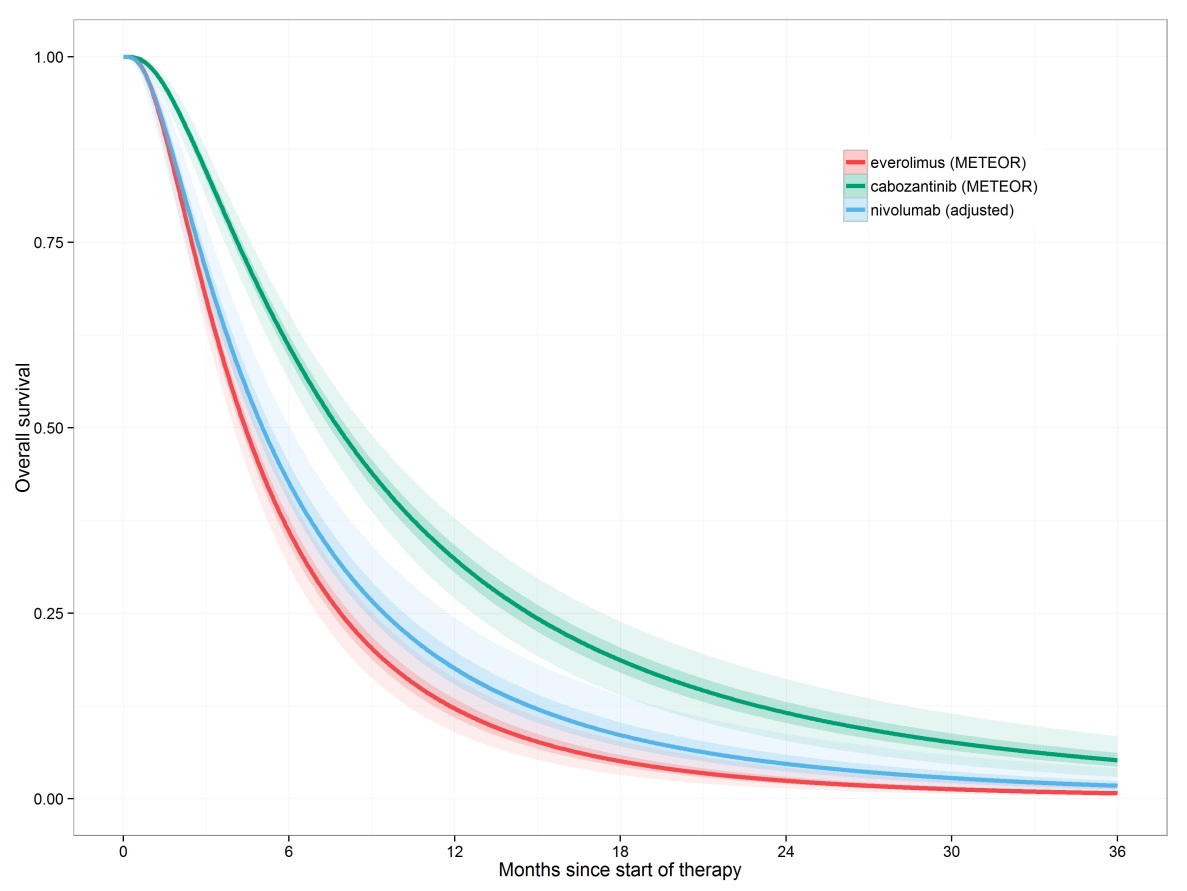
*

Fig 12. Averaged OS over time derived from the Weibull fixed-effects model, adjusted to the baseline from METEOR study, with shaded areas representing 95% credible intervals.


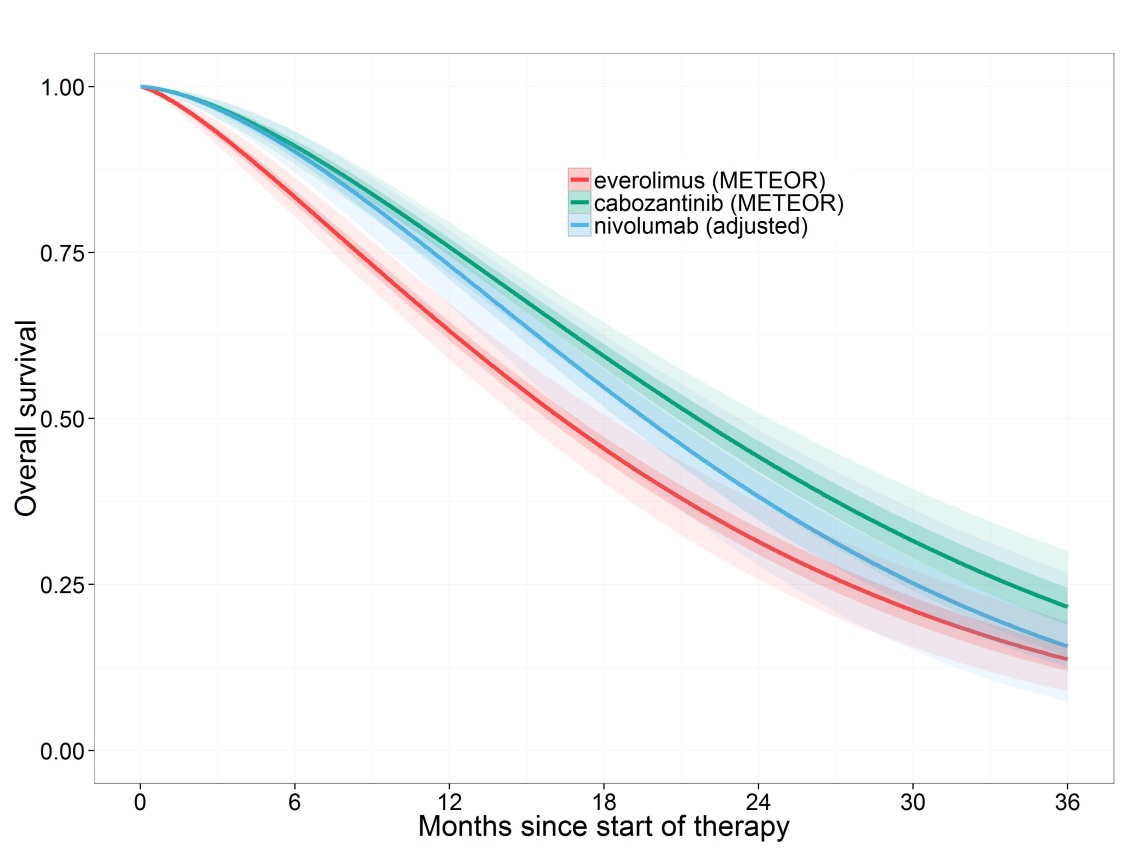


Fig 13. Averaged OS curves over time derived from the Gompertz fixed-effects model, adjusted to the baseline from METEOR study, with shaded areas representing 95% credible intervals.

**
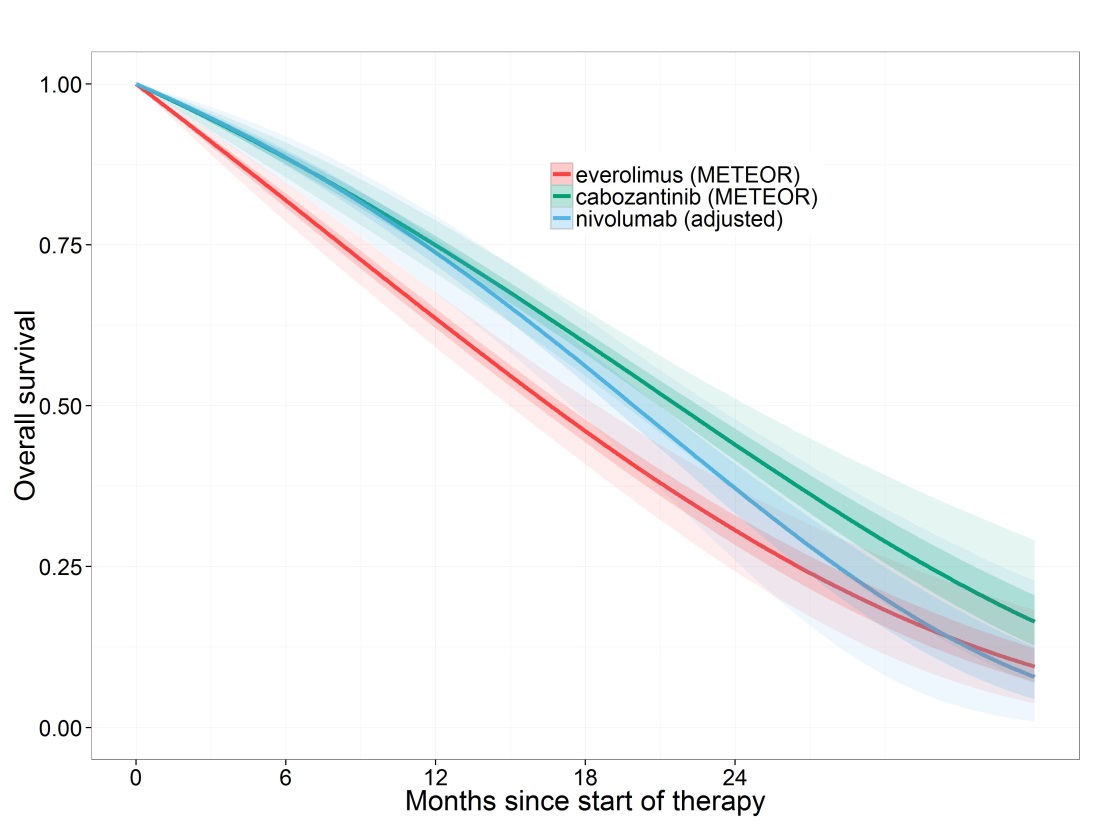
**

Fig 14. Averaged OS curves over time derived from the Log-logistic fixed-effects model, adjusted to the baseline from METEOR study, with shaded areas representing 95% credible intervals.


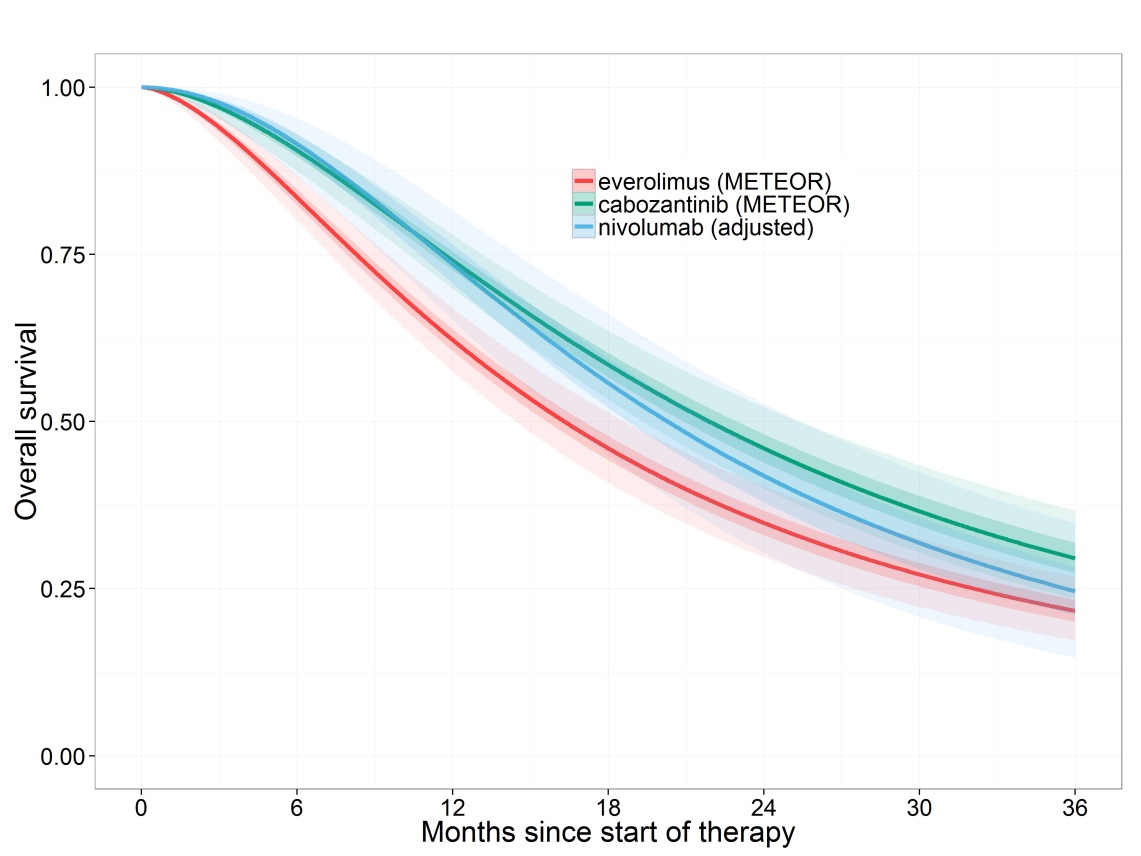


Fig 15. Averaged OS curves over time derived from the exponential fixed-effects model, adjusted to the baseline from METEOR study, with shaded areas representing 95% credible intervals.


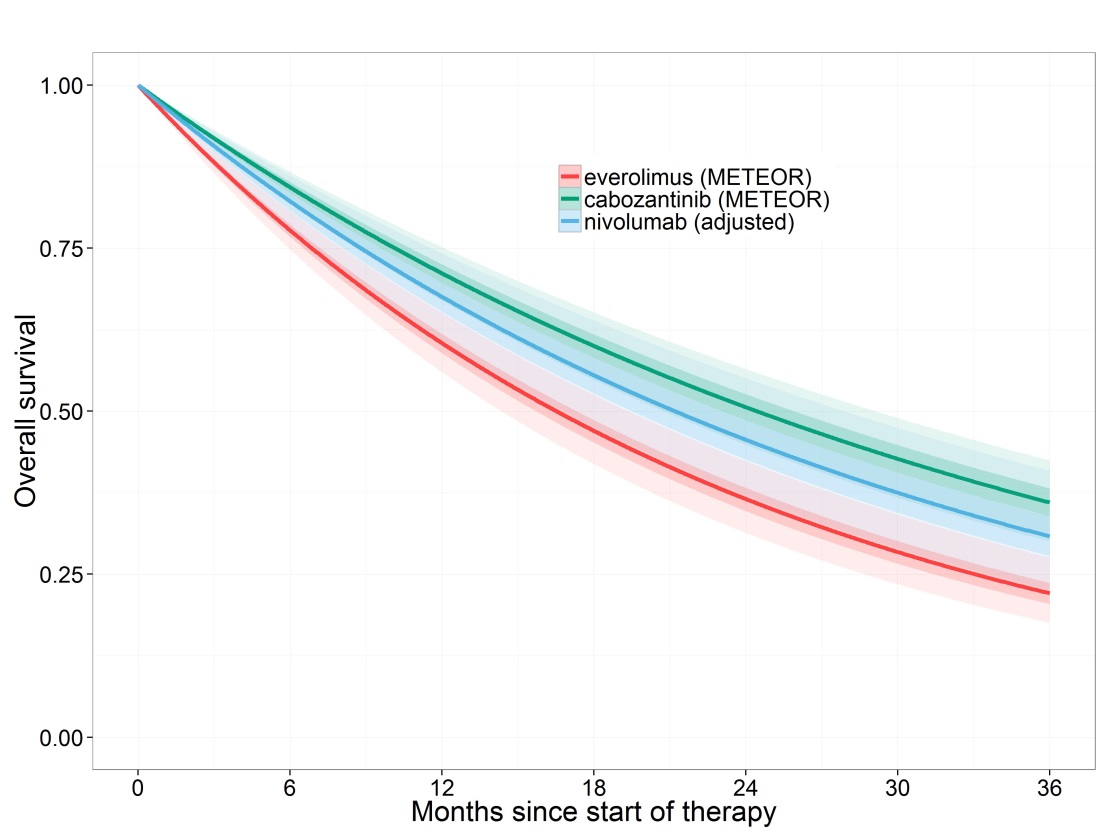


Fig 16. Averaged OS curves over time derived from the lognormal fixed-effects model, adjusted to the baseline from METEOR study, with shaded areas representing 95% credible intervals.


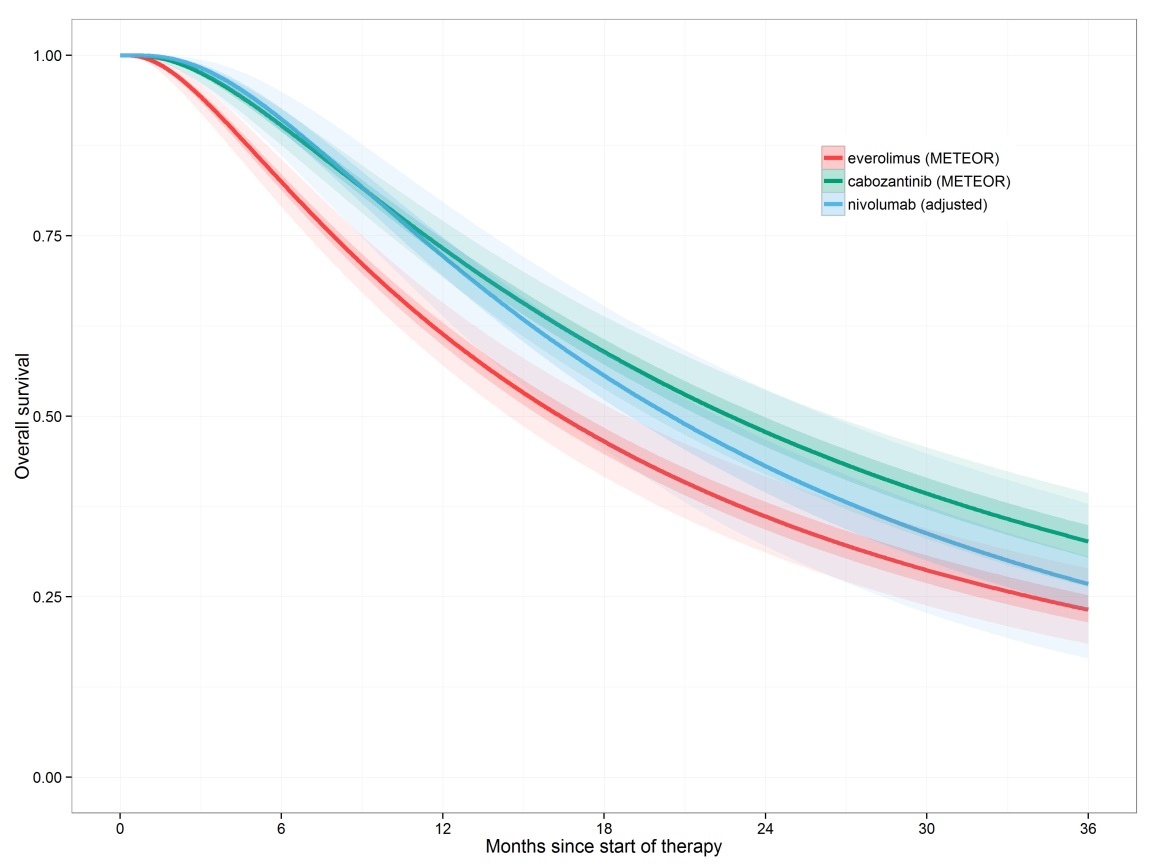

Supplement: S11 File — (DOCX) [file pone.0184423.s011.docx]
