# Supplementary material for: Cabozantinib versus everolimus, nivolumab, axitinib, sorafenib and best supportive care: A network meta-analysis of progression-free survival and overall survival in second line treatment of advanced renal cell carcinoma
Source: PLoS One. 2017 Sep 8;12(9):e0184423. doi: 10.1371/journal.pone.0184423 (PMC5590935; doi:10.1371/journal.pone.0184423)

### Additional results

**S12 Additional results – Randome effect, whole network**

Estimates of parameters of each survival curve (Weibull, Gompertz, log-logistic, log-normal and exponential) with 95% credible intervals are presented in Table 2 below.

Table 2. Parameter estimates with 95% credible intervals of Weibull, Gompertz, log-logistic, log-normal and Exponential distributions for fixed-effects network meta-analysis

|  |  | **OS** | | **PFS** | |
| --- | --- | --- | --- | --- | --- |
| **Weibull** | **Distribution parameters** | **Scale** $\boldsymbol{\lambda}$ **(95% credible intervals)** | **Shape** $\boldsymbol{ϒ}$ **(95% credible intervals)** | **Scale** $\boldsymbol{\lambda}$ **(95% credible intervals)** | **Shape** $\boldsymbol{ϒ}$ **(95% credible intervals)** |
|  | **Everolimus** | 0.015 (0.010, 0.024) | 1.362 (1.205, 1.515) | 0.086 (0.063, 0.115) | 1.321 (1.176, 1.471) |
|  | **Cabozantinib** | 0.007 (0.004, 0.013) | 1.492 (1.278, 1.695) | 0.036 (0.023, 0.057) | 1.414 (1.191, 1.631) |
|  | **Nivolumab** | 0.005 (0.003, 0.011) | 1.644 (1.381, 1.903) | 0.098 (0.059, 0.163) | 1.171 (0.941, 1.421) |
|  | **Placebo** | 0.016 (0.004,0.041) | 1.604 (1.206, 2.140) | 0.196 (0.099, 0.419) | 1.671 (1.209, 2.110) |
|  | **Sorafenib** | 0.020 (0.005, 0.057) | 1.324 (0.920, 1.843) | 0.050 (0.022, 0.113) | 2.121 (1.597, 2.614) |
|  | **Axitinib** | 0.025 (0.006, 0.114) | 1.270 (0.777, 1.842) | 0.043 (0.015, 0.115) | 2.026 (1.425, 2.622) |
| **Gompertz** | **Distribution parameters** | **Shape** $\boldsymbol{a}$ **(95% CI)** | **Scale** $\boldsymbol{b}$ **(95% CI)** | **Shape** $\boldsymbol{a}$ **(95% CI)** | **Scale** $\boldsymbol{b}$ **(95% CI)** |
|  | **Everolimus** | 0.029 (0.022, 0.037) | 0.041 (0.017, 0.066) | 0.140 (0.114, 0.171) | 0.027 (-0.015, 0.069) |
|  | **Cabozantinib** | 0.018 (0.013, 0.026) | 0.048 (-0.072, 0.168) | 0.065 (0.050, 0.087) | 0.059 (-0.060, 0.184) |
|  | **Nivolumab** | 0.016 (0.010, 0.025) | 0.068 (-0.053, 0.190) | 0.149 (0.106, 0.212) | -0.015 (-0.139, 0.117) |
|  | **Placebo** | 0.028 (0.012, 0.051) | 0.132 (-0.005, 0.293) | 0.283 (0.147, 0.471) | 0.247 (0.046, 0.466) |
|  | **Sorafenib** | 0.030 (0.011, 0.062) | 0.072 (-0.107, 0.264) | 0.106 (0.053, 0.198) | 0.360 (0.128, 0.607) |
|  | **Axitinib** | 0.032 (0.011, 0.076) | 0.071 (-0.143, 0.291) | 0.084 (0.037,0.175) | 0.355 (0.087, 0.635) |
| **Log-logistic** | **Distribution parameters** | **Scale** $\boldsymbol{a}$ **(95% CI)** | **Shape** $\boldsymbol{b}$ **(95% CI)** | **Scale** $\boldsymbol{a}$ **(95% CI)** | **Shape** $\boldsymbol{b}$ **(95% CI)** |
|  | **Everolimus** | 0.011 (0.006, 0.018) | 1.632 (1.431, 1.841) | 0.057 (0.040, 0.080) | 1.969 (1.764, 2.182) |
|  | **Cabozantinib** | 0.005 (0.000, 0.032) | 1.723 (1.163, 2.539) | 0.024 (0.005, 0.102) | 1.823 (1.234, 2.675) |
|  | **Nivolumab** | 0.003 (0.000, 0.025) | 1.970 (1.294,2.936) | 0.065 (0.017, 0.226) | 1.771 (1.175, 2.606) |
|  | **Placebo** | 0.014 (0.001, 0.074) | 1.776 (1.160, 2.781) | 0.092 (0.019, 0.423) | 3.055 (2.029, 4.758) |
|  | **Sorafenib** | 0.013 (0.001, 0.105) | 1.583 (0.919, 2.805) | 0.012 (0.000, 0.190) | 3.365 (1.966, 5.870) |
|  | **Axitinib** | 0.010 (0.000, 0.149) | 1.695 (0.858, 3.344) | 0.010 (0.000, 0.232) | 2.866 (1.454, 5.594) |
| **Log-normal** | **Distribution parameters** | **Location** $\boldsymbol{\mu}$ **(95% CI)** | **Scale** $\boldsymbol{\sigma}$ **(95% CI)** | **Location** $\boldsymbol{\mu}$ **(95% CI)** | **Scale** $\boldsymbol{\sigma}$ **(95% CI)** |
|  | **Everolimus** | 2.792 (2.656, 2.933) | 1.077 (0.966, 1.203) | 1.481 (1.377, 1.584) | 0.858 (0.781, 0.943) |
|  | **Cabozantinib** | 3.129 (2.737, 3.529) | 1.029 (0.702, 1.498) | 2.056 (1.680, 2.452) | 0.941 (0.640, 1.377) |
|  | **Nivolumab** | 3.020 (2.569, 3.468) | 0.909 (0.603, 1.356) | 1.612 (1.186, 2.026) | 0.926 (0.618, 1.367) |
|  | **Placebo** | 2.439 (1.982, 2.871) | 1.027 (0.682, 1.601) | 0.853 (0.426, 1.240) | 0.590 (0.395, 0.902) |
|  | **Sorafenib** | 2.755 (2.165, 3.315) | 1.113 (0.651, 1.930) | 1.311 (0.750, 1.830) | 0.565 (0.334, 0.968) |
|  | **Axitinib** | 2.745 (2.032, 3.458) | 1.001 (0.513, 1.939) | 1.591 (0.913, 2.269) | 0.650 (0.332, 1.258) |
| **Exponential** | **Distribution parameters** | **Rate** $\boldsymbol{\lambda}$ **(95% CI)** | | **Rate** $\boldsymbol{\lambda}$ **(95% CI)** | |
|  | **Everolimus** | 0.042 (0.036, 0.048) | | 0.155 (0.134, 0.177) | |
|  | **Cabozantinib** | 0.028 (0.016, 0.052) | | 0.086 (0.047, 0.158) | |
|  | **Nivolumab** | 0.032 (0.018, 0.058) | | 0.130 (0.072, 0.234) | |
|  | **Placebo** | 0.056 (0.029, 0.109) | | 0.362 (0.188, 0.706) | |
|  | **Sorafenib** | 0.041 (0.017, 0.098) | | 0.231 (0.099, 0.562) | |
|  | **Axitinib** | 0.041 (0.015, 0.118) | | 0.171 (0.061, 0.490) | |

Fig 7. Averaged PFS curves over time derived from the Weibull random-effects model, adjusted to the baseline from METEOR study, with shaded areas representing 95% credible intervals.


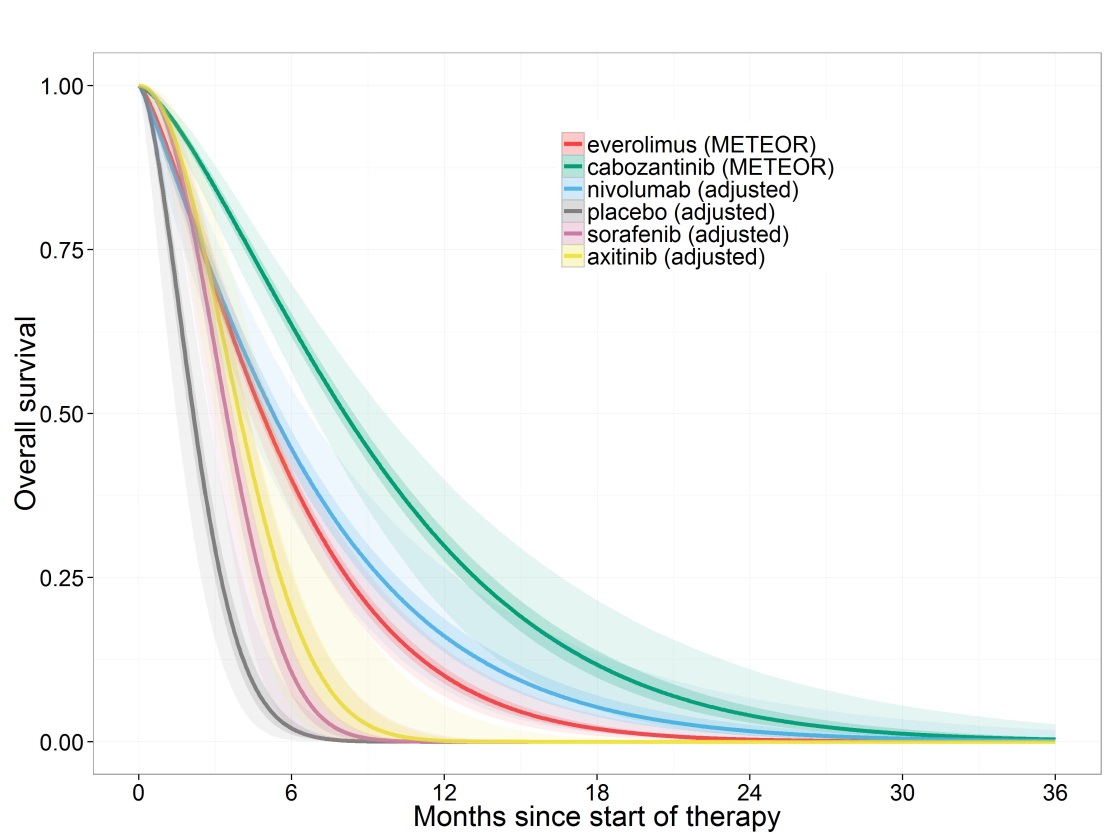


Fig 8. Averaged PFS over time derived from the Gompertz fixed-effects model, adjusted to the baseline from METEOR study, with shaded areas representing 95% credible intervals.

**
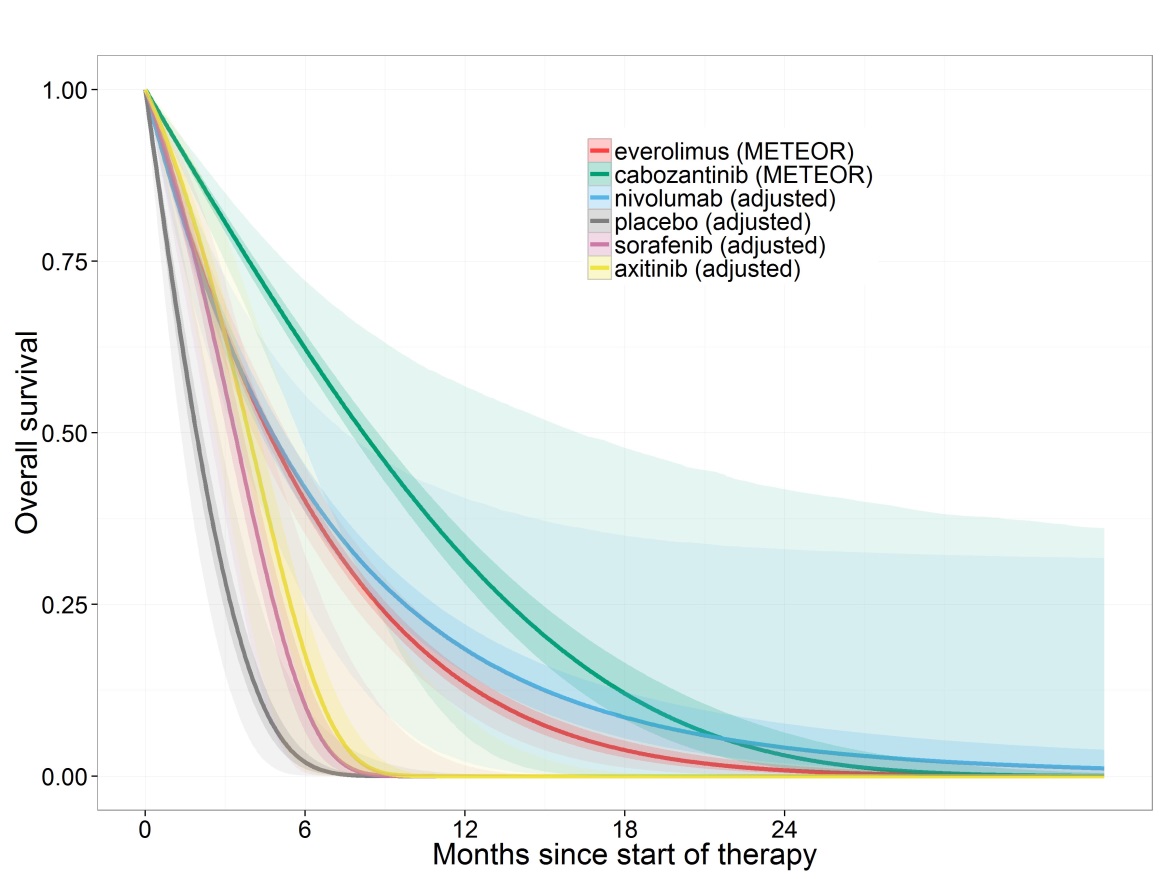
**

Fig 9. Averaged PFS over time derived from the Log-logistic random-effects model, adjusted to the baseline from METEOR study, with shaded areas representing 95% credible intervals.


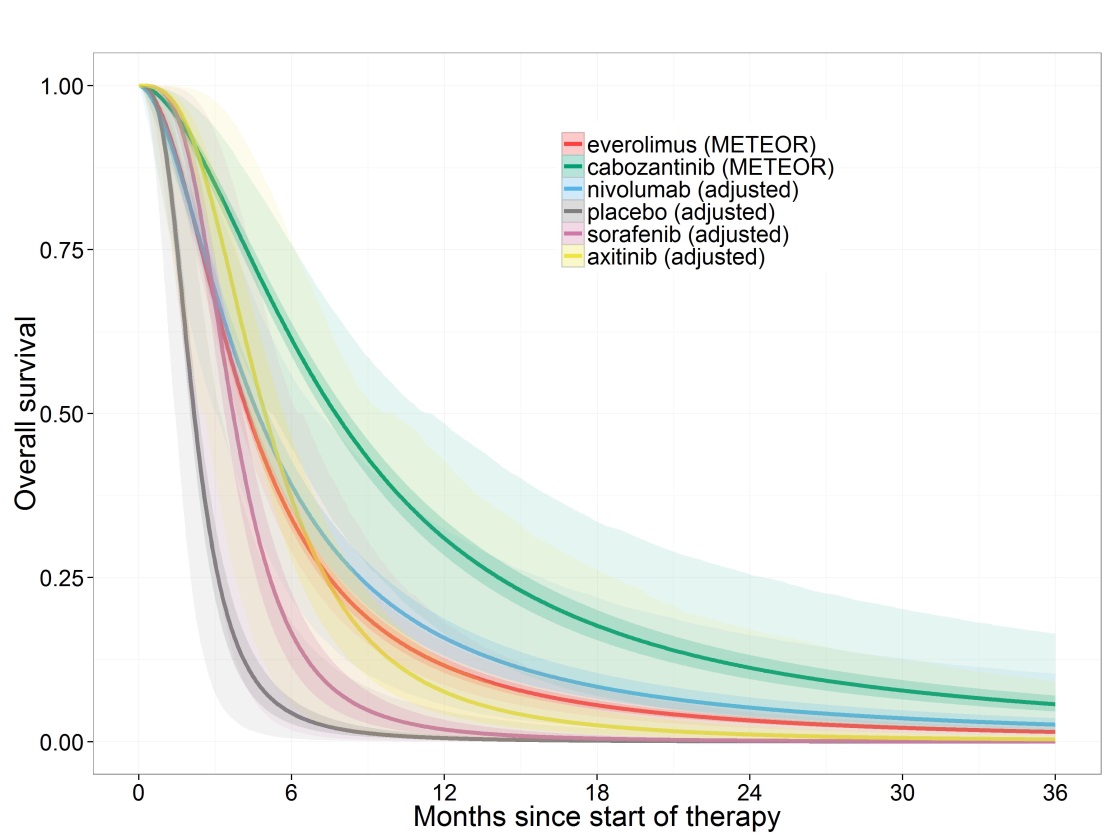


Fig 10. Averaged PFS over time derived from the exponential random-effects model, adjusted to the baseline from METEOR study, with shaded areas representing 95% credible intervals.

*
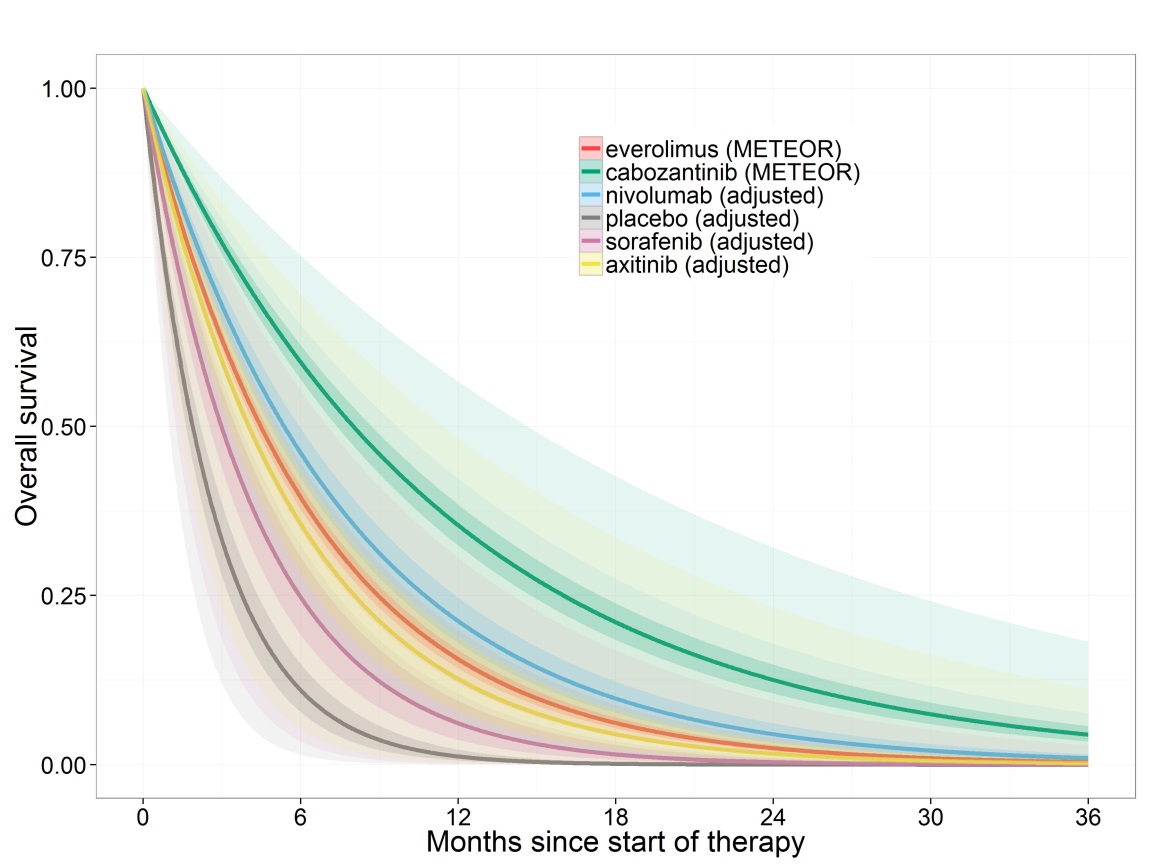
*

Fig 11. Averaged PFS over time derived from the lognormal random-effects model, adjusted to the baseline from METEOR study, with shaded areas representing 95% credible intervals.

*
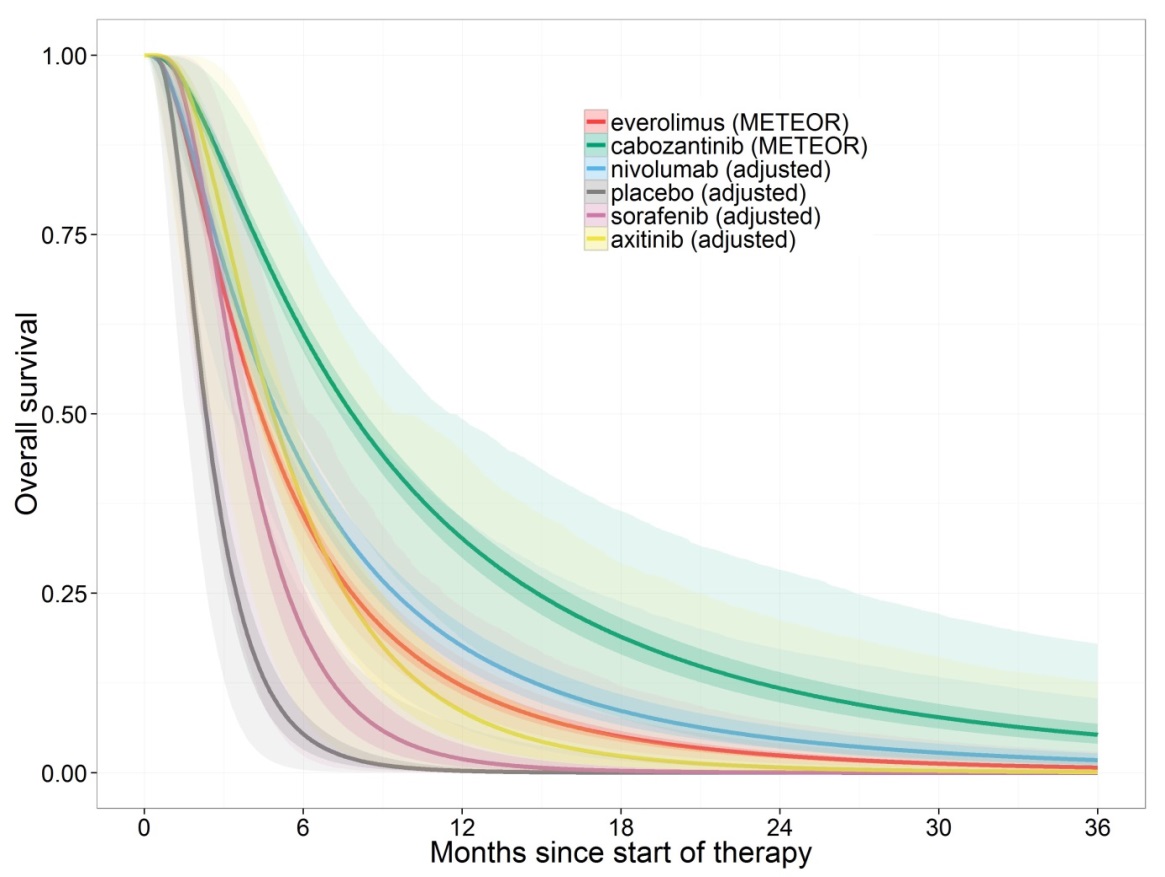
*

Fig 12. Averaged OS over time derived from the Weibull random-effects model, adjusted to the baseline from METEOR study, with shaded areas representing 95% credible intervals.


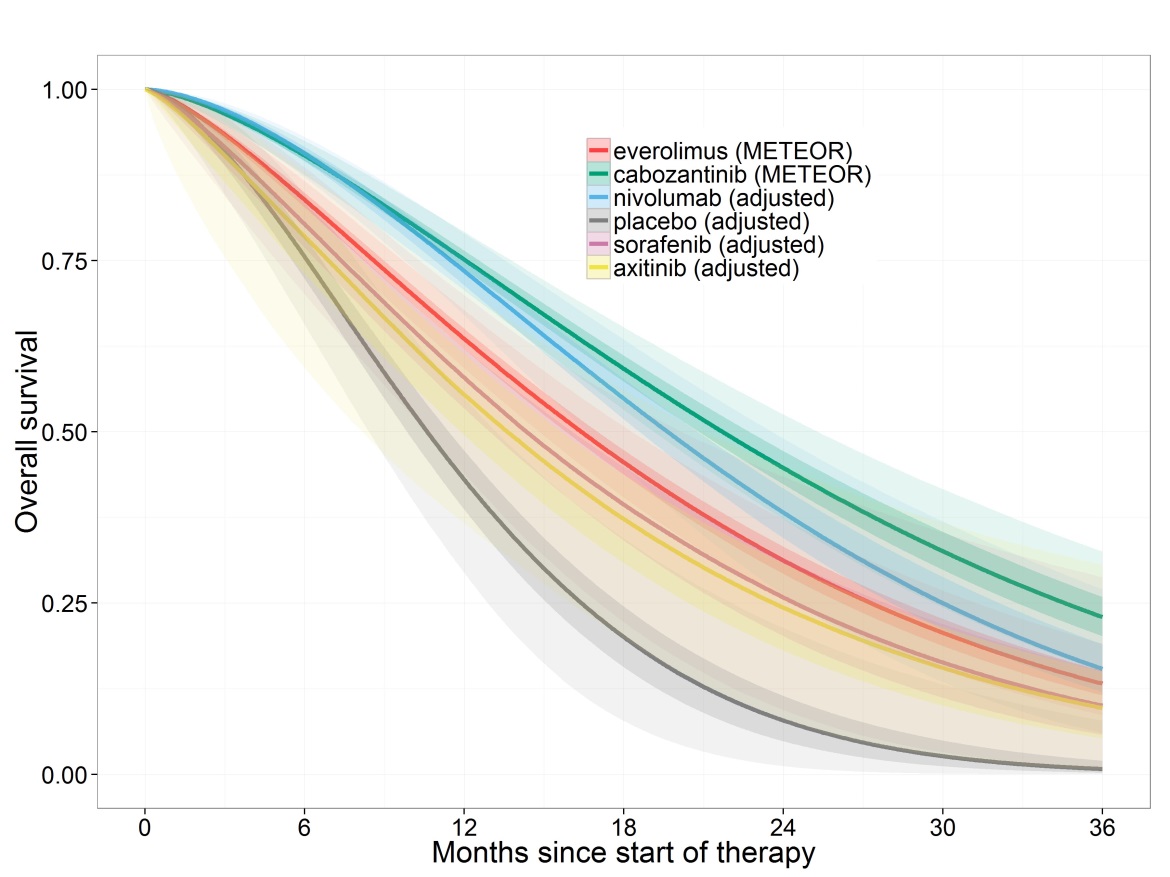


Fig 13. Averaged OS curves over time derived from the Gompertz random-effects model, adjusted to the baseline from METEOR study, with shaded areas representing 95% credible intervals.

**
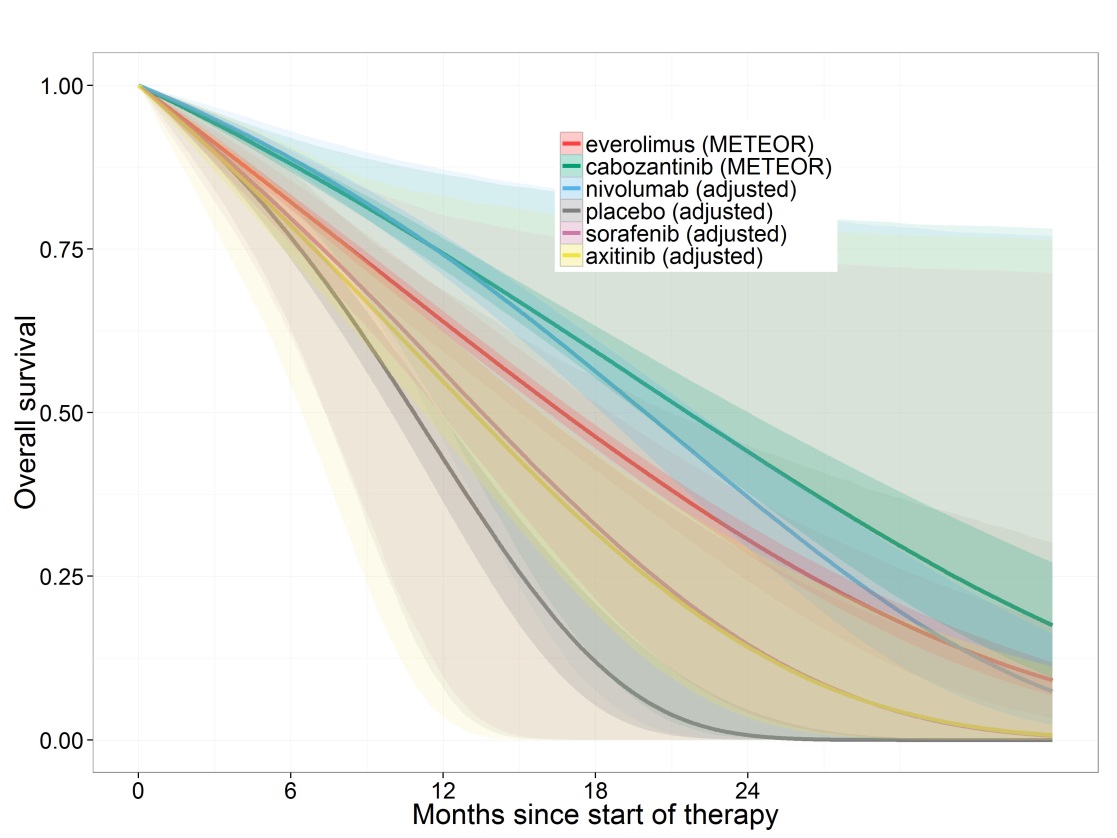
**

Fig 14. Averaged OS curves over time derived from the Log-logistic random-effects model, adjusted to the baseline from METEOR study, with shaded areas representing 95% credible intervals.


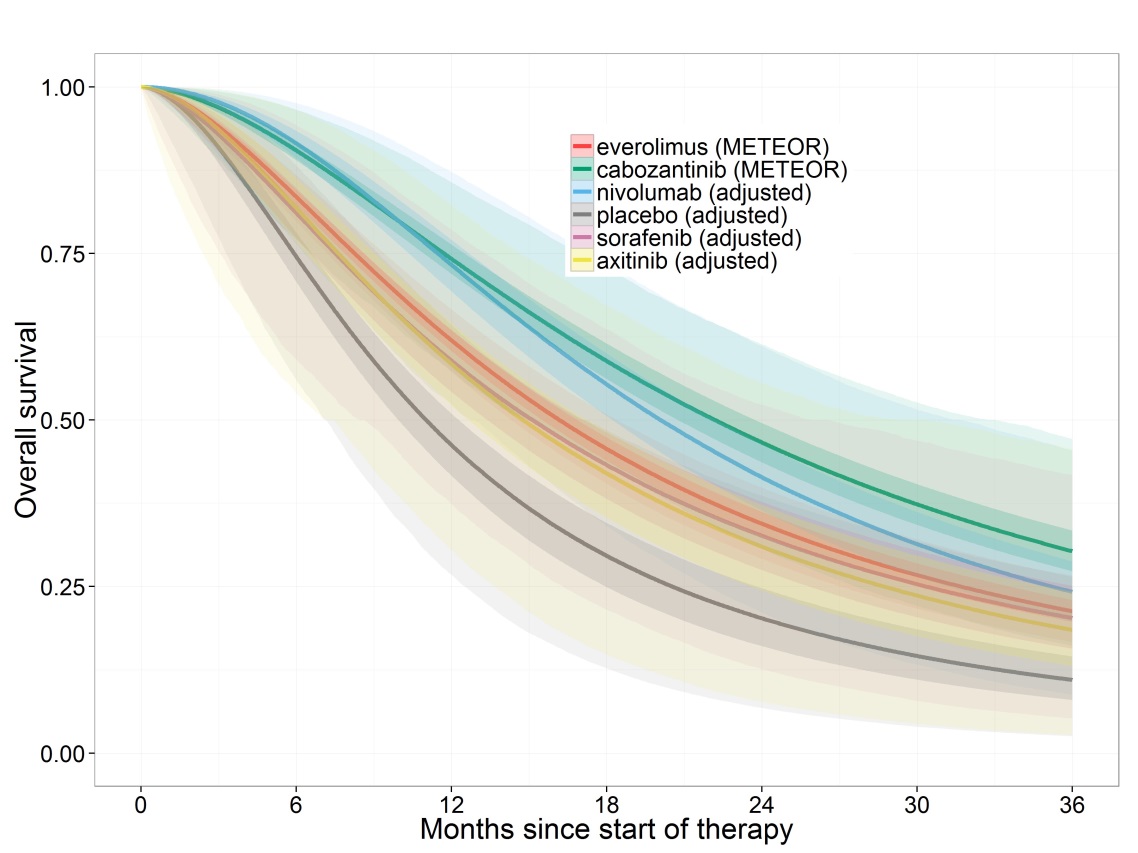


Fig 15. Averaged OS curves over time derived from the exponential random-effects model, adjusted to the baseline from METEOR study, with shaded areas representing 95% credible intervals.


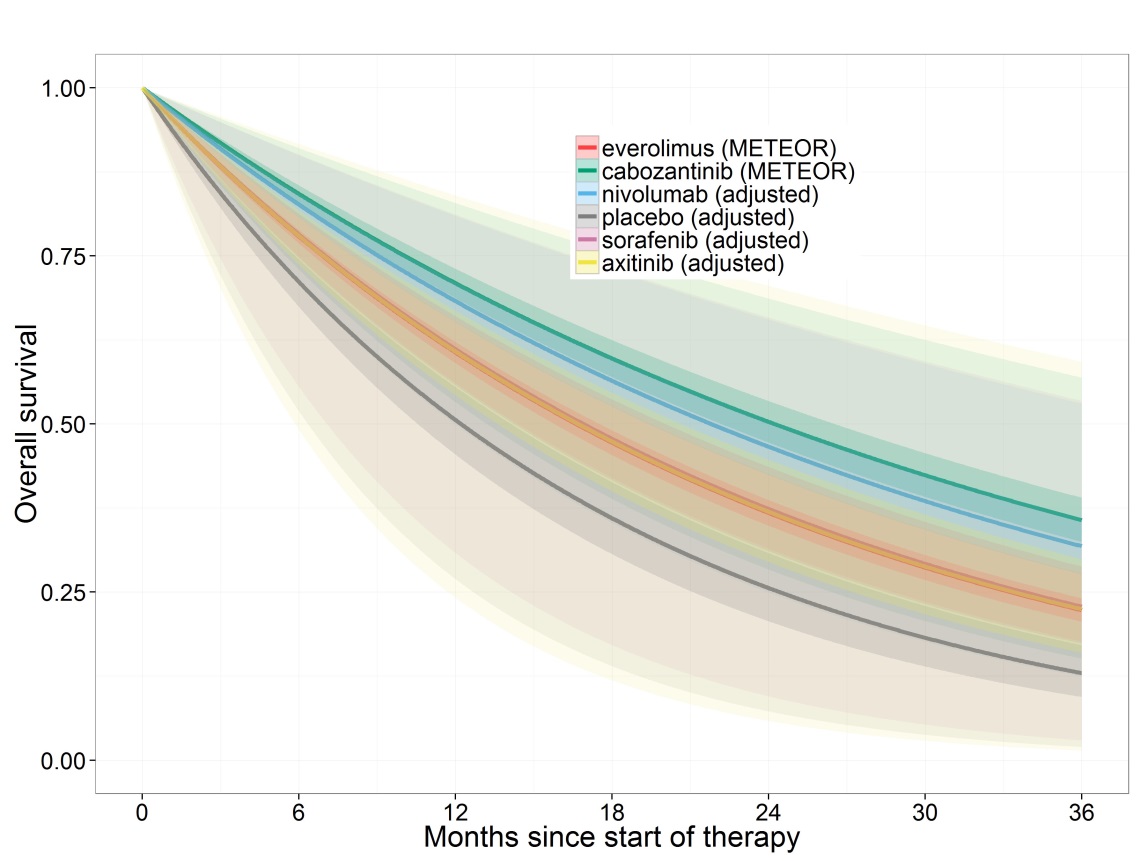


Fig 16. Averaged OS curves over time derived from the lognormal random-effects model, adjusted to the baseline from METEOR study, with shaded areas representing 95% credible intervals.


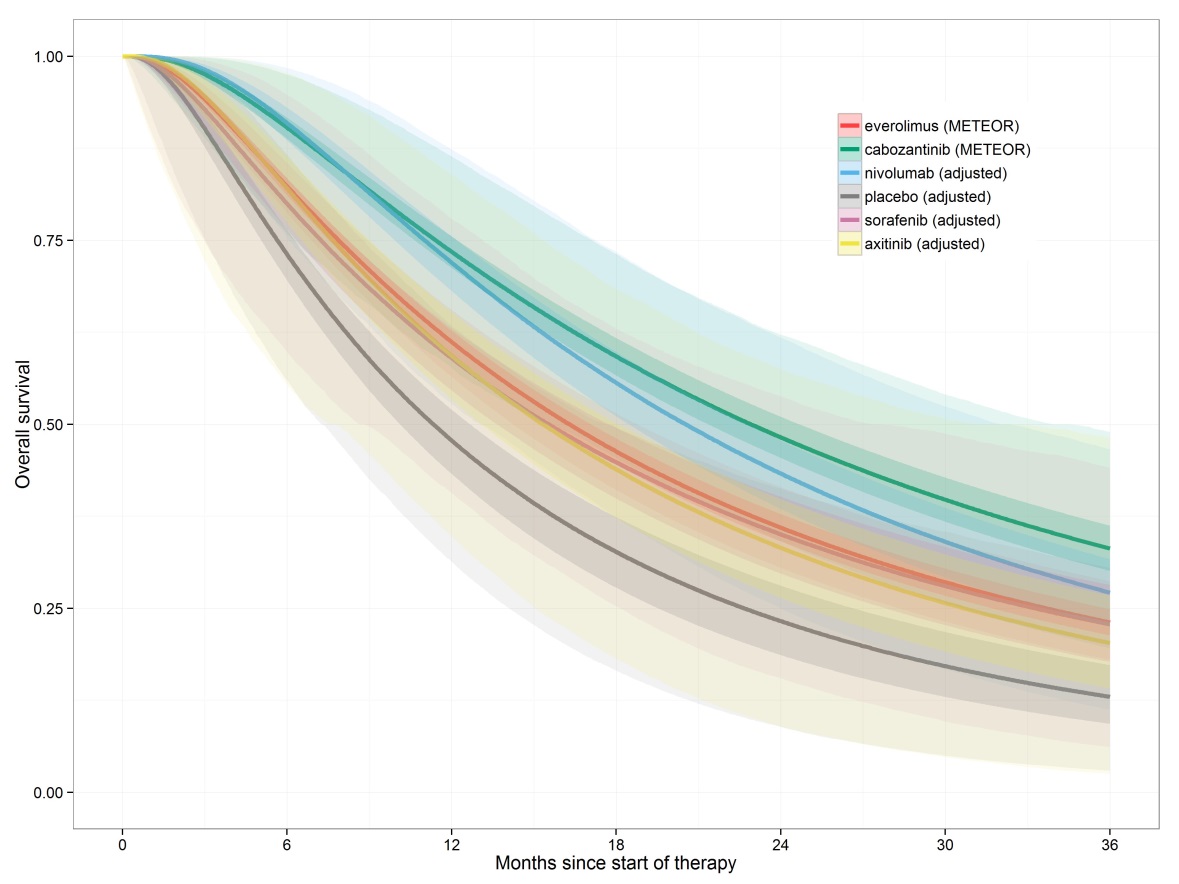

Supplement: S12 File — (DOCX) [file pone.0184423.s012.docx]
