# Supplementary material for: Cabozantinib versus everolimus, nivolumab, axitinib, sorafenib and best supportive care: A network meta-analysis of progression-free survival and overall survival in second line treatment of advanced renal cell carcinoma
Source: PLoS One. 2017 Sep 8;12(9):e0184423. doi: 10.1371/journal.pone.0184423 (PMC5590935; doi:10.1371/journal.pone.0184423)
Supplement: S13 File — (DOCX) [file pone.0184423.s013.docx]

**Table S13. Proportion of subsequent treatments received in different trials**

| **After progression of** | **Axitinib** | **Cabozantinib** | **Everolimus** | **Sunitinib** | **Sorafenib** | **Pazopanib** |
| --- | --- | --- | --- | --- | --- | --- |
| **Cabozantinib METEOR** | 17.0% | 0.0% | 29.0% | 5.2% | 0.0% | 0.0% |
| **Everolimus**  **METEOR** | 27.0% | 0.0% | 0.0% | 10.0% | 9.5% | 6.7% |
| **Axitinib**  **AXIS** | 0.5% | 0.0% | 39.0% | 8.5% | 16.0% | 8.5% |
| **Nivolumab**  **CheckMate025** | 24.2% | 0.0% | 25.6% | 6.8% | 6.3% | 9.0% |
